# Catalytic asymmetric synthesis of geminal-dicarboxylates†
†Electronic supplementary information (ESI) available. See DOI: 10.1039/c8sc01786g


**DOI:** 10.1039/c8sc01786g

**Published:** 2018-06-28

**Authors:** Nisha Mistry, Stephen P. Fletcher

**Affiliations:** a Department of Chemistry , Chemistry Research Laboratory , University of Oxford , 12 Mansfield Road , Oxford OX1 3TA , UK . Email: stephen.fletcher@chem.ox.ac.uk

## Abstract

Stereogenic acetals, spiroacetals and ketals are well-studied stereochemical features that bear two heteroatoms at a common carbon atom. These stereocenters are normally found in cyclic structures while linear (or acyclic) analogues bearing two heteroatoms are rare. Chiral geminal-dicarboxylates are illustrative, there is no current way to access this class of compounds while controlling the stereochemistry at the carbon center bound to two oxygen atoms. Here we report a rhodium-catalysed asymmetric carboxylation of ester-containing allylic bromides to form stereogenic carbon centers bearing two different carboxylates with high yields and enantioselectivities. The products, which are surprisingly stable to a variety of acidic and basic conditions, can be manipulated with no loss of enantiomeric purity as demonstrated by ring closing metathesis reactions to form chiral lactones, which have been extensively used as building blocks in asymmetric synthesis.

## Abstract 2

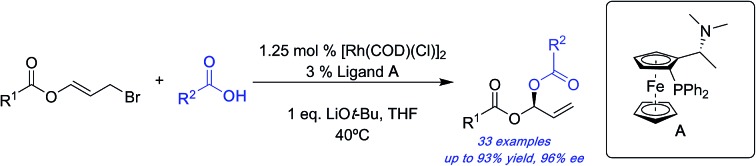
Here we report a rhodium-catalyzed asymmetric carboxylation of ester-containing allylic bromides to form stereogenic carbon centers bearing two different carboxylates with high yields and enantioselectivities.

## Introduction

**Fig. 1:**
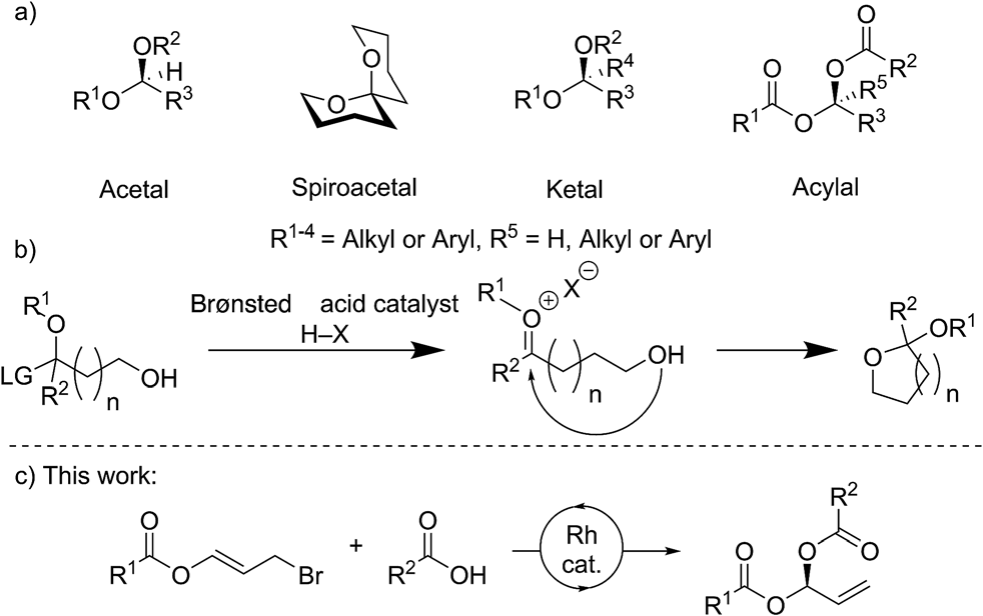
Common stereocenters bearing two heteroatoms and their catalytic asymmetric formation, including this work. (a) General structures for *O*,*O*-acetals, spiroacetals, ketals and dicarboxylates. (b) Formation of a bis-heteroatom bearing stereogenic center *via* an oxocarbenium ion intermediate using Brønsted acid catalysis. (c) This work: Rh catalyzed asymmetric carboxylation of heteroatom containing allyl bromides to form *gem*-dicarboxylates.

Stereocenters bearing two heteroatoms, including chiral acetals, spiroacetals and ketals (Fig. 1a), are some of the most prevalent structural motifs found in nature. These features are present in virtually all carbohydrate derivatives such as starch and cellulose, and in many small natural products including pheromones, steroids, and polyketides.^1^ The ability to control the stereochemistry of carbon centers featuring two heteroatoms is also important in the development of pharmaceuticals.^1^,^2^

For many years, stereocontrolled access to chiral acetal derivatives relied on derivatisation of chiral starting materials,^3^–^8^ metal mediated desymmetrisations,^9^–^11^ or kinetic or thermodynamically controlled cyclisation of carbonyl containing chiral-compounds.^12^–^17^ A recent approach to bis-heteroatom containing stereogenic centers involves catalysis with very sterically hindered chiral Brønsted acids. Though generally envisaged to occur *via* oxocarbenium ion or imine intermediates (Fig. 1b) which undergo stereoselective addition, some reactions likely proceed *via* single-step asynchronous pathways.^18^ This strategy has now been sufficiently developed to allow the synthesis of chiral *N*,*N*-,^19^–^22^ *N*,*O*-,^23^*N*,*S*-24,25 and *O*,*O*-acetals.^26^–^29^ Methods for the stereocontrolled formation of analogous linear compounds are less common.^19^,^20^,^23^,^24^

A variety of rhodium-catalysed asymmetric allylation-type processes, including allylic substitutions, with oxygen, nitrogen and carbon nucleophiles have been reported to form allylic alcohols, amines, and tertiary or quaternary carbon stereogenic centers.^30^–^39^ Carboxylic acids may be used as nucleophiles, not only in rhodium-catalysed asymmetric reactions,^40^ but also in processes catalysed by iridium,^41^ palladium,^42^,^43^ and ruthenium.^44^ However, only limited examples of metal-catalysed asymmetric additions to make stereogenic centres bearing two heteroatoms are known, and no Rh-catalysed processes have been reported.^45^–^48^

Here we report the stereocontrolled synthesis of geminal-dicarboxylates (acylals) *via* a highly enantioselective rhodium-catalysed carboxylation of allyl bromide derivatives bearing ester groups. These allyl bromides have been used in copper-catalysed additions of Grignard reagents to give allylic esters.^49^ The linear products described here feature a stereogenic carbon center bearing two different carboxylates. Geminal-dicarboxylates are an understudied class of compounds with the exception of the diacetate and dipropionate derivatives, which can protect aldehydes and are important substrates for asymmetric Tsuji–Trost reactions.^50^,^51^

Methods for the synthesis of 1,1-diacetates include protic and Lewis acid catalysis,^52^,^53^ the action of I_2_,^54^ NBS,^55^ and various heterogeneous catalysts.^56^ However, none of these methods allow stereocontrol. As far as we are aware the only report of asymmetric induction in *gem*-dicarboxylate formation involved copper-catalysed allylic oxidation of an olefin in 23% yield and 10% ee.^57^

## Results and discussion

Our standard reaction conditions involve 1.25 mol% [Rh(COD)(Cl)]_2_, 3 mol% of Ugi amine derived ligand **A**, 1 eq. of LiO*t*-Bu and THF at 40 °C. Using ester substituted allyl bromide **1a**, easily prepared by mixing benzoyl bromide and acrolein at room temperature in CH_2_Cl_2_,^58^ we are able to add isobutyric acid to give **2a** in good yield and excellent ee (82%, 96% ee, Table 1, entry 1).

**Table 1:** Asymmetric geminal-dicarboxylation and variations from standard conditions

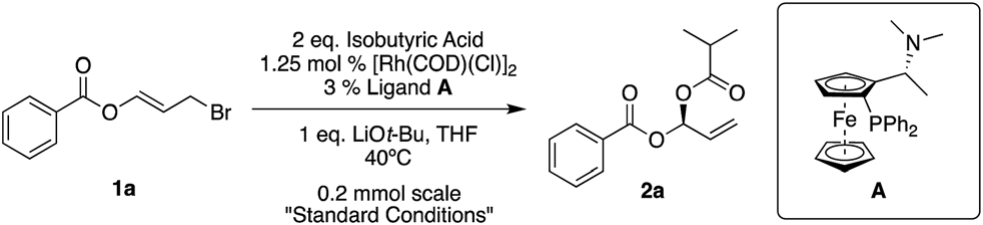				
Entry	Variation from standard conditions	Reaction time	Yield^*a*^ (%)	ee^*b*^ (%)
1	None	1.5 h	82	96
2	No Rh	o/n^*c*^	0	—
3	No ligand	o/n	76	*rac*
4	(*S*)-BINAP instead of **A**	o/n	69	45
5	No LiO*t*-Bu	o/n	0	—
6	LiOMe instead of LiO*t*-Bu	1 h	85	96
7	KO*t*-Bu instead of LiO*t*-Bu	o/n	67	8
8	Room temperature	2 h	89	94
9	60 °C	1 h	83	95
10	1 eq. isobutyric acid	50 min	82	96
11^*d*^	4 mmol scale	2 h	82	95

^*a*^All yields are isolated yields.

^*b*^Enantiomeric excesses determined by SFC using a chiral non-racemic stationary phase.

^*c*^The reaction was stirred overnight.

^*d*^Gram-scale reaction, carried out using 0.5 mol% [Rh(COD)(Cl)]_2_ and 1.2 mol% ligand.

Pleasingly, we also observe complete regioselectivity for the S_N_2′ product over the S_N_2 product, which is a known challenge in allylic substitution reactions (see ESI† for further details).

If we remove either the rhodium source or base from the reaction, we obtain no product (Table 1, entries 2 and 5). A reaction without ligand **A** gave racemic product (Table 1, entry 3) and using (*S*)-BINAP instead of **A** gave 45% ee (Table 1, entry 4).

As long as it is of high quality, it is possible to use LiOMe instead of LiO*t*-Bu (Table 1, entry 6), however when switching to non-Li bases, for example KO*t*-Bu, the ee drops significantly (8% ee, Table 1, entry 7).

Room temperature reactions proceed with excellent results but to maintain reaction component solubility, adequate stirring and reasonable reaction times (particularly when using other nucleophiles), 40 °C appears to be a suitable temperature (Table 1, entries 8 and 9). The reaction can easily be performed on a gram-scale while simultaneously reducing the amount of rhodium to 0.5 mol% [Rh(COD)(Cl)]_2_, and ligand to 1.2 mol% (Table 1, entry 11).

**Fig. 2:**
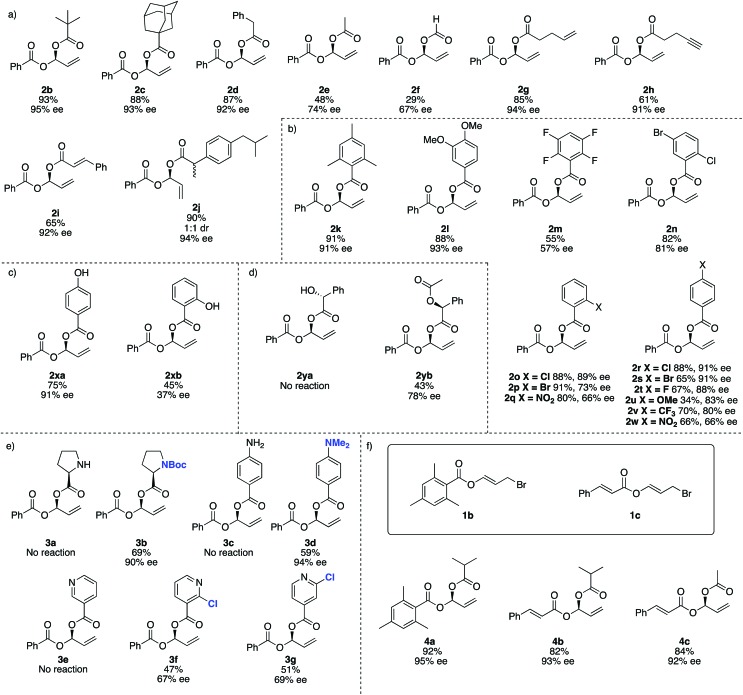
Reaction scope. (a) Scope of aliphatic carboxylic acids. (b) Scope of aromatic carboxylic acids. (c) 4-Hydroxybenzoic acid and 2-hydroxybenzoic acid give very different results. (d) Protection of free –OH groups may allow an otherwise problematic reaction to occur. (e) Nitrogen-containing and pyridyl substrates. (f) Using different carboxylic acid derived electrophiles. All reactions were carried out on a 0.4 mmol scale of the electrophile. All yields are isolated yields. Enantiomeric excesses determined by SFC using a chiral non-racemic stationary phase.

For reaction scope, aliphatic carboxylic acids including bulky *tert*-butyl (**2b**) and adamantyl groups (**2c**), provided the corresponding products in excellent enantioselectivities (>93% ee), however smaller nucleophiles such as formic and acetic acid (**2e** and **2f** respectively), give lower yields and ee's. In addition to the reduced yield and ee, the reaction with formic acid also gave small amounts of the achiral dibenzoyloxy derivative of **2** and under some conditions **1a** may decompose to benzoic acid, which then undergoes competitive Rh-catalysed carboxylation to the remaining **1a**. We were not able to separate this by-product from **2f** (Fig. 2).

This method is compatible with carboxylic acids that contain a terminal alkene (**2g**), terminal alkyne (**2h**) and an internal alkene (**2i**). When racemic ibuprofen is used as a nucleophile, a 1 : 1 mixture of diastereoisomers is formed (both diastereomers having 94% ee, **2j**).

Aromatic carboxylic acids generally work well as nucleophiles. 2,4,6-Trimethylbenzoic acid (**2k**) and 4,5-dimethoxybenzoic (**2l**) both gave high yields and ee's over 90%. 4-Chloro- (**2r**), 2-chloro- (**2o**) and 4-bromobenzoic acid (**2s**) also give good ee's. Interestingly the 2-bromo derivative (**2p**) was formed in excellent yield (91%) but the ee drops to only 73%. Increasing the electron withdrawing potential of ring substituents, for example having fluoro (**2r**), trifluoromethyl (**2v**), nitro (**2q** and **2w**) or multiple halogens (**2m** and **2n**) tends to decrease the yield and ee. Curiously 4-methoxybenzoic acid (**2u**) gives only a 34% yield and 83% ee, whereas 4,5-dimethoxybenzoic acid (**2l**) gave 88% yield and 93% ee.

For 4-hydroxybenzoic acid (**2xa**) we obtained good results (75%, 91% ee) but a hydroxy at the 2-position (**2xb**) is detrimental (45%, 37% ee), likely due to either chelation of **2xb** to Rh during the reaction or hydrogen (or lithium) bonding of the 2-hydroxy group altering the nucleophilicity of the carboxylic acid. A free hydroxyl group in (*R*)-2-hydroxy-2-phenylacetic acid (used as a single enantiomer) entirely suppressed reactivity and no product **2ya** is observed, but if acetylated (here the single *S*-enantiomer was arbitrarily used) **2yb** can be obtained (43%, 78% ee).

Substrates containing free amino groups such as enantiomerically pure proline (**3a**) and 4-aminobenzoic acid (**3c**) give no product, but protected derivatives (single enantiomer Boc-proline and *N*,*N*-dimethyl-4-aminobenzoic acid) allowed synthesis of **3b** and **3d** in respectable yield with excellent enantioselectivity (>90% ee). **3b** was obtained as a ‘single diastereoisomer’ which exists as a 3 : 1 mixture of rotamers at room temperature, as observed by NMR spectroscopy (see ESI† for NOESY spectra). Nicotinic acid did not give product **3e**, but addition of a chloro group in the 2-position of the pyridyl ring allowed moderate yields and enantioselectivities to be achieved in formation of **3f** and **3g**. This observation is consistent with previous Rh-catalysed asymmetric processes,^59^ and overall these experiments suggest that many other carboxylic acid bearing heterocycles and heteroatoms would be compatible with this method if appropriate protecting group strategies are used.

We then examined different allylic bromides **1b** and **1c**^58^ with isobutyric acid which gave **4a** and **4b** with good yields and excellent ee's. We note that acetic acid in combination with **1a** gave 48% yield and 74% ee however with **1c** much better results (84%, 92% ee) are observed.

**Fig. 3:**
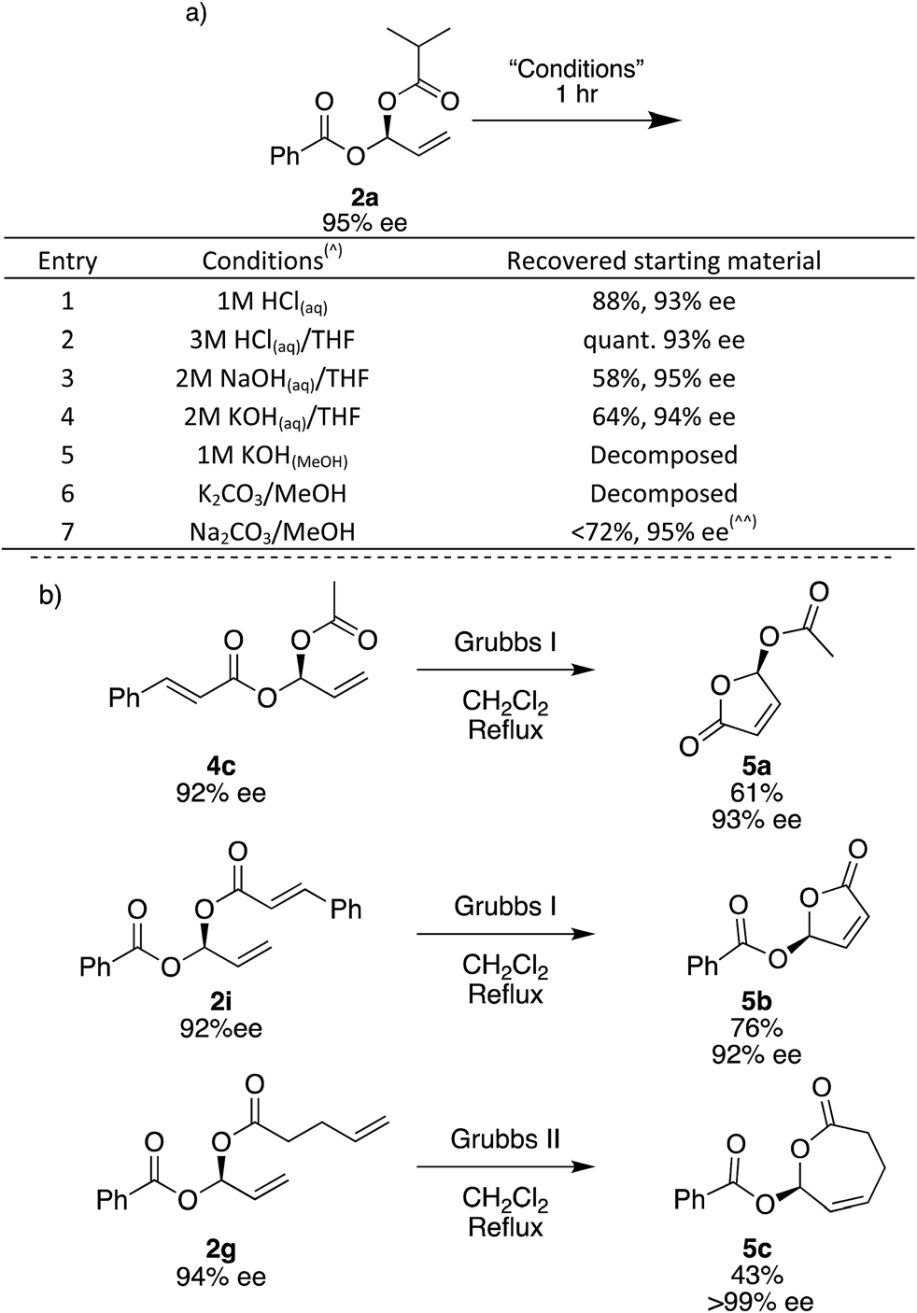
Stability and derivatization of *gem*-dicarboxylates. (a) (^) 25 mg of **2a** stirred in 1 mL of a 1 : 1 mixture of solvents for 1 hour. (^^) Complete decomposition observed overnight. (aq) – queous, (MeOH) – methanolic solution. (b) Ring closing metathesis of *gem*-dicarboxylates to lactones. Isolated yields. Enantiomeric excesses determined by SFC.

To investigate the stability of the *gem*-dicarboxylates, we subjected **2a** to various conditions for 1 hour at room temperature. **2a** is remarkably stable to a range of conditions; in aqueous acidic solutions of up to 3 M HCl, there is a negligible loss in the yield and ee of **2a** (Fig. 3a, entries 1 and 2). In aqueous basic solutions of up to 2 M NaOH or KOH we see some loss of yield, there is no change in ee (Fig. 3a, entries 1–4). Unsurprisingly, the products were unstable to methanolic basic solutions in combination with potassium salts, and under these conditions complete decomposition was observed (Fig. 3a, entries 5 and 6). Using Na_2_CO_3_ and MeOH trace decomposition was observed after 1 h, and complete decomposition occurs overnight (Fig. 3).

The *gem*-dicarboxylates shown here have not previously been described in the literature and since there has been no good way to access these chiral compounds before, their chemistry has not yet been explored. In order to demonstrate if the *gem*-dicarboxylates may be useful we briefly examined their conversion to other species using ring-closing metathesis. The synthesis of cyclic small-ring esters is of considerable interest as they frequently appear in natural products and show important biological activity.^60^ Asymmetric γ-butenolides have been widely studied but only acetyl esters in the γ-position have been reported, and asymmetry is normally induced using enzymes.^61^–^64^

Using the terminal alkene formed during the Rh-catalysed addition, we are able to access 5- and 7-membered lactones. Treatment of **4c** and **2i** with the 1^st^ generation Grubbs catalyst in refluxing CH_2_Cl_2_ overnight gave 5-membered lactones **5a** and **5b**, which have a γ-stereogenic center. We assign the absolute stereochemistry of all *gem*-dicarboxylate products here, including diastereomeric mixture **2j**, and single diastereoisomers **2yb** and **3b**, by comparing the optical rotation of lactone **5a** with that quoted in the literature.^62^ This is the first report of an asymmetric synthesis of compound **5b**.^65^ Using electrophile **1c** specifically, the combination of Rh-catalysed carboxylation followed by RCM has the potential to give access to a range of new chiral γ-butenolides. Attempts to use the Grubbs I catalyst to form a 7-membered ring did not give the desired **5c**, but the 2nd generation Grubbs catalyst gave 43% yield and **5c** was obtained as a solid with >99% ee.

## Conclusions

In conclusion, we have developed a method to form chiral *gem*-dicarboxylates by Rh-catalysed asymmetric carboxylation. Many different carboxylic acid nucleophiles can be used to give novel *gem*-dicarboxylates in good yields with high enantioselectivity. The products are remarkably stable to a variety of acidic and basic conditions. We have demonstrated that the products can be used to form other chiral acetal derivatives using the terminal alkene formed in the reaction in subsequent RCM reactions to access novel cyclic products including valuable γ-butenolides. We anticipate that these *gem*-dicarboxylates may have many other potential uses and now that we have described an efficient synthesis, this chemistry can now be explored more fully.

More generally, linear products, chiral by virtue of a stereogenic carbon bearing two differentiated hetereoatoms, can be obtained by metal catalysed asymmetric additions to appropriately substituted electrophiles. The ease of synthesis and stability of the products suggests that a broad range of new chemical species may be accessible by developing strategies to form stereogenic carbon centers featuring different combinations of hetereoatoms.

## Experimental procedures

### General procedure for asymmetric carboxylation reaction to give chiral *gem*-dicarboxylates

In a flame-dried 10 mL round bottomed flask [Rh(COD)(Cl)]_2_ (2.5 mg, 0.0050 mmol, 0.0125 eq.), ligand **A** (5.3 mg, 0.012 mmol, 0.030 eq.) and LiO*t*-Bu (32 mg, 0.40 mmol, 1.0 eq.) were stirred in THF (2 mL) at 60 °C for 30 min. The reaction was cooled to 40 °C then a solution of the allylic bromide (**1a–c**, 0.40 mmol, 1.0 eq.) and the carboxylic acid (0.80 mmol, 2.0 eq.) in THF (1.5 mL) was added *via* syringe and the flask rinsed with THF (0.5 mL). The resulting mixture was then stirred at 40 °C until the reaction was complete by TLC. SiO_2_ was added and the solvent was then carefully evaporated. The resulting solid was directly loaded onto a chromatographic column and eluted with Et_2_O/pentane to afford the products.

## Conflicts of interest

There are no conflicts to declare.

## Supplementary Material

Supplementary information
