# Supplementary material for: Catalytic asymmetric synthesis of geminal-dicarboxylates
Source: Chem Sci. 2018 Jun 28;9(29):6307–12. doi: 10.1039/c8sc01786g (PMC6063137; doi:10.1039/c8sc01786g)
Supplement: Supplementary file 1 [file SC-009-C8SC01786G-s001.pdf]

Electronic Supplementary Material (ESI) for Chemical Science.
This journal is © The Royal Society of Chemistry 2018

Supporting Information

Catalytic asymmetric synthesis of *geminal*-dicarboxylates

Nisha Mistry and Stephen P. Fletcher

General Methods

The reactions were carried out in glassware that was flame-dried under vacuum, in anhydrous solvents with continuous magnetic stirring in an inert argon atmosphere. Cooling to 0 °C was achieved with an ice/water bath and to –78 °C, was achieved with an acetone/dry ice bath. If needed, other temperatures were obtained using a Julabo FT902 immersion cooler. Heating was performed using DrySyn heating blocks.

Thin layer chromatography was performed on plates with glass backing (Silica Gel 60 F_{254}, Merck or Aluminium Oxide N/UV_{254}, Macherey–Nagel) and visualised with UV light (254 nm) and aqueous potassium permanganate stain. Column chromatography was performed by using Merck 60 Å silica gel or Acros 60 Å aluminium oxide, neutral and pressure applied with a flow of nitrogen.

Unless otherwise stated, nuclear magnetic resonance (NMR) spectroscopy measurements were carried out at room temperature. ^{1}H NMR, ^{13}C NMR, ^{19}F NMR, DEPT-135, COSY, HSQC, HMBC and NOESY experiments were carried out using Bruker AVN-400 (400/100 MHz), DQX-400 (400/100 MHz) or AVC-500 (500/125 MHz) spectrometers.

Chemical shifts (δ) are reported in ppm relative to the residual solvent peak with corresponding coupling constants (*J*) in Hertz (Hz) and multiplicities (s: singlet, d: doublet, t: triplet, q: quartet, m: multiplet).

Infrared (IR, neat, thin film) spectroscopy was carried out on a Bruker Tensor 27 FT–IR spectrometer with in internal calibration range of 4000 – 600 cm^{-1}.

Optical rotations were recorded on a Perkin-Elmer 241 polarimeter at 20 °C in a 10 cm cell in the stated solvent; [α] D values are given in 10–1 deg.cm2 g–1 (concentration c given as g/100 mL).

Low-resolution mass spectra were recorded using a Walters LCT premier XE. High Resolution Mass spectra were carried out by internal service at the university of Oxford. (1) Electron spray ionisation (ESI+) were recorded on a Fisons Platform II. (2) Electron ionisation (EI)/Chemical ionisation (CI): Analyses were performed on an Agilent 7200 quadrupole time of flight (Q-ToF) instrument equipped with a direct insertion probe supplied by Scientific instrument Manufacturer (SIM) GmbH. Instrument control and data processing were performed using Agilent MassHunter software. The system was calibrated on the day of the analysis and its mass accuracy with external calibration (as used for these experiments) is better than 5ppm for 24 hours following calibration. Source conditions for both EI and CI were adjusted to maximise sensitivity, the reagent gas used in CI was either methane or ammonia (and should be apparent in the metadata associated with the data). (3) Atmospheric pressure chemical ionisation (APCI+): Analyses were performed using a Thermo Exactive mass spectrometer equipped with Waters Acquity liquid chromatography system. Instrument control and data processing were performed using Thermo Xcalibur Software. The system was calibrated on the day of the analysis and its mass accuracy with external calibration (as used for these experiments) is better than 5ppm for 24 hours following calibration. The mass spec was operated using the APCI probe and resolution was set to 50,000. APCI source conditions were adjusted to maximise sensitivity. A mixture of 10% water, 89.9% methanol and 0.1% formic acid was used to transport samples to the mass spectrometer at a flow rate of 0.2 mL/min. The values of mass over charge (*m/z*) indicate the most intense peak.

Optical rotations ($[\alpha]_D^{20}$) were recorded using a Perkin Elmer- 241 Polarimeter. Concentrations (*c*) are reported in g/100 mL.

Chiral HPLC separations were achieved using an Agilent 1230 Infinity series normal phase HPLC unit and HP Chemstation software. Chirapak® columns (250 x 4.6 mm), fitted with matching Chirapak® Guard Cartridges (10 x 4 mm), were used as specified in the text. Solvents used were of HPLC grade (Sigma Aldrich); all eluent systems were isocratic.

Chiral SFC (supercritical fluid chromatography) separations were conducted on a Waters Acquity UPC2 system using Waters Empower software. Chiralpak® columns (150 × 3 mm, particle size 3 μm) were used as specified in the text. Solvents used were of HPLC grade (Fisher Scientific, Sigma Aldrich or Rathburn).

Chemicals

Commercially available reagents were purchased from Sigma Aldrich, Alfa Aesar, Acros Organics, Flurochem and Strem Chemicals and unless otherwise stated were used without further purification. Dry solvents were collected fresh from an mBraun SPS–800 solvent purification system after having passed through anhydrous alumina columns. Deuterated solvents were purchased from Sigma Aldrich. Pentane (HPLC grade) was used without further purification.

Screening

Table 1. Screening and optimisation table

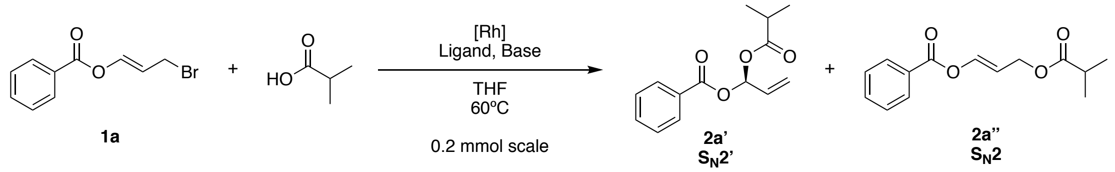

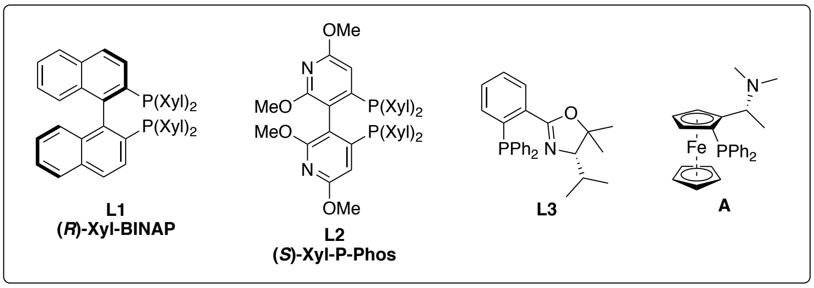

Entry	[Rh]	Ligand	Base	Reaction Time	Yield[a] (%)	2a':2a"[b]	ee[c] (%)
1	$[Rh(coe)Cl]_2$	L1	Na_2CO_3	1.5 hrs	80	>99.9:0.1	–13
2	$[Rh(coe)Cl]_2$	L2	KO*t*-Bu	1.5 hrs	72	85:15	13
3	$[Rh(coe)Cl]_2$	L2	LiOMe	21 hrs	71	>99.9:0.1	57
4	$[Rh(coe)Cl]_2$	L3	KO*t*-Bu	1.5 hrs	50	70:30	16
5	$[Rh(cod)Cl]_2$	L2	LiOMe	1 hr	70	>99.9:0.1	65
6	$[Rh(cod)Cl]_2$	A	LiOMe	1 hr	85	>99.9:0.1	96
7	$[Rh(cod)Cl]_2$	A	LiO*t*-Bu	1 hr	83	>99.9:0.1	95
8[d]	$[Rh(cod)Cl]_2$	A	LiO*t*-Bu	1.5 hrs	82	>99.9:0.1	96

[a] All yields are isolated yields [b] Regioselectivity determined by ^1H NMR spectroscopy [c] Enantiomeric excesses of S_N2' product determined by SFC using a chiral non-racemic stationary phase [d] Reaction carried out at 40 °C

Experimental Procedures

(*E*)-3-Bromoprop-1-en-1-yl benzoate (1a)

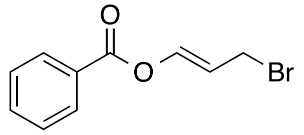

Prepared according to the procedures of Trombini and Lombardo *et al.*[1] Benzoyl bromide and acrolein were freshly distilled. Under an argon atmosphere, acrolein (7.74 mL, 116 mmol, 1.0 eq.) was added to CH_2Cl_2 (115 mL) and the resulting solution cooled to 0 °C. Benzoyl bromide (21.44 g, 116 mmol, 1.0 eq.) was added before stirring the reaction at room temperature for 72 hours. The reaction was concentrated *in vacuo* to give an oil. The pure product was recrystallized from pentane to give a white crystalline solid (11.31 g, 40% yield)

^{1}H NMR (400 MHz, $CDCl_3$) δ = 8.13 – 8.07 (m, 2H), 7.72 – 7.65 (m, 1H), 7.62 (td, *J*=7.2, 1.4, 1H), 7.48 (dd, *J*=8.4, 7.1, 2H), 5.90 (dt, *J*=12.4, 8.4, 1H), 4.07 (dd, *J*=8.4, 1.0, 2H).
^{13}C NMR (101 MHz, $CDCl_3$) δ = 163.2, 139.4, 133.9, 130.1, 128.6, 128.4, 111.9, 28.6.
HRMS (ESI) *m/z* calcd for $C_{10}H_9BrO_2$ $[M]^+$: 239.9786, found: 239.9574
IR (ATR) ν (cm^{-1}, $CHCl_3$): 705, 937, 1068, 1160, 1294, 1319, 1732
m.p.: 72–75°C

(*E*)-3-bromoprop-1-en-1-yl 2,4,6-trimethylbenzoate (1b)

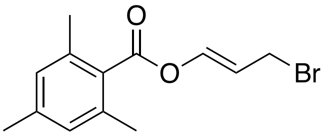

Synthesized using the same procedure as for **1a** from the corresponding acid bromide and acrolein, both freshly distilled. The pure product was recrystallized from pentane to give a white crystalline solid (1.02 g, 30% yield)

^{1}H NMR (400 MHz, $CDCl_3$) δ 7.67 (dt, *J* = 12.4, 1.1 Hz, 1H), 6.88 (d, *J* = 1.1 Hz, 2H), 5.79 (dt, *J* = 12.3, 8.4 Hz, 1H), 4.04 (dd, *J* = 8.4, 1.0 Hz, 2H), 2.32 (s, 6H), 2.30 (s, 3H).
^{13}C NMR (101 MHz, $CDCl_3$) δ 166.3, 140.4, 139.2, 136.2, 128.7, 111.8, 28.4, 21.2, 20.1.
HRMS (GCMS with methane as reagent gas) *m/z* calcd. for $C_{13}H_{16}O_2Br$ $[M+H]^+$: 282.0328, found: 283.0320
IR (ATR) ν (cm^{-1}, $CHCl_3$): 930, 1166, 1246, 1735, 2360
m.p.: 84–86°C

(*E*)-3-bromoprop-1-en-1-yl cinnamate (1c)

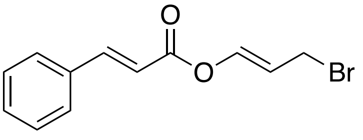

Synthesized using the same procedure as for **1a** from the corresponding acid bromide and acrolein, both freshly distilled. The pure product was recrystallized from pentane to give a white crystalline solid (1.74 g, 46% yield)

^{1}H NMR (400 MHz, $CDCl_3$) δ 7.81 (d, *J* = 16.0 Hz, 1H), 7.63 – 7.50 (m, 3H), 7.48 – 7.36 (m, 3H), 6.45 (d, *J* = 16.0 Hz, 1H), 5.80 (dt, *J* = 12.4, 8.4 Hz, 1H), 4.04 (dd, *J* = 8.4, 1.1 Hz, 2H)
^{13}C NMR (101 MHz, $CDCl_3$) δ 163.4, 147.3, 139.4, 133.9, 131.0, 129.0, 128.4, 116.1, 111.4, 28.7.
HRMS (GCMS with methane as reagent gas) *m/z* calcd. for $C_{12}H_{12}O_2Br$ $[M+H]^+$: 267.0015, found: 267.0011
IR (ATR) ν (cm^{-1}, $CHCl_3$): 763, 976, 1139, 1167, 1633, 1728, 2361
m.p.: 98–100°C

General procedure for racemic *gem*-dicarboxylates: In a flame-dried 5 mL round bottomed flask $[Rh(cod)(Cl)]_2$ (1.2 mg, 0.0025 mmol, 0.0125 eq), (±)-BINAP (2.6 mg, 0.006 mmol, 0.03 eq) and LiO*t*-Bu (16 mg, 0.20 mmol, 1.00 eq) were stirred in THF (1 mL) at 60 °C for 30 min. A solution of the allylic bromide (**1a-c**, 0.20 mmol, 1.00 eq) and the carboxylic acid (0.40 mmol, 2.00 eq) in THF (0.75 mL) was then added via syringe and the flask rinsed with THF (0.25 mL). The resulting mixture was then stirred at 60 °C until the reaction was complete by TLC. SiO_2 was added and the solvent was then carefully evaporated. The resulting solid was directly loaded onto a chromatographic column and eluted with Et_2O/pentane to afford the products.

General procedure for asymmetric *gem*-dicarboxylates:

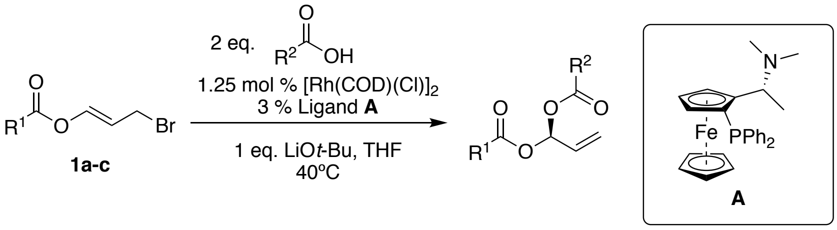

In a flame-dried 10 mL round bottomed flask $[Rh(cod)(Cl)]_2$ (2.5 mg, 0.005 mmol, 0.0125 eq), Ligand **A** (5.3 mg, 0.012 mmol, 0.03 eq) and LiO*t*-Bu (32 mg, 0.40 mmol, 1.00 eq) were stirred in THF (2 mL) at 60 °C for 30 min. The reaction was cooled to 40 °C then a solution of the allylic bromide (**1a-c**, 0.40 mmol, 1.00 eq) and the carboxylic acid (0.80 mmol, 2.00 eq) in THF (1.5 mL) was added *via* syringe and the flask rinsed with THF (0.5 mL). The resulting mixture was then stirred at 40 °C until the reaction was complete by TLC. SiO_2 was added and the solvent was then carefully evaporated. The resulting solid was directly loaded onto a chromatographic column and eluted with Et_2O/pentane to afford the products.

(*R*)-1-(isobutyryloxy)allyl benzoate (2a)

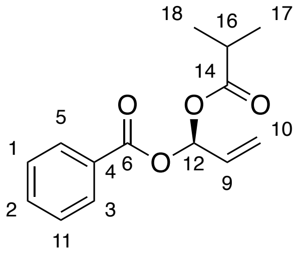

Reaction time: 1.5 hrs. Purification: 0–5% Et_2O in Pentane. Colourless oil (81.4 mg, 82% yield, 96% ee)

The enantiomeric excess of 96% was determined by SFC [Chiralpak® ID; 1500 psi, 30°C; 1% to 30% MeOH in 5 mins; flow: 1.5 ml/min; λ=225 nm; minor enantiomer t_R = 1.35 mins, major enantiomer t_R = 1.42 mins]

^{1}H NMR (400 MHz, $CDCl_3$) δ 8.13 – 8.02 (m, 2H, Ar), 7.65 – 7.54 (m, 1H, Ar), 7.50 – 7.44 (m, 2H, Ar), 7.44 – 7.39 (m, 1H, C(*H*)O_2), 6.04 (ddd, *J* = 17.3, 10.6, 5.5 Hz, 1H, C*H*=CH_2), 5.65 (dt, *J* = 17.3, 1.0 Hz, 1H, CH=CH_2), 5.46 (dt, *J* = 10.6, 1.0 Hz, 1H, CH=CH_2), 2.61 (hept, *J* = 7.0 Hz, 1H, C*H*$(CH_3)_2$), 1.20 (d, *J* = 7.0 Hz, 6H, CH(CH_3)$_2$)
^{13}C NMR (101 MHz, $CDCl_3$) δ 175.0 (6), 164.5 (14), 133.6 (2), 131.6 (9), 130.1 (5, 3), 129.4 (4), 128.6 (1, 11), 120.6 (10), 89.7 (12), 34.1 (16), 18.8 (18, 17)
$[\alpha]^{25}_{589}$ = +10.4 (c=1.0 in $CHCl_3$, 96% ee)
HRMS (ESI) *m/z* calcd. for $C_{14}H_{16}O_4Na$ $[M+Na]^+$: 271.0941, found: 271.0940
IR (ATR) ν (cm^{-1}, $CHCl_3$): 711, 957, 1084, 1265, 1749, 2977

(*S*)-1-(pivaloyloxy)allyl benzoate (2b)

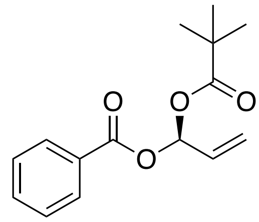

Reaction time: 1 hr. Purification: 0–5% Et_2O in Pentane. Yellow oil (97.3 mg, 93% yield, 95% ee).

The enantiomeric excess of 95% was determined by SFC [Chiralpak® ID; 1500 psi, 30°C; 0 to 5% MeOH in 5 mins; flow: 1 ml/min; λ= 225 nm; major enantiomer t_R = 3.21 mins, minor enantiomer t_R = 3.32 mins]

^{1}H NMR (400 MHz, $CDCl_3$) δ = 8.06 – 7.95 (m, 2H), 7.56 – 7.47 (m, 1H), 7.41 – 7.35 (m, 2H), 7.31 (dt, *J*=5.3, 1.1, 1H), 5.97 (ddd, *J*=17.3, 10.6, 5.3, 1H), 5.57 (dt, *J*=17.3, 1.1, 1H), 5.38 (dt, *J*=10.6, 1.0, 1H), 1.16 (s, 9H).
^{13}C NMR (101 MHz, $CDCl_3$) δ 176.4, 164.5, 133.6, 131.6, 130.0, 129.4, 128.6, 120.5, 89.7, 39.0, 27.0.
$[\alpha]^{25}_{589}$ = +7.2 (c=1.0 in $CHCl_3$, 95% ee)
HRMS (ESI) *m/z* calcd. for $C_{15}H_{18}O_4Na$ $[M+Na]^+$: 285.1098, found: 285.1099
IR (ATR) ν (cm^{-1}, $CHCl_3$): 711, 968, 1087, 1263, 1746, 2976

(*S*)-1-(benzoyloxy)allyl (3*S*,5*S*,7*S*)-adamantane-1-carboxylate (2c)

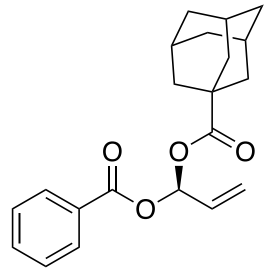

Reaction time: 1 hr. Purification: 0–5% Et_2O in petane. Colourless oil (119.5 mg, 88% yield, 93% ee)

The enantiomeric excess of 93% was determined by SFC [Chiralpak® IC; 1500 psi, 30°C; 1 to 30% MeOH in 5 mins; flow: 1.5 ml/min; λ= 230 nm; minor enantiomer t_R = 2.65 mins, major enantiomer t_R = 2.79 mins]

^{1}H NMR (400 MHz, $CDCl_3$) δ = 8.09 – 8.03 (m, 2H), 7.62 – 7.55 (m, 1H), 7.49 – 7.42 (m, 2H), 7.38 (dt, *J*=5.2, 1.1, 1H), 6.03 (ddd, *J*=17.3, 10.6, 5.2, 1H), 5.64 (dt, *J*=17.3, 1.1, 1H), 5.44 (dt, *J*=10.6, 1.1, 1H), 2.06 – 1.98 (m, 3H), 1.95 – 1.91 (m, 6H), 1.79 – 1.64 (m, 6H).
^{13}C NMR (101 MHz, $CDCl_3$) δ 175.4, 164.5, 133.6, 131.7, 130.1, 129.4, 128.6, 120.4, 89.5, 40.9, 38.6, 36.5, 27.9.
$[\alpha]^{25}_{589}$ = + 0.71 (c=1.04 in $CHCl_3$, 93% ee)
HRMS (ESI) *m/z* calcd. for $C_{21}H_{24}O_4Na$ $[M+Na]^+$: 363.1567, found: 363.1564
IR (ATR) ν (cm^{-1}, $CHCl_3$): 711, 952, 1053, 1265, 1744, 2907

(*R*)-1-(2-phenylacetoxy)allyl benzoate (2d)

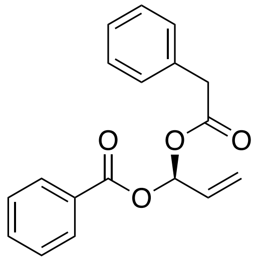

Reaction time: 2 hrs. Purification: 0–10% Et_2O in Pentane. Colourless oil (105.5 mg, 87% yield, 92% ee)

The enantiomeric excess of 92% was determined by SFC [Chiralpak® IB; 1500 psi, 30°C; 1 to 30% MeOH in 5 mins; flow: 1.5 ml/min; λ= 210 nm; major enantiomer t_R = 1.95 mins, minor enantiomer t_R = 2.09 mins]

^{1}H NMR (400 MHz, $CDCl_3$) δ 8.07 – 8.00 (m, 2H), 7.62 – 7.55 (m, 1H), 7.49 – 7.44 (m, 2H), 7.42 (dt, *J* = 5.5, 1.0 Hz, 1H), 7.36 – 7.25 (m, 5H), 6.02 (ddd, *J* = 17.3, 10.5, 5.4 Hz, 1H), 5.61 (dt, *J* = 17.3, 1.0 Hz, 1H), 5.44 (dt, *J* = 10.6, 0.9 Hz, 1H), 3.70 (s, 2H).
^{13}C NMR (101 MHz, $CDCl_3$) δ 169.5, 164.4, 133.7, 133.3, 131.3, 130.1, 129.4, 129.2, 128.7, 128.6, 127.4, 120.9, 90.0, 41.2.
$[\alpha]^{25}_{589}$ = +13.3 (c=1.0 in $CHCl_3$ 92% ee)
HRMS (ESI) *m/z* calcd. for $C_{18}H_{16}O_4Na$ $[M+Na]^+$: 319.0941, found: 319.0943
IR (ATR) ν (cm^{-1}, $CHCl_3$): 711, 965, 1088, 1267, 1733, 1752

(*R*)-1-acetoxyallyl benzoate (2e)

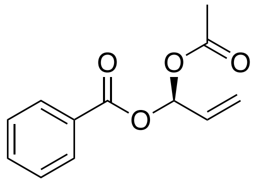

Reaction time: overnight. Purification: 0–10% Et_2O in Pentane. Colourless oil (42.4 mg, 48% yield, 74% ee)

The enantiomeric excess of 74% was determined by SFC [Chiralpak® ID; 1500 psi, 30°C; 1 to 30% MeOH in 5 mins; flow: 1.5 ml/min; λ= 210 nm; major enantiomer t_R = 1.36 mins, minor enantiomer t_R = 1.42 mins]

^{1}H NMR (400 MHz, $CDCl_3$) δ = 8.13 – 8.03 (m, 2H), 7.64 – 7.54 (m, 1H), 7.49 – 7.43 (m, 2H), 7.41 (dt, *J*=5.6, 1.1, 1H), 6.03 (ddd, *J*=17.3, 10.5, 5.5, 1H), 5.66 (dt, *J*=17.3, 1.0, 1H), 5.46 (dt, *J*=10.6, 1.0, 1H), 2.13 (s, 3H).
^{13}C NMR (101 MHz, $CDCl_3$) δ 168.8, 164.4, 133.7, 131.4, 130.1, 129.3, 128.6, 120.8, 89.7, 21.0.
$[\alpha]^{25}_{589}$ = +10.9 (c=1.0 in $CHCl_3$, 74% ee)
HRMS (ESI) *m/z* calcd. for $C_{12}H_{12}O_4Na$ $[M+Na]^+$: 243.0628, found: 243.0628
IR (ATR) ν (cm^{-1}, $CHCl_3$): 711, 956, 1218, 1269, 1734, 1757

(*R*)-1-(formyloxy)allyl benzoate (2f)

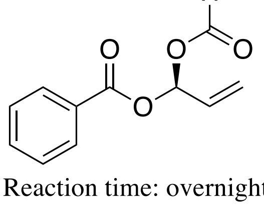

Reaction time: overnight. Purification: 0–5% Et_2O in Pentane. Colourless oil (29.8 mg, 29% yield, 67% ee)
Note: The reaction with formic acid also gave small amounts of the achiral dibenzoyloxy derivative of **2** and under some conditions **1a** may decompose to benzoic acid, which then undergoes competitive Rh-catalysed carboxylation to the remaining **1a**. We were not able to separate this by-product from **2f**.

The enantiomeric excess of 67% was determined by HPLC [Chiralpak® IC; hexane:^{i}PrOH 99:1; 1.3 ml.min^{-1}; λ= 210 nm; major enantiomer t_R = 13.61 mins, minor enantiomer t_R = 15.41 mins].

^{1}H NMR (400 MHz, $CDCl_3$) δ = 8.13 (d, *J*=0.8, 1H), 8.11 – 8.04 (m, 3H), 7.64 – 7.57 (m, 1H), 7.50 – 7.44 (m, 3H), 6.06 (ddd, *J*=17.2, 10.5, 5.6, 1H), 5.70 (dt, *J*=17.3, 0.9, 1H), 5.51 (dt, *J*=10.6, 0.9, 1H).
^{13}C NMR (101 MHz, $CDCl_3$) δ = 158.84, 133.93, 130.87, 130.13, 128.69, 128.61, 121.49, 89.30.
$[\alpha]^{25}_{589}$ = +13.7 (c=1.04 in $CHCl_3$, 67% ee)
HRMS (GCMS EI) *m/z* calcd. for $C_{11}H_{10}O_4P$ $[M]^+$: 206.0574, found: 206.0583
IR (ATR) ν (cm^{-1}, $CHCl_3$): 710, 953, 1065, 1085, 1264, 1740

(*R*)-1-(pent-4-enoyloxy)allyl benzoate (2g)

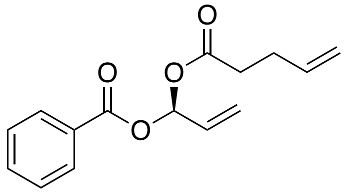

Reaction time: 2.25 hrs. Purification: 0–5% Et_2O in Pentane. Colourless oil (87.9 mg, 85% yield, 94% ee)

The enantiomeric excess of 94% was determined by SFC [Chiralpak® IC; 1500 psi, 30°C; 1 to 30% MeOH in 5 mins; flow: 1.5 ml/min; λ= 220 nm; minor enantiomer t_R = 1.65 mins, major enantiomer t_R = 1.69 mins]

^{1}H NMR (400 MHz, $CDCl_3$) δ 8.12 – 8.02 (m, 2H), 7.63 – 7.53 (m, 1H), 7.49 – 7.39 (m, 3H), 6.03 (ddd, *J* = 17.2, 10.5, 5.5 Hz, 1H), 5.81 (ddt, *J* = 16.5, 10.2, 6.2 Hz, 1H), 5.65 (dt, *J* = 17.3, 1.0 Hz, 1H), 5.46 (dt, *J* = 10.4, 1.0 Hz, 1H), 5.11 – 4.96 (m, 2H), 2.56 – 2.43 (m, 2H), 2.43 – 2.34 (m, 2H).
^{13}C NMR (101 MHz, $CDCl_3$) δ 170.8, 164.4, 136.3, 133.6, 131.4, 130.1, 130.0, 129.2, 128.5, 120.7, 120.7, 115.9, 89.6, 33.4, 28.6.
$[\alpha]^{25}_{589}$ = +8.9 (c=1.05 in $CHCl_3$, 94% ee)
HRMS (ESI) *m/z* calcd. for $C_{15}H_{16}O_4Na$ $[M+Na]^+$: 283.0941, found: 283.0942
IR (ATR) ν (cm^{-1}, $CHCl_3$): 711, 954, 1086, 1265, 1734, 2981

(*R*)-1-(pent-4-ynoyloxy)allyl benzoate (2h)

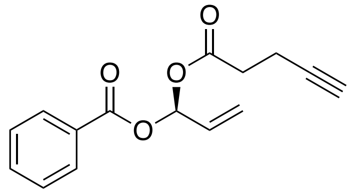

Reaction time: overnight. Purification: 0–5% Et_2O in Pentane. Slightly yellow oil (63.1 mg, 61% yield, 91% ee)

The enantiomeric excess of 91% was determined by SFC [Chiralpak® IF; 1500 psi, 30°C; 0 to 10% MeOH in 5 mins; flow: 1.0 ml/min; λ= 220 nm; minor enantiomer t_R = 3.44 mins, major enantiomer t_R = 3.50 mins]

^{1}H NMR (400 MHz, $CDCl_3$) δ 8.12 – 8.02 (m, 2H), 7.64 – 7.54 (m, 1H), 7.50 – 7.40 (m, 3H), 6.04 (ddd, *J* = 17.2, 10.5, 5.5 Hz, 1H), 5.67 (dt, *J* = 17.3, 1.0 Hz, 1H), 5.47 (dt, *J* = 10.5, 1.0 Hz, 1H), 2.70 – 2.58 (m, 2H), 2.62 – 2.46 (m, 2H), 1.96 (t, *J* = 2.6 Hz, 1H).
^{13}C NMR (101 MHz, $CDCl_3$) δ 169.6, 164.3, 133.6, 131.1, 130.0, 129.1, 128.5, 120.9, 120.9, 89.7, 82.0, 69.3, 33.2, 14.2.
$[\alpha]^{25}_{589}$ = +6.8 (c=1.01 in $CHCl_3$, 91% ee)
HRMS (ESI) *m/z* calcd. for $C_{15}H_{14}O_4Na$ $[M+Na]^+$: 281.0784, found: 281.0784
IR (ATR) ν (cm^{-1}, $CHCl_3$): 711, 955, 1089, 1265, 1732

(*R*)-1-(cinnamoyloxy)allyl benzoate (2i)

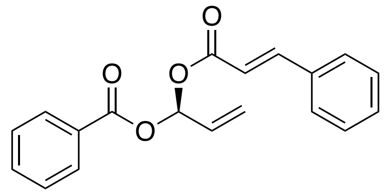

Reaction time: overnight. Purification: 0–10% Et_2O in Pentane. Colourless oil (84.7 mg, 65% yield, 92% ee)

The enantiomeric excess of 92% was determined by SFC [Chiralpak® ID; 1500 psi, 30°C; 1 to 20% MeOH in 5 mins; 1.5 ml/min; λ= 215 nm; minor enantiomer t_R = 3.37 mins, major enantiomer t_R = 3.47 mins]

^{1}H NMR (400 MHz, $CDCl_3$) δ 8.13 – 8.06 (m, 2H), 7.78 (d, *J* = 16.0 Hz, 1H), 7.62 – 7.55 (m, 2H), 7.55 – 7.50 (m, 2H), 7.49 – 7.43 (m, 2H), 7.41 – 7.37 (m, 3H), 6.47 (d, *J* = 16.0 Hz, 1H), 6.12 (ddd, *J* = 17.3, 10.5, 5.4 Hz, 1H), 5.72 (dt, *J* = 17.3, 1.0 Hz, 1H), 5.50 (dt, *J* = 10.6, 1.0 Hz, 1H).
^{13}C NMR (101 MHz, $CDCl_3$) δ 164.7, 164.5, 146.7, 134.2, 133.7, 131.6, 130.8, 130.1, 129.3, 129.1, 128.6, 128.4, 120.8, 117.0, 89.9.
$[\alpha]^{25}_{589}$ = –15.5 (c=1.0 in $CHCl_3$, 92% ee)
HRMS (ESI) *m/z* calcd. for $C_{19}H_{16}O_4Na$ $[M+Na]^+$: 331.0941, found: 331. 0938
IR (ATR) ν (cm^{-1}, $CHCl_3$): 709, 962, 1264, 1635, 1734

(1*R*)-1-((2-(4-isobutylphenyl)propanoyl)oxy)allyl benzoate (2j)

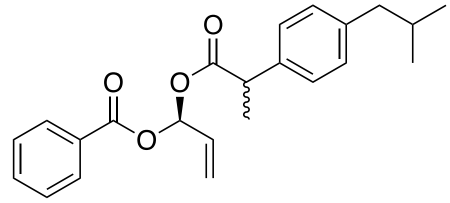

Reaction time: 1 hr. Purification: 0–10% Et_2O in Pentane. Colourless oil (132 mg, 90% yield, 1:1 d.r. 94% ee)

The enantiomeric excess of 94% was determined by SFC [Chiralpak® IG; 1500 psi; 30°C; 95:5 CO_2:MeOH; flow: 1.5 ml/min; λ= 220 nm; **D1** – major enantiomer t_R = 1.81 mins, minor enantiomer t_R = 2.62 mins; **D2** – major enantiomer t_R = 1.93 mins, minor enantiomer t_R = 2.20 mins]

^{1}H NMR (400 MHz, $CDCl_3$) δ 8.04 (dd, *J* = 8.4, 1.3 Hz, 2H, **D1**), 7.92 (dd, *J* = 8.4, 1.3 Hz, 2H, **D2**), 7.61 – 7.53 (m, 2H, **D1** and **D2**), 7.48 – 7.37 (m, 6H, **D1** and **D2**), 7.23 – 7.16 (m, 4H, **D1** and **D2**), 7.08 (d, *J* = 14.2 Hz, 2H, **D1**), 7.06 (d, *J* = 14.3 Hz, 2H, **D2**), 6.00 (ddd, *J* = 16.3, 10.0, 5.0 Hz, 1H, **D2**), 5.92 (ddd, 1H, **D1**), 5.60 (dt, *J* = 17.2, 1.0 Hz, 1H, **D2**), 5.44 (dt, *J* = 3.2, 0.9 Hz, 1H, **D1**), 5.41 (dt, *J* = 3.4, 1.0 Hz, 1H, **D1**), 5.33 (dt, *J* = 10.6, 1.0 Hz, 1H, **D2**), 3.80 – 3.75 (m, 1H, **D2**), 3.75 – 3.71 (m, 1H, **D1**), 2.44 (d, *J* = 7.2 Hz, 2H, **D1**), 2.42 (d, *J* = 7.2 Hz, 2H, **D2**), 1.85 (t, *J* = 6.7 Hz, 1H, **D2**), 1.80 (dd, *J* = 13.0, 6.3 Hz, 1H, **D1**), 1.52 (d, *J* = 5.0 Hz, 2H, **D2**), 1.50 (d, *J* = 5.1 Hz, 3H, **D1**), 0.89 (d, *J* = 6.6 Hz, 6H, **D1**), 0.87 (d, *J* = 6.6 Hz, 6H, **D2**).
^{13}C NMR (101 MHz, $CDCl_3$) δ 172.6, 164.5 (**D2**), 164.3 (**D1**), 140.8 (**D1**), 140.7 (**D2**), 137.2 (**D1**), 137.1 (**D2**), 133.7 (**D1**), 133.5 (**D2**), 131.4 (**D1**), 131.3 (**D2**), 130.1 (**D1**), 130.0 (**D2**), 129.5 (**D1**), 129.4 (**D2**), 129.3 (**D1**), 129.3 (**D2**), 128.6 (**D1**), 128.5 (**D2**), 127.4, 120.8 (**D2**), 120.5 (**D1**), 89.8 (**D2**), 89.6 (**D1**), 45.2, 45.1, 30.3x, 22.5, 18.5 (**D1**), 18.4 (**D2**).
$[\alpha]^{25}_{589}$ = –17.2 (c=1.04 in $CHCl_3$, 94% ee)
HRMS (ESI) *m/z* calcd. for $C_{23}H_{26}O_4Na$ $[M+Na]^+$: 389.1723, found: 389.1723
IR (ATR) ν (cm^{-1}, $CHCl_3$): 711, 951, 1063, 1265, 1749, 2360, 2980

(S)-1-(benzoyloxy)allyl 2,4,6-trimethylbenzoate (2k)

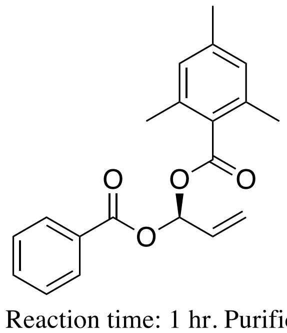

Reaction time: 1 hr. Purification: 0–5% Et_2O in Pentane. Colourless oil (117.4 mg, 91% yield, 91% ee)

The enantiomeric excess of 91% was determined by analytical chiral SFC [Chiralpak® IC; 1500 psi, 30°C; 1 to 30% MeOH in 5 mins; flow: 1.5 ml/min; λ= 225 nm; minor enantiomer t_R = 2.36 mins, Major enantiomer t_R = 2.52 mins]

^{1}H NMR (400 MHz, $CDCl_3$) δ = 8.12 – 8.04 (m, 2H), 7.69 – 7.62 (m, 1H), 7.65 – 7.55 (m, 1H), 7.51 – 7.41 (m, 2H), 6.85 (s, 2H), 6.13 (ddd, *J*=17.3, 10.5, 5.8, 1H), 5.76 (dt, *J*=17.3, 1.0, 1H), 5.52 (dt, *J*=10.5, 0.9, 1H), 2.32 (s, 6H), 2.27 (s, 3H).
^{13}C NMR (101 MHz, $CDCl_3$) δ 167.8, 164.4, 139.9, 135.7, 133.7, 131.4, 130.1, 129.8, 129.3, 128.6, 121.2, 90.2, 21.3, 19.9.
$[\alpha]^{25}_{589}$ = –39.8 (c=1.03 in $CHCl_3$, 91% ee)
HRMS (ESI) *m/z* calcd. for $C_{20}H_{20}O_4Na$ $[M+Na]^+$: 347.1254, found: 347.1253
IR (ATR) ν (cm^{-1}, $CHCl_3$): 710, 956, 1054, 1242, 1269, 1741

(S)-1-(benzoyloxy)allyl 3,4-dimethoxybenzoate (2l)

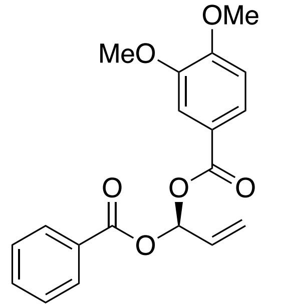

Reaction time: 2 hrs. Purification: 0–40% Et_2O in Pentane. Yellow oil (119.8 mg, 88% yield, 93% ee)

The enantiomeric excess of 93% was determined by SFC [Chiralpak® IG; 1500 psi, 30°C; 1 to 30% MeOH in 5 mins; flow: 1.5 ml/min; λ= 220 nm; minor enantiomer t_R = 4.76 mins, major enantiomer t_R = 4.98 mins]

^{1}H NMR (400 MHz, Chloroform-*d*) δ 8.13 – 8.05 (m, 2H), 7.74 (dd, *J* = 8.5, 2.0 Hz, 1H), 7.66 (dt, *J* = 5.4, 1.1 Hz, 1H), 7.63 – 7.53 (m, 2H), 7.49 – 7.40 (m, 2H), 6.88 (d, *J* = 8.5 Hz, 1H), 6.17 (ddd, *J* = 17.3, 10.6, 5.4 Hz, 1H), 5.74 (dt, *J* = 17.3, 1.1 Hz, 1H), 5.51 (dt, *J* = 10.5, 1.0 Hz, 1H), 3.93 (d, *J* = 6.4 Hz, 6H).
^{13}C NMR (101 MHz, Chloroform-*d*) δ 164.4, 164.1, 153.5, 148.7, 133.5, 131.6, 130.0, 129.2, 128.5, 124.2, 121.5, 120.7, 112.2, 110.2, 90.0, 56.1, 56.0, 15.3.
$[\alpha]^{25}_{589}$ = –7.9 (c=1.01 in $CHCl_3$, 93% ee)
HRMS (ESI) *m/z* calcd. for $C_{19}H_{18}O_6Na$ $[M+Na]^+$: 365.0996, found: 365.0994
IR (ATR) ν (cm^{-1}, $CHCl_3$): 711, 952, 1024, 1267, 1732

(*S*)-1-(benzoyloxy)allyl 2,3,5,6-tetrafluorobenzoate (2m)

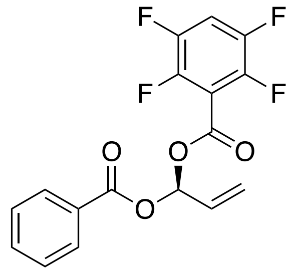

Reaction time: overnight. Purification: 0–5% Et_2O in Pentane. Colourless oil (77.9 mg, 55% yield, 57% ee)

The enantiomeric excess of 57% was determined by SFC [Chiralpak® IE, 1500 psi, 30°C; 1 to 30% MeOH in 5 mins; flow: 1.5 ml/min; λ= 220 nm; minor enantiomer t_R = 1.78 mins, major enanatiomer t_R = 1.84 mins]

^{1}H NMR (400 MHz, $CDCl_3$) δ = 8.16 – 8.05 (m, 2H), 7.66 – 7.62 (m, 1H), 7.62 – 7.58 (m, 1H), 7.55 – 7.39 (m, 3H), 7.22 (tt, *J*=9.4, 7.2, 1H), 6.14 (ddd, *J*=17.3, 10.5, 5.6, 1H), 5.80 (dt, *J*=17.2, 0.9, 1H), 5.57 (dt, *J*=10.6, 0.9, 1H).
^{13}C NMR (101 MHz, $CDCl_3$) δ 164.3, 157.6, 147.6 – 147.0 (m), 146.4 – 146.0 (m), 145.0 – 144.6 (m), 143.8 – 143.4 (m), 134.0, 130.5, 130.2, 128.8, 128.7, 122.0, 109.5 (t, *J* = 22.4 Hz), 90.9.
^{19}F NMR (377 MHz, $CDCl_3$) δ = -136.33 – - 137.21 (2F, m), -138.34 – -138.82 (2F, m).
$[\alpha]^{25}_{589}$ = –2.1 (c=1.01 in $CHCl_3$, 57% ee)
HRMS (ESI) *m/z* calcd. for $C_{17}H_{10}O_4F_4Na$ $[M+Na]^+$: 377.0407, found: 377.0408
IR (ATR) ν (cm^{-1}, $CHCl_3$): 711, 953, 1262, 1298, 1503, 1749

(*S*)-1-(benzoyloxy)allyl 5-bromo-2-chlorobenzoate (2n)

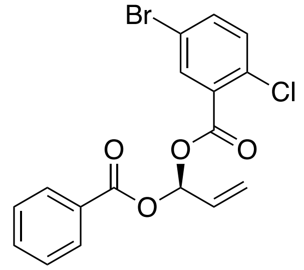

Reaction time: 5 hrs. Purification: 0–5% Et_2O in Pentane. Yellow oil (129.1 mg, 82% yield, 81% ee)

The enantiomeric excess of 81% was determined by SFC [Chiralpak® IG; 1500 psi, 30°C; 1 to 30% MeOH in 5 mins; flow: 1.5 ml/min; λ= 210 nm; major enantiomer t_R = 4.48 mins, minor enantiomer t_R = 4.78 mins]

^{1}H NMR (400 MHz, $CDCl_3$) δ 8.12 – 8.05 (m, 2H), 7.77 (d, *J* = 8.4 Hz, 1H), 7.64 (d, *J* = 1.9 Hz, 1H), 7.63 – 7.57 (m, 2H), 7.50 – 7.42 (m, 3H), 6.15 (ddd, *J* = 17.3, 10.6, 5.6 Hz, 1H), 5.77 (dt, *J* = 17.3, 1.0 Hz, 1H), 5.54 (dt, *J* = 10.6, 0.9 Hz, 1H).
^{13}C NMR (101 MHz, $CDCl_3$) δ 164.4, 162.6, 135.7, 134.2, 133.8, 133.1, 131.0, 130.2, 129.1, 128.7, 127.7, 127.3, 121.5, 90.7.
$[\alpha]^{25}_{589}$ = –0.6 (c=1.02 in $CHCl_3$, 81% ee)
HRMS (ESI) *m/z* calcd. for $C_{17}H_{12}O_4BrClNa$ $[M+Na]^+$: 416.9500, found: 416.9501
IR (ATR) ν (cm^{-1}, $CHCl_3$): 711, 952, 1083, 1234, 1580, 1742

(*S*)-1-(benzoyloxy)allyl 2-chlorobenzoate (2o)

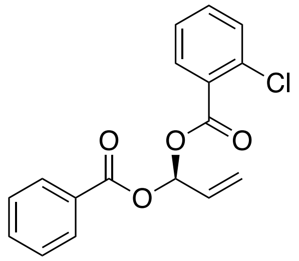

Reaction time: 1.5 hrs. Purification: 0–10% Et_2O in Pentane. Yellow oil (110.9 mg, 88% yield, 89% ee)

The enantiomeric excess of 89% was determined by SFC [Chiralpak® IG; 1500 psi; 30°C; 1 to 30% MeOH in 5 mins; flow: 1.5 ml/min; λ= 220 nm; major enantiomer t_R = 3.83 mins, minor enantiomer t_R = 4.12 mins]

^{1}H NMR (400 MHz, $CDCl_3$) δ 8.14 – 8.07 (m, 2H), 7.93 – 7.86 (m, 1H), 7.64 (dt, *J* = 5.5, 1.0 Hz, 1H), 7.62 – 7.57 (m, 1H), 7.49 – 7.41 (m, 4H), 7.32 (ddd, *J* = 7.8, 6.6, 2.1 Hz, 1H), 6.16 (ddd, *J* = 17.3, 10.5, 5.6 Hz, 1H), 5.78 (dt, *J* = 17.3, 1.0 Hz, 1H), 5.54 (dt, *J* = 10.6, 1.0 Hz, 1H).
^{13}C NMR (101 MHz, $CDCl_3$) δ 164.5, 163.4, 134.5, 133.8, 133.3, 131.9, 131.4, 131.2, 130.2, 129.2, 129.0, 128.6, 126.8, 121.3, 90.6.
$[\alpha]^{25}_{589}$ = +3.0 (c=1.02 in $CHCl_3$, 89% ee)
HRMS (ESI) *m/z* calcd. for $C_{17}H_{13}O_4ClNa$ $[M+Na]^+$: 339.0395, found: 339.0398
IR (ATR) ν (cm^{-1}, $CHCl_3$): 711, 951, 1085, 1940, 1742, 2981

(*S*)-1-(benzoyloxy)allyl 2-bromobenzoate (2p)

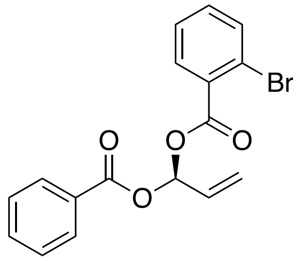

Reaction time: 2.5 hrs. Purification: 0–10% Et_2O in Pentane. Colourless oil (130.5 mg, 91% yield, 73% ee)

The enantiomeric excess of 73% was determined by SFC [Chiralpak® IG; 1500 psi; 30°C; 1 to 30% MeOH in 5 mins; flow: 1.5 ml/min; λ= 225 nm; major enantiomer t_R = 4.27 mins, minor enantiomer t_R = 4.66 mins]

^{1}H NMR (400 MHz, $CDCl_3$) δ 8.15 – 8.06 (m, 2H), 7.90 – 7.83 (m, 1H), 7.71 – 7.63 (m, 2H), 7.60 (ddt, *J* = 8.0, 6.9, 1.3 Hz, 1H), 7.50 – 7.43 (m, 2H), 7.40 – 7.31 (m, 2H), 6.17 (ddd, *J* = 17.3, 10.5, 5.6 Hz, 1H), 5.78 (dt, *J* = 17.3, 1.0 Hz, 1H), 5.54 (dt, *J* = 10.6, 0.9 Hz, 1H).
^{13}C NMR (101 MHz, $CDCl_3$) δ 164.3, 163.7, 134.6, 133.6, 133.1, 131.8, 131.1, 131.0, 130.0, 129.1, 128.5, 127.2, 122.2, 121.2.
$[\alpha]^{25}_{589}$ = +6.6 (c=1.0 in $CHCl_3$, 73% ee)
HRMS (APCI) *m/z* calcd. for $C_{17}H_{13}O_4BrNa$ $[M+Na]^+$: 382.9889, found: 382.9886
IR (ATR) ν (cm^{-1}, $CHCl_3$): 710, 951, 1081, 1240, 1744

(*S*)-1-(benzoyloxy)allyl 2-nitrobenzoate (2q)

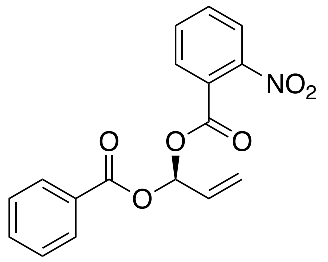

Reaction time: overnight. Purification: 0–25% Et_2O in Pentane. Colourless oil (105.7 mg, 80% yield, 66% ee)

The enantiomeric excess of 66% was determined by SFC [Chiralpak® IG; 1500 psi; 30°C; 1 to 30% MeOH in 5 mins; flow: 1.5 ml/min; λ= 220 nm; major enantiomer t_R = 3.96 mins, minor enantiomer t_R = 4.62 mins]

^{1}H NMR (400 MHz, $CDCl_3$) δ 8.16 – 8.07 (m, 2H), 7.99 – 7.91 (m, 1H), 7.81 – 7.74 (m, 1H), 7.72 – 7.55 (m, 4H), 7.47 (ddt, *J* = 7.9, 6.6, 1.2 Hz, 2H), 6.11 (ddd, *J* = 17.3, 10.6, 5.6 Hz, 1H), 5.77 (dt, *J* = 17.2, 0.9 Hz, 1H), 5.55 (dt, *J* = 10.5, 0.9 Hz, 1H).
^{13}C NMR (101 MHz, $CDCl_3$) δ 164.4, 163.5, 148.1, 133.9, 133.2, 132.2, 130.5, 130.2, 130.2, 129.0, 128.7, 127.0, 124.2, 121.7, 90.9.
$[\alpha]^{25}_{589}$ = –24.5 (c=1.07 in $CHCl_3$, 66% ee)
HRMS (method) *m/z* calcd. for $C_{17}H_{13}O_6NNa$ $[M+Na]^+$: 350.0635, found: 350.0639
IR (ATR) ν (cm^{-1}, $CHCl_3$): 712, 954, 1059, 1246, 1536, 1747

(*S*)-1-(benzoyloxy)allyl 4-chlorobenzoate (2r)

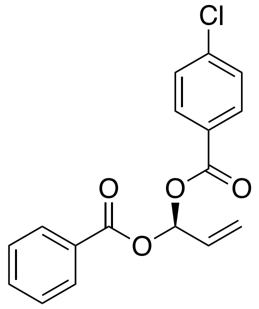

Reaction time: 5.5 hrs. Purification: 0–5% Et_2O in Pentane. Colourless oil (111.3 mg, 88% yield, 91% ee)

The enantiomeric excess of 91% was determined by SFC [Chiralpak® IC; 1500 psi, 30°C; 1 to 30% MeOH in 5 mins; flow: 1.5 ml/min; λ= 235 nm; minor enantiomer t_R = 2.49 mins, Major enantiomer t_R = 2.62 mins]

^{1}H NMR (400 MHz, $CDCl_3$) δ 8.13 – 8.07 (m, 2H), 8.06 – 7.98 (m, 2H), 7.65 (dt, *J* = 5.5, 1.0 Hz, 1H), 7.64 – 7.56 (m, 1H), 7.48 – 7.40 (m, 4H), 6.16 (ddd, *J* = 17.3, 10.6, 5.5 Hz, 1H), 5.75 (dt, *J* = 17.3, 1.0 Hz, 1H), 5.53 (dt, *J* = 10.6, 0.9 Hz, 1H).
^{13}C NMR (101 MHz, $CDCl_3$) δ 164.4, 163.7, 140.2, 133.8, 131.5, 131.4, 130.1, 129.2, 129.0, 128.6, 127.7, 121.2, 90.3.
$[\alpha]^{25}_{589}$ = –2.7 (c=1.03 in $CHCl_3$, 91% ee)
HRMS (ESI) *m/z* calcd. for $C_{17}H_{13}O_4ClNa$ $[M+Na]^+$: 339.0395, found: 339.0395
IR (ATR) ν (cm^{-1}, $CHCl_3$): 710, 950, 1062, 1247, 1738, 2981

(*S*)-1-(benzoyloxy)allyl 4-bromobenzoate (2s)

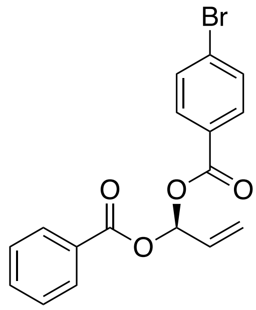

Reaction time: overnight. Purification: 0–5% Et_2O in Pentane. Colourless oil (94.1 mg, 65% yield, 91% ee)

The enantiomeric excess of 91% was determined by SFC [Chiralpak® IC; 1500 psi, 30°C; 1 to 30% MeOH in 5 mins; flow: 1.5 ml/min; λ= 235 nm; minor enantiomer t_R = 2.74 mins, major enantiomer t_R = 2.89 mins]

^{1}H NMR (400 MHz, $CDCl_3$) δ 8.12 – 8.06 (m, 2H), 7.97 – 7.91 (m, 2H), 7.65 (dt, *J* = 5.5, 1.0 Hz, 1H), 7.62 – 7.56 (m, 3H), 7.49 – 7.42 (m, 2H), 6.16 (ddd, *J* = 17.3, 10.6, 5.5 Hz, 1H), 5.75 (dt, *J* = 17.3, 1.0 Hz, 1H), 5.53 (dt, *J* = 10.6, 1.0 Hz, 1H).
^{13}C NMR (101 MHz, $CDCl_3$) δ 164.4, 163.8, 133.8, 132.0, 131.6, 131.3, 130.1, 129.1, 128.9, 128.6, 128.2, 121.2, 90.3.
$[\alpha]^{25}_{589}$ = –4.3 (c=1.0 in $CHCl_3$, 91% ee)
HRMS (ESI) *m/z* calcd. for $C_{17}H_{13}O_4BrNa$ $[M+Na]^+$: 382.9889, found: 382.9893
IR (ATR) ν (cm^{-1}, $CHCl_3$): 712, 954, 1059, 1246, 1536, 1747

(*S*)-1-(benzoyloxy)allyl 4-fluorobenzoate (2t)

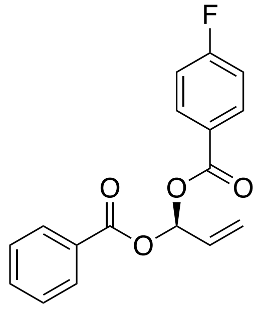

Reaction time: overnight. Purification: 0–5% Et_2O in Pentane. Slightly yellow oil (83.3 mg, 67% yield, 88% ee)

The enantiomeric excess of 88% was determined by SFC [Chiralpak® IC; 1500 psi, 30°C; 1 to 30% MeOH in 5 mins; flow: 1.5 ml/min; λ= 220 nm; minor enantiomer t_R = 2.07 mins, major enantiomer t_R = 2.16 mins]

^{1}H NMR (400 MHz, $CDCl_3$) δ 8.16 – 8.05 (m, 4H), 7.65 (dt, *J* = 5.5, 1.1 Hz, 1H), 7.59 (ddt, *J* = 7.9, 7.0, 1.3 Hz, 1H), 7.50 – 7.41 (m, 2H), 7.17 – 7.07 (m, 2H), 6.16 (ddd, *J* = 17.3, 10.6, 5.5 Hz, 1H), 5.75 (dt, *J* = 17.3, 1.0 Hz, 1H), 5.53 (dt, *J* = 10.6, 0.9 Hz, 1H).
^{13}C NMR (101 MHz, $CDCl_3$) δ 167.5, 165.0, 164.5, 163.5, 133.8, 132.8, 132.7, 131.4, 130.1, 129.2, 128.6, 121.1, 115.9, 115.7, 90.3, 48.3, 15.4.
^{19}F NMR (377 MHz, $CDCl_3$) δ -104.44.
$[\alpha]^{25}_{589}$ = +3.03 (c=1.0 in $CHCl_3$, 88% ee)
HRMS (GCMS EI) *m/z* calcd. for $C_{17}H_{13}O_4F$ $[M]^+$: 300.0792, found: 300.0795
IR (ATR) ν (cm^{-1}, $CHCl_3$): 711, 950, 1061, 1246, 1604, 1739

(*S*)-1-(benzoyloxy)allyl 4-methoxybenzoate (2u)

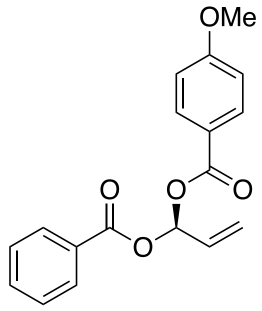

Reaction Time: overnight. Purification: 0–10% Et_2O in Pentane. Colourless oil (42.6 mg, 34% yield, 83% ee)

The enantiomeric excess of 83% was determined by HPLC [Chiralpak® IB; 99:1 Hexane:IPA; 1 ml/min; λ= 250 nm; major enantiomer t_R = 12.89 mins, minor enantiomer t_R = 14.27 mins]

^{1}H NMR (400 MHz, Chloroform-*d*) δ 8.13 – 8.07 (m, 2H), 8.06 – 8.02 (m, 2H), 7.66 (dt, *J* = 5.4, 1.1 Hz, 1H), 7.63 – 7.53 (m, 1H), 7.49 – 7.40 (m, 2H), 6.96 – 6.88 (m, 2H), 6.16 (ddd, *J* = 17.3, 10.5, 5.4 Hz, 1H), 5.74 (dt, *J* = 17.3, 1.1 Hz, 1H), 5.50 (dt, *J* = 10.6, 1.0 Hz, 1H), 3.86 (s, 3H).
^{13}C NMR (101 MHz, $CDCl_3$) δ 164.5, 164.2, 164.0, 133.6, 132.3, 131.8, 130.1, 129.4, 128.6, 121.6, 120.7, 113.9, 90.0, 55.6.
$[\alpha]^{20}_{589}$ = –6.7 (c=1.0 in $CHCl_3$, 83% ee)
HRMS (GCMS with ammonia as reagent gas) *m/z* calcd. for $C_{18}H_{20}NO_5$ $[M+NH_4]^+$: 330.1336, found: 330.1318
IR (ATR) ν (cm^{-1}, $CHCl_3$): 711, 950, 1061, 1249, 1605, 1733, 2980

(*S*)-1-(benzoyloxy)allyl 4-(trifluoromethyl)benzoate (2v)

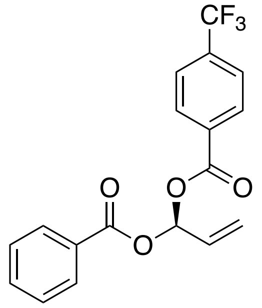

Reaction Time: overnight. Purification: 0–5% Et_2O in Pentane. Colourless oil (98.1 mg, 70% yield, 80% ee)

The enantiomeric excess of 80% was determined by SFC [Chiralpak® IG, 1500 psi, 30°C; 1 to 30% MeOH in 5 mins; flow: 1.5 ml/min; λ= 220 nm; minor enantiomer t_R = 1.91 mins, major enantiomers t_R = 2.02 mins]

^{1}H NMR (400 MHz, $CDCl_3$) δ = 8.20 (dp, *J*=7.8, 0.9, 2H), 8.11 – 8.07 (m, 2H), 7.75 – 7.69 (m, 2H), 7.69 – 7.67 (m, 1H), 7.63 – 7.57 (m, 1H), 7.50 – 7.43 (m, 2H), 6.17 (ddd, *J*=17.3, 10.6, 5.6, 1H), 5.77 (dt, *J*=17.3, 1.0, 1H), 5.55 (dt, *J*=10.6, 0.9, 1H).
^{13}C NMR (101 MHz, $CDCl_3$) δ 164.4, 163.3, 135.1 (q, *J* = 32.7 Hz), 133.9, 132.6, 131.2, 130.5, 130.2, 129.1, 128.7, 125.6 (q, *J* = 3.7 Hz), 121.3, 90.5.
^{19}F NMR (377 MHz, $CDCl_3$) δ = -63.19.
$[\alpha]^{25}_{589}$ = +6.6 (c=1.01 in $CHCl_3$, 80% ee)
HRMS (ESI) *m/z* calcd. for $C_{18}H_{13}O_4F_3Na$ $[M+Na]^+$: 373.0658, found: 373.0658
IR (ATR) ν (cm^{-1}, $CHCl_3$): 709, 951, 1066, 1325, 1742

(*S*)-1-(benzoyloxy)allyl 4-nitrobenzoate (2w)

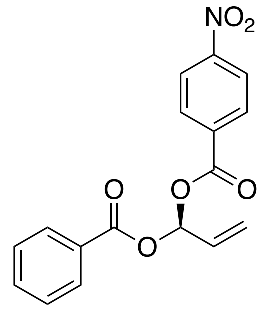

Reaction Time: overnight. Purification: 0–10% Et_2O in Pentane. Yellow oil (86.2 mg, 66% yield, 66% ee)

The enantiomeric excess of 66% was determined by SFC [Chiralpak® IG; 1500 psi, 30°C; 1 to 30% MeOH in 5 mins; flow: 1.5 ml/min; λ= 220 nm; minor enantiomer t_R = 4.21 mins, major enantiomer t_R = 4.44 mins]

^{1}H NMR (400 MHz, $CDCl_3$) δ = 8.32 – 8.22 (m, 4H), 8.11 – 8.06 (m, 2H), 7.67 (dt, *J*=5.7, 1.0, 1H), 7.64 – 7.58 (m, 1H), 7.50 – 7.44 (m, 2H), 6.18 (ddd, *J*=17.3, 10.6, 5.7, 1H), 5.78 (dt, *J*=17.3, 0.9, 1H), 5.57 (dt, *J*=10.5, 0.9, 1H).
^{13}C NMR (101 MHz, $CDCl_3$) δ 164.4, 162.7, 134.7, 134.0, 131.3, 131.0, 130.2, 128.9, 128.7, 123.8, 121.7, 90.8.
$[\alpha]^{25}_{589}$ = +3.2 (c=1.0 in $CHCl_3$, 66% ee)
HRMS (ESI) *m/z* calcd. for $C_{17}H_{13}O_6NNa$ $[M+Na]^+$: 350.0635, found: 350.0636
IR (ATR) ν (cm^{-1}, $CHCl_3$): 711, 953, 1064, 1246, 1527, 1741

(*S*)-1-(benzoyloxy)allyl 4-hydroxybenzoate (2xa)

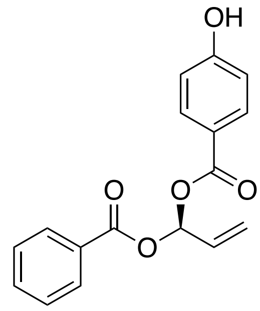

Reaction time: overnight. Purification: 0–30% Et_2O in Pentane. Colourless oil (89.8 mg, 75% yield, 91% ee)

The enantiomeric excess of 91% was determined by SFC [Chiralpak® IG; 1500 psi, 30°C; 1 to 30% MeOH in 5 mins; flow: 1.5 ml/min; λ= 210 nm; major enantiomer t_R = 3.91 mins, minor enantiomer t_R = 3.98 mins]

^{1}H NMR (400 MHz, $CDCl_3$) δ 8.14 – 8.04 (m, 1H), 7.98 (d, *J* = 8.7 Hz, 1H), 7.65 (dt, *J* = 5.4, 1.0 Hz, 1H), 7.58 (ddt, *J* = 7.9, 7.0, 1.3 Hz, 1H), 7.44 (ddt, *J* = 7.8, 6.6, 1.1 Hz, 2H), 6.91 – 6.83 (m, 2H), 6.16 (ddd, *J* = 17.3, 10.6, 5.4 Hz, 1H), 5.97 (s, 1H), 5.74 (dt, *J* = 17.3, 1.0 Hz, 1H), 5.51 (dt, *J* = 10.6, 1.0 Hz, 1H).
^{13}C NMR (101 MHz, $CDCl_3$) δ 164.8, 164.4, 160.7, 133.8, 132.6, 131.6, 130.2, 129.3, 128.6, 120.9, 115.5, 90.2.
$[\alpha]^{25}_{589}$ = –11.7 (c=1.0 in $CHCl_3$, 91% ee)
HRMS (ESI) *m/z* calcd. for $C_{17}H_{14}O_5Na$ $[M+Na]^+$: 321.0733, found: 321.0735
IR (ATR) ν (cm^{-1}, $CHCl_3$): 711, 950, 1063, 1255, 1607, 1732, 3380

(*S*)-1-(benzoyloxy)allyl 2-hydroxybenzoate (2xb)

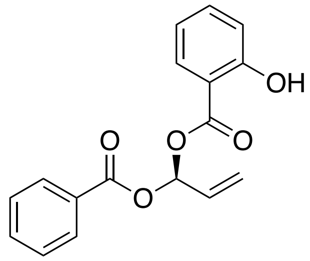

Reaction time: overnight. Purification: 0–10% Et_2O in Pentane. Colourless oil (54.1 mg, 45% yield, 37% ee)

The enantiomeric excess of 37% was determined by SFC [Chiralpak® IG; 1500 psi, 30°C; 1 to 30% MeOH in 5 mins; flow: 1.5 ml/min; λ= 220 nm; minor enantiomer t_R = 2.98 mins, major enantiomer t_R = 3.49 mins]

^{1}H NMR (400 MHz, $CDCl_3$) δ 10.47 (s, 1H), 8.17 – 8.06 (m, 2H), 7.89 (dd, *J* = 8.0, 1.7 Hz, 1H), 7.69 (dt, *J* = 5.5, 1.0 Hz, 1H), 7.65 – 7.56 (m, 1H), 7.47 (dtd, *J* = 8.0, 6.8, 6.3, 1.2 Hz, 3H), 6.99 (dd, *J* = 8.5, 1.1 Hz, 1H), 6.89 (ddd, *J* = 8.3, 7.2, 1.1 Hz, 1H), 6.17 (ddd, *J* = 17.3, 10.6, 5.5 Hz, 1H), 5.77 (dt, *J* = 17.3, 0.9 Hz, 1H), 5.56 (dt, *J* = 10.6, 0.9 Hz, 1H).
^{13}C NMR (101 MHz, $CDCl_3$) δ 168.1, 164.3, 162.1, 136.4, 133.8, 130.9, 130.1 (d, *J* = 7.8 Hz), 128.8, 128.6, 121.4, 119.3, 117.8, 111.5, 89.9.
$[\alpha]^{25}_{589}$ = +10.2 (c=1.05 in $CHCl_3$, 37% ee)
HRMS (ESI) *m/z* calcd. for $C_{17}H_{14}O_5Na$ $[M+Na]^+$: 321.0733, found: 321.0734
IR (ATR) ν (cm^{-1}, $CHCl_3$): 710, 953, 1062, 1247, 1737, 3230

(*S*)-1-((*S*)-2-acetoxy-2-phenylacetoxy)allyl benzoate (2yb)

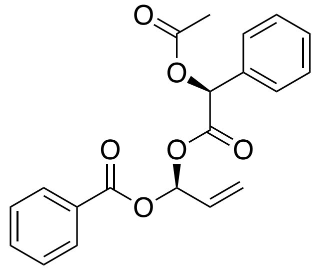

Reaction time: overnight. Purification: 0–15% Et_2O in Pentane. Yellow oil (60.7 mg, 43% yield, 78% ee)

The enantiomeric excess of 78% was determined by SFC [Chiralpak® IE; 1500 psi, 30°C; 1 to 30% MeOH in 5 mins; flow: 1.5 ml/min; λ= 220 nm; major enantiomer t_R = 2.83 mins, minor enantiomer t_R = 3.11 mins]

^{1}H NMR (400 MHz, $CDCl_3$) δ 8.1 – 8.0 (m, 2H), 7.7 – 7.6 (m, 1H), 7.5 – 7.3 (m, 8H), 6.0 (s, 1H), 5.9 (ddd, *J* = 17.3, 10.6, 5.2 Hz, 1H), 5.5 (dd, *J* = 17.3, 1.1 Hz, 1H), 5.4 (dd, *J* = 10.6, 1.0 Hz, 1H), 2.2 (s, 3H).
^{13}C NMR (101 MHz, $CDCl_3$) δ 170.1, 166.8, 164.1, 133.6, 133.3, 130.6, 130.1, 129.5, 128.9, 128.9, 128.5, 127.8, 120.9, 90.1, 74.2, 20.7.
$[\alpha]^{25}_{589}$ = +70.2 (c=1.0 in $CHCl_3$, 78% ee)
HRMS (ESI) *m/z* calcd. for $C_{20}H_{18}O_6Na$ $[M+Na]^+$: 377.0996, found: 377.0989
IR (ATR) ν (cm^{-1}, $CHCl_3$): 711, 957, 1053, 1228, 1740, 2360

2-((*S*)-1-(benzoyloxy)allyl) 1-(*tert*-butyl) (*R*)-pyrrolidine-1,2-dicarboxylate (3b)

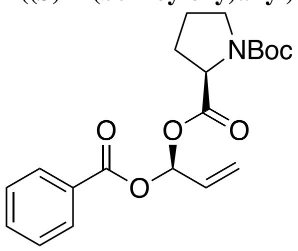

Reaction time: overnight. Purification: 0–30% Et_2O in Pentane. Yellow oil (103.6 mg, 69% yield, 90% ee). The product is obtained as ~2:1 ratio of rotamers, as confirmed by NOESY NMR (see fig. S27), NMR assignments below are for the major rotamer.

The enantiomeric excess of 90% was determined by SFC [Chiralpak® IE; 1500 psi, 30°C; 1 to 30% MeOH in 5 mins; flow: 1.5 ml/min; λ= 220 nm; major enantiomer t_R = 3.39 mins, minor enantiomer t_R = 3.57 mins]

^{1}H NMR Major Rotamer (400 MHz, $CDCl_3$) δ 8.10 – 8.02 (m, 2H), 7.64 – 7.55 (m, 1H), 7.49 – 7.40 (m, 3H), 6.03 (ddd, *J* = 17.2, 10.6, 5.7 Hz, 1H), 5.67 (d, *J* = 17.2 Hz, 1H), 5.47 (d, *J* = 10.5 Hz, 1H), 4.29 (dd, *J* = 8.7, 4.0 Hz, 1H), 3.57 – 3.40 (m, 2H), 2.31 – 2.16 (m, 1H), 2.07 – 1.97 (m, 1H), 1.97 – 1.79 (m, 2H), 1.43 (s, 3H), 1.37 (s, 6H).
^{13}C NMR Major Rotamer (101 MHz, $CDCl_3$) δ 171.0, 164.3, 153.8, 133.8, 131.2, 130.0, 129.1, 128.6, 121.2, 90.1, 80.3, 59.2, 46.4, 31.0, 28.5, 28.4, 23.6.
$[\alpha]^{25}_{589}$ = –53.6 (c=1.0 in $CHCl_3$, 90% ee)
HRMS (ESI) *m/z* calcd. for $C_{20}H_{25}NO_6Na$ $[M+Na]^+$: 398.1574, found: 398.1573
IR (ATR) ν (cm^{-1}, $CHCl_3$): 712, 956, 1160, 1395, 1700, 2360, 2980

(*S*)-1-(benzoyloxy)allyl 4-(dimethylamino)benzoate (3d)

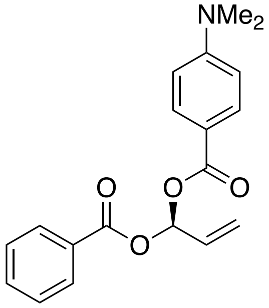

Reaction time: overnight. Purification: 0–30% Et_2O in Pentane. Yellow oil (76.7 mg, 59% yield, 94% ee)

The enantiomeric excess of 94% was determined by SFC [Chiralpak® IB; 1500 psi, 30°C; 1 to 30% MeOH in 5 mins; flow: 1.5 ml/min; λ= 220 nm; major enantiomer t_R = 3.38 mins, minor enantiomer t_R = 3.47 mins]

^{1}H NMR (400 MHz, $CDCl_3$) δ 8.17 – 8.07 (m, 2H), 8.02 – 7.93 (m, 2H), 7.68 (dt, *J* = 5.3, 1.1 Hz, 1H), 7.64 – 7.54 (m, 1H), 7.51 – 7.41 (m, 2H), 6.70 – 6.62 (m, 2H), 6.19 (ddd, *J* = 17.3, 10.6, 5.3 Hz, 1H), 5.75 (dt, *J* = 17.3, 1.1 Hz, 1H), 5.51 (dt, *J* = 10.6, 1.0 Hz, 1H), 3.07 (s, 6H).
^{13}C NMR (101 MHz, $CDCl_3$) δ 164.6, 164.4, 153.7, 133.4, 132.0, 131.8, 130.0, 129.5, 128.4, 120.2, 115.5, 110.7, 89.6, 40.1.
$[\alpha]^{25}_{589}$ = –45.0 (c=1.0 in $CHCl_3$, 94% ee)
HRMS (ESI) *m/z* calcd. for $C_{19}H_{19}NO_4Na$ $[M+Na]^+$: 348.1206, found: 348.1204
IR (ATR) ν (cm^{-1}, $CHCl_3$): 711, 946, 1060, 1259, 1605, 1731, 2360, 2981

(*S*)-1-(benzoyloxy)allyl 2-chloronicotinate (3f)

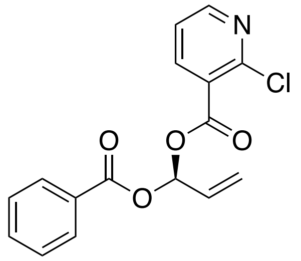

Reaction time: overnight. Purification: 0–30% Et_2O in Pentane. Colourless oil (59.3 mg, 47% yield, 67% ee)

The enantiomeric excess of 67% was determined by SFC [Chiralpak® ID; 1500 psi, 30°C; 1 to 30% MeOH in 5 mins; flow: 1.5 ml/min; λ= 220 nm; major enantiomer t_R = 3.10 mins, minor enantiomer t_R = 3.35 mins]

^{1}H NMR (400 MHz, $CDCl_3$) δ = 8.53 (dd, *J*=4.8, 2.0, 1H), 8.21 (dd, *J*=7.7, 2.0, 1H), 8.14 – 8.05 (m, 2H), 7.63 (dt, *J*=5.6, 1.0, 1H), 7.61 – 7.57 (m, 1H), 7.51 – 7.41 (m, 2H), 7.33 (dd, *J*=7.7, 4.8, 1H), 6.16 (ddd, *J*=17.3, 10.6, 5.7, 1H), 5.78 (dt, *J*=17.3, 0.9, 1H), 5.56 (dt, *J*=10.6, 0.9, 1H).
^{13}C NMR (101 MHz, $CDCl_3$) δ = 164.4, 162.3, 152.5, 150.5, 140.8, 133.9, 130.8, 130.1, 128.9, 128.7, 126.0, 122.2, 121.7, 90.9.
$[\alpha]^{25}_{589}$ = +4.1 (c=1.04 in $CHCl_3$, 67% ee)
HRMS (ESI) *m/z* calcd. for $C_{16}H_{12}O_4NClNa$ $[M+Na]^+$: 340.0347, found: 340.0348
IR (ATR) ν (cm^{-1}, $CHCl_3$): 711, 953, 1043, 1261, 1406, 1738

(*S*)-1-(benzoyloxy)allyl 2-chloroisonicotinate (3g)

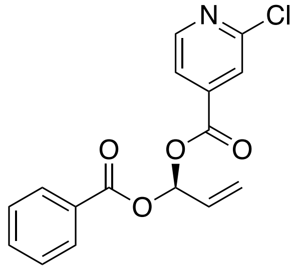

Reaction time: overnight. Purification: 0–30% Et_2O in Pentane. Orange oil (65.2 mg, 51% yield, 69% ee)

The enantiomeric excess of 69% was determined by SFC [Chiralpak® ID; 1500 psi, 30°C; 1 to 30% MeOH in 5 mins; flow: 1.5 ml/min; λ= 220 nm; major enantiomer t_R = 2.60 mins, minor enantiomer t_R = 2.71 mins]

^{1}H NMR (400 MHz, $CDCl_3$) δ = 8.55 (dd, *J*=5.1, 0.8, 1H), 8.13 – 8.04 (m, 2H), 7.93 – 7.87 (m, 1H), 7.80 (dd, *J*=5.1, 1.4, 1H), 7.67 – 7.62 (m, 1H), 7.61 – 7.58 (m, 1H), 7.51 – 7.42 (m, 2H), 6.15 (ddd, *J*=17.3, 10.6, 5.7, 1H), 5.77 (dt, *J*=17.2, 0.9, 1H), 5.57 (dt, *J*=10.6, 0.9, 1H).
^{13}C NMR (101 MHz, $CDCl_3$) δ 164.3, 161.9, 152.6, 150.7, 139.5, 134.0, 130.7, 130.1, 128.7, 128.7, 124.3, 121.9, 90.9.
$[\alpha]^{25}_{589}$ = +6.5 (c=1.0 in $CHCl_3$, 69% ee)
HRMS (ESI) *m/z* calcd. for $C_{16}H_{12}O_4NClNa$ $[M+Na]^+$: 340.0347, found: 340.0348
IR (ATR) ν (cm^{-1}, $CHCl_3$): 711, 954, 1081, 1245, 1370, 1743

(*E*)-3-bromoprop-1-en-1-yl 2,4,6-trimethylbenzoate (4a)

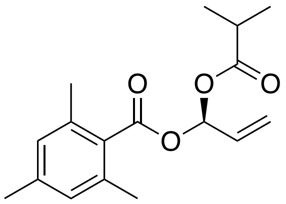

Reaction time: 50 minutes. Purification: 0–10% Et_2O in Pentane. Colourless oil (107.0 mg, 92% yield, 95% ee)

The enantiomeric excess of 95% was determined by SFC [Chiralpak® IC; 1500 psi, 30°C; 1 to 30% MeOH in 5 mins; flow: 1.5 ml/min; λ= 215 nm; minor enantiomer t_R = 1.61 mins, major enantiomer t_R = 1.67 mins]

^{1}H NMR (400 MHz, $CDCl_3$) δ 7.40 (dt, *J* = 5.7, 1.0 Hz, 1H), 6.88 – 6.82 (m, 2H), 6.00 (ddd, *J* = 17.3, 10.5, 5.8 Hz, 1H), 5.66 (dt, *J* = 17.3, 1.0 Hz, 1H), 5.45 (dt, *J* = 10.6, 1.0 Hz, 1H), 2.61 (hept, *J* = 7.0 Hz, 1H), 2.30 (s, 6H), 2.28 (s, 3H), 1.21 (d, *J* = 7.0 Hz, 6H).
^{13}C NMR (101 MHz, $CDCl_3$) δ 174.8, 167.8, 139.9, 135.6, 131.5, 129.9, 128.6, 120.9, 89.6, 34.1, 21.3, 18.9, 18.8.
$[\alpha]^{25}_{589}$ = +24.1 (c=1.02 in $CHCl_3$, 95% ee)
HRMS (ESI) *m/z* calcd. for $C_{17}H_{22}O_4Na$ $[M+Na]^+$: 313.1410, found: 313.1410
IR (ATR) ν (cm^{-1}, $CHCl_3$): 958, 1061, 1258, 1753, 2360

(E)-3-bromoprop-1-en-1-yl cinnamate (4b)

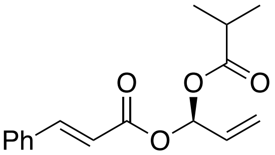

Reaction time: 30 minutes. Purification: 0–5% Et_2O in Pentane. Slightly yellow oil (90.2 mg, 82% yield, 93% ee)

The enantiomeric excess of 93% was determined by SFC [Chiralpak® IA; 1500 psi, 30°C; 1 to 30% MeOH in 5 mins; flow: 1.5 ml/min; λ= 275 nm; major enantiomer t_R = 1.66 mins, minor enantiomer t_R = 1.76 mins]

^{1}H NMR (400 MHz, $CDCl_3$) δ 7.75 (d, *J* = 16.0 Hz, 1H), 7.56 – 7.49 (m, 2H), 7.43 – 7.35 (m, 3H), 7.29 (dt, *J* = 5.4, 1.0 Hz, 1H), 6.44 (d, *J* = 16.0 Hz, 1H), 5.99 (ddd, *J* = 17.3, 10.6, 5.4 Hz, 1H), 5.62 (dt, *J* = 17.3, 1.0 Hz, 1H), 5.44 (dt, *J* = 10.6, 1.0 Hz, 1H), 2.61 (hept, *J* = 7.0 Hz, 1H), 1.20 (d, *J* = 7.0 Hz, 6H).
^{13}C NMR (101 MHz, $CDCl_3$) δ 174.9, 164.7, 146.5, 134.3, 131.6, 130.8, 129.1, 128.4, 120.5, 117.1, 89.3, 34.1, 18.8 (d, *J* = 2.3 Hz).
$[\alpha]^{25}_{589}$ = +20.0 (c=1.02 in $CHCl_3$, 93% ee)
HRMS (ESI) *m/z* calcd. for $C_{16}H_{18}O_4Na$ $[M+Na]^+$: 297.1097, found: 297.1098
IR (ATR) ν (cm^{-1}, $CHCl_3$): 768, 963, 1127, 1636, 1747, 2360

(*R*)-1-acetoxyallyl cinnamate (4c)

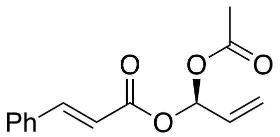

Reaction time: 1.5 hrs. Purification: 0–10% Et_2O in Pentane. Yellow oil (82.7 mg, 84% yield, 92% ee)

The enantiomeric excess of 92% was determined by SFC [Chiralpak® IA; 1500 psi, 30°C; 1 to 30% MeOH in 5 mins; flow: 1.5 ml/min; λ= 275 nm; major enantiomer t_R = 1.66 mins, minor enantiomer t_R = 1.77 mins]

^{1}H NMR (400 MHz, $CDCl_3$) δ 7.76 (d, *J* = 16.0 Hz, 1H), 7.58 – 7.49 (m, 2H), 7.45 – 7.36 (m, 3H), 7.30 (dt, *J* = 5.5, 1.0 Hz, 1H), 6.44 (d, *J* = 16.0 Hz, 1H), 5.98 (ddd, *J* = 17.2, 10.5, 5.5 Hz, 1H), 5.62 (dt, *J* = 17.3, 1.0 Hz, 1H), 5.44 (dt, *J* = 10.5, 1.0 Hz, 1H), 2.13 (s, 3H).
^{13}C NMR (101 MHz, $CDCl_3$) δ 168.8, 164.7, 146.7, 134.2, 131.5, 130.8, 129.1, 128.4, 120.7, 117.0, 89.4, 21.0.
$[\alpha]^{25}_{589}$ = +23.4 (c=1.0 in $CHCl_3$, 92% ee)
HRMS (ESI) *m/z* calcd. for $C_{14}H_{14}O_4Na$ $[M+Na]^+$: 269.0784, found: 269.0785
IR (ATR) ν (cm^{-1}, $CHCl_3$): 768, 965, 1150, 1222, 1636, 1756

(*R*)-5-oxo-2,5-dihydrofuran-2-yl acetate (5a)

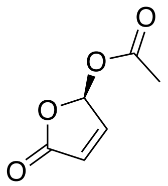

(*R*)-1-acetoxyallyl cinnamate (**4c**, 57 mg, 0.23 mmol, 1.0 eq.) was dissolved in CH_2Cl_2 (46 mL) and the resulting solution, degassed by bubbling through argon for 30 minutes. Grubbs I catalyst (19 mg, 0.023 mmol, 0.1 eq.) was added, the flask fitted with a reflux condenser and the reaction stirred at 50 °C for 24 hrs. SiO_2 (40 mg) was added and the solvent was then carefully evaporated. The resulting solid was directly loaded onto a chromatographic column and eluted with 0–50% Et_2O/pentane to afford the product as a brown oil (19.8 mg, 61% yield, 93% ee). Absolute stereochemistry was assigned by comparison with the literature.[2]

The enantiomeric excess of 93% was determined by SFC [Chiralpak® IG; 1500 psi, 30°C; 1 to 30% MeOH in 5 mins; flow: 1.5 ml/min; λ= 215 nm; major enantiomer t_R = 1.99 mins, minor enantiomer t_R = 2.32 mins
^{1}H NMR (400 MHz, $CDCl_3$) δ 7.32 (dd, *J* = 5.6, 1.3 Hz, 1H), 7.00 (t, *J* = 1.3 Hz, 1H), 6.32 (dd, *J* = 5.7, 1.2 Hz, 1H), 2.17 (s, 3H).
^{13}C NMR (101 MHz, $CDCl_3$) δ 169.7, 169.0, 149.8, 125.4, 93.9, 20.8.
$[\alpha]^{25}_{589}$ = +21.4 (c=1.02 in $CHCl_3$, 93% ee)
HRMS (ESI) *m/z* calcd. for $C_6H_7O_4$ $[M+H]^+$: 143.0339, found: 143.0340
IR (ATR) ν (cm^{-1}, $CHCl_3$): 820, 880, 1023, 1086, 1212, 1791

(*R*)-5-oxo-2,5-dihydrofuran-2-yl benzoate (5b)

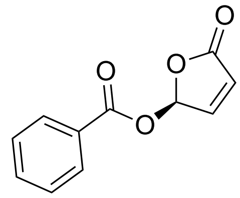

(*R*)-1-(cinnamoyloxy)allyl benzoate (**2i**, 80 mg, 0.26 mmol, 1.0 eq.) was dissolved in CH_2Cl_2 (52 mL) and the resulting solution, degassed by bubbling through argon for 30 minutes. Grubbs I catalyst (21.3 mg, 0.026 mmol, 0.1 eq.) was added, the flask fitted with a reflux condenser and the reaction stirred at 50 °C for 24 hrs. SiO_2 (40 mg) was added and the solvent was then carefully evaporated. The resulting solid was directly loaded onto a chromatographic column and eluted with 0–40% Et_2O/pentane to afford the product as an off-white solid (40.4 mg, 76% yield, 92% ee).

The enantiomeric excess of 92% was determined by SFC [Chiralpak® IC; 1500 psi, 30°C; 1 to 30% MeOH in 5 mins; flow: 1.5 ml/min; λ= 235 nm; major enantiomer t_R = 3.04 mins, minor enantiomer t_R = 3.48 mins]

^{1}H NMR (400 MHz, $CDCl_3$) δ 8.09 – 8.01 (m, 2H), 7.67 – 7.58 (m, 1H), 7.52 – 7.41 (m, 3H), 7.25 (t, *J* = 1.3 Hz, 1H), 6.39 (dd, *J* = 5.7, 1.2 Hz, 1H).
^{13}C NMR (101 MHz, $CDCl_3$) δ 169.6, 164.5, 149.8, 134.2, 130.1, 128.7, 128.2, 125.4, 94.5.
$[\alpha]^{25}_{589}$ = +110.2 (c=1.0 in $CHCl_3$, 92% ee)
HRMS (ESI) *m/z* calcd. for $C_{11}H_8O_4Na$ $[M+Na]^+$: 227.0315, found: 227.0316
IR (ATR) ν (cm^{-1}, $CHCl_3$): 718, 891, 1084, 1252, 1736, 1795

(*R*)-5-oxo-2,5-dihydrofuran-2-yl benzoate (5c)

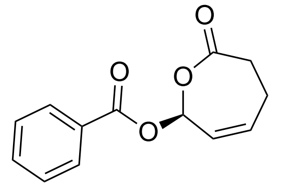

(*R*)-1-(pent-4-enoyloxy)allyl benzoate (**2g**, 47.5 mg, 0.183 mmol, 1.0 eq.) was dissolved in CH_2Cl_2 (36 mL) and the resulting solution, degassed by bubbling through argon for 45 minutes. Grubbs II catalyst (15.5 mg, 0.018 mmol, 0.1 eq.) was added, the flask fitted with a reflux condenser and the reaction stirred at 50 °C for 24 hrs. SiO_2 (40 mg) was added and the solvent was then carefully evaporated. The resulting solid was directly loaded onto a chromatographic column and eluted with 0–40% Et_2O/pentane to afford the product as an off-white solid (43% yield, >99% ee). Trace impurities were removed by triturating in hexane. No change in ee was observed before and after triturating.

The enantiomeric excess of >99% was determined by SFC [Chiralpak® IB; 1500 psi, 30°C; 97:3 CO_2:MeOH; flow: 1.5 ml/min; λ= 225 nm; major enantiomer t_R = 6.96 mins, minor enantiomer not observed]

^{1}H NMR (400 MHz, $CDCl_3$) δ 8.11 – 8.06 (m, 1H), 7.62 – 7.56 (m, 1H), 7.49 – 7.44 (m, 1H), 7.35 (d, *J* = 7.6 Hz, 0H), 6.07 (dt, *J* = 15.7, 6.2 Hz, 1H), 5.83 (dd, *J* = 15.5, 7.6 Hz, 1H), 2.68 – 2.55 (m, 1H), 2.50 – 2.43 (m, 2H).
^{13}C NMR (101 MHz, $CDCl_3$) δ 169.5, 164.4, 135.7, 133.8, 130.1, 129.3, 128.6, 125.7, 90.9, 33.0, 27.8.
$[\alpha]^{25}_{589}$ = –4.0 (c=1.0 in $CHCl_3$, >99% ee)
HRMS (ESI) *m/z* calcd. for $C_{13}H_{12}O_4Na$ $[M+Na]^+$: 255.0628, found: 255.0628
IR (ATR) ν (cm^{-1}, $CHCl_3$): 711, 957, 1065, 1269, 1747
m.p.: 183–186°C

References

(1) Lombardo, M.; Morganti, S.; Trombini, C. *J. Org. Chem.* **2003**, *68* (3), 997.

(2) Brinksma, J.; van der Deen, H.; van Oeveren, A.; Feringa, B. L. *J. Chem. Soc. Perkin Trans. 1* **1998**, *0* (24), 4159.

Supplementary Figure 1: ^{1}H, ^{13}C NMR and SFC trace for **2a**

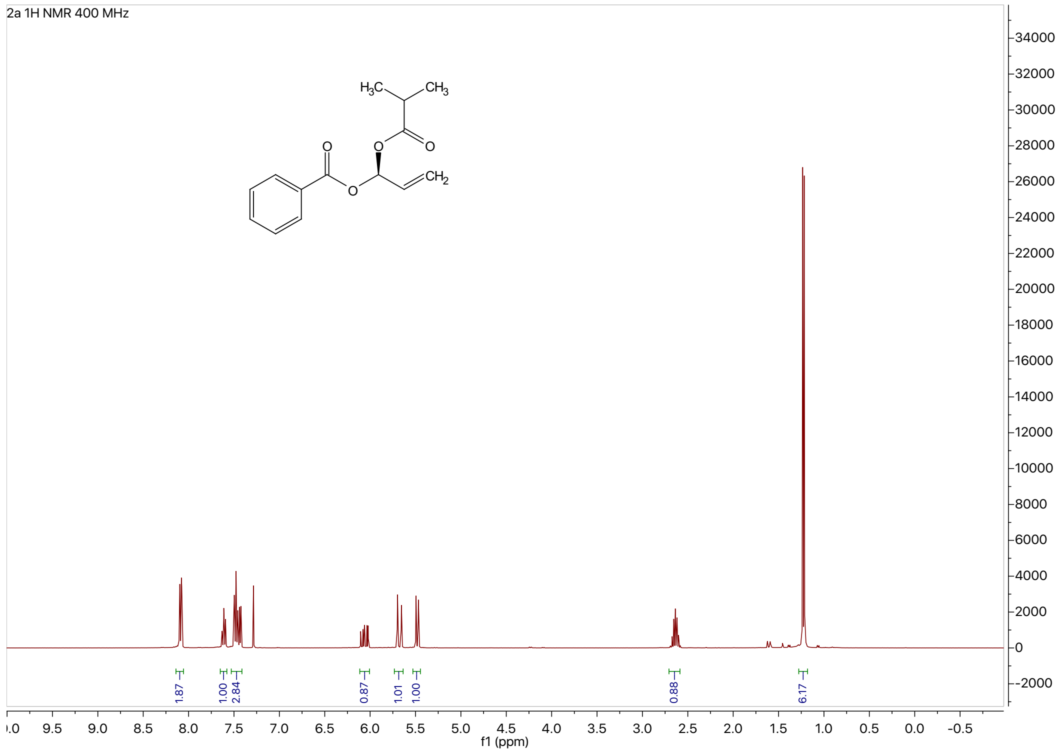

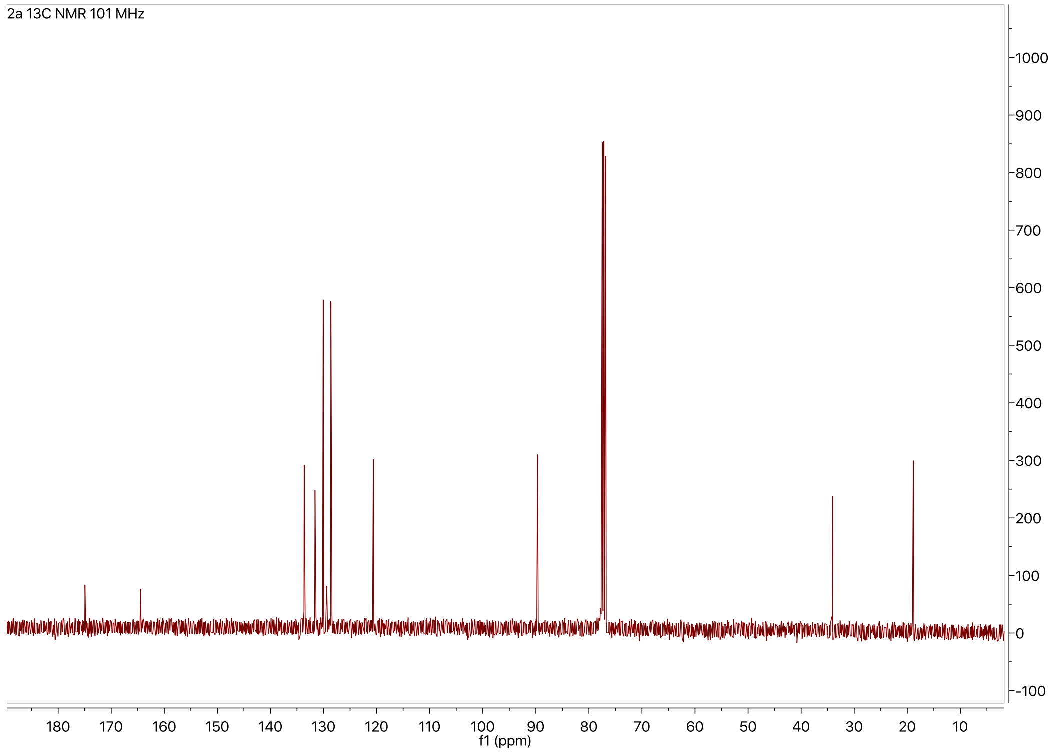

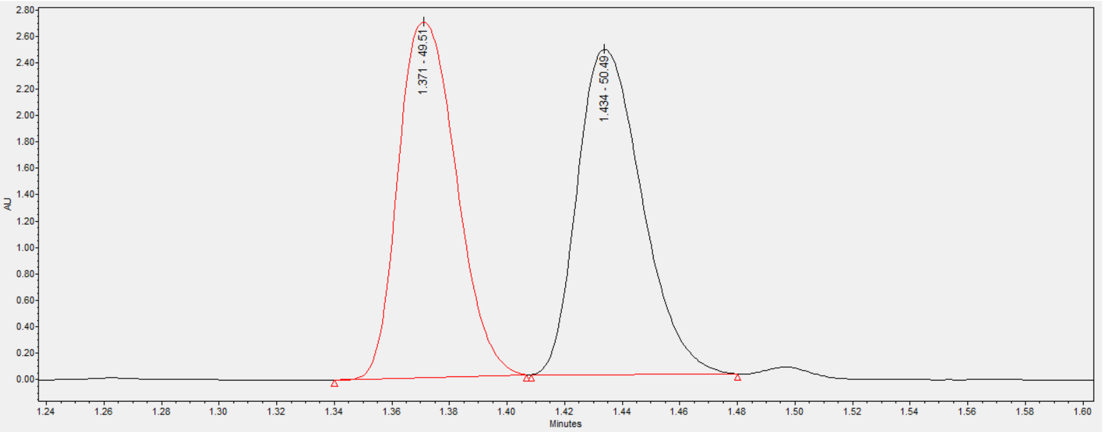
1.371 - 49.51
1.434 - 50.49
AU
Minutes

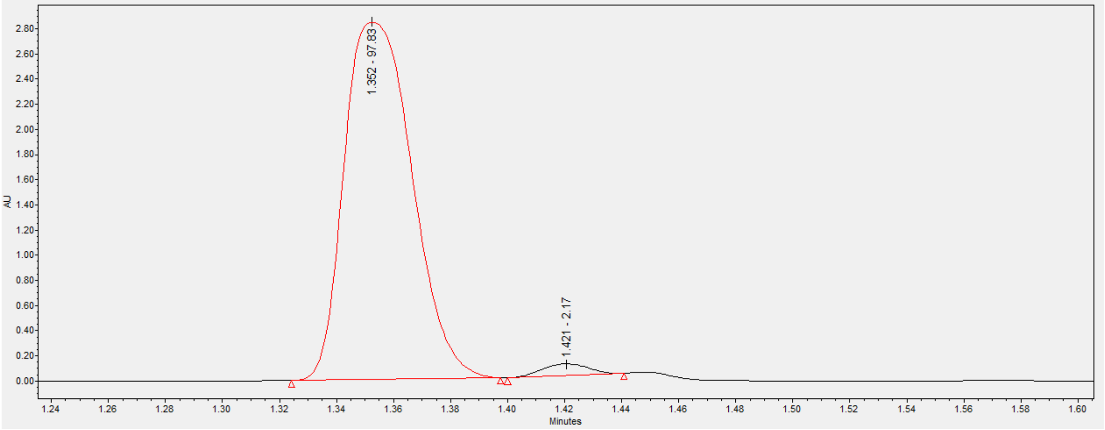
1.352 - 97.83
1.421 - 2.17
AU
Minutes

Supplementary Figure 2: ^{1}H, ^{13}C NMR and SFC trace for **2b**

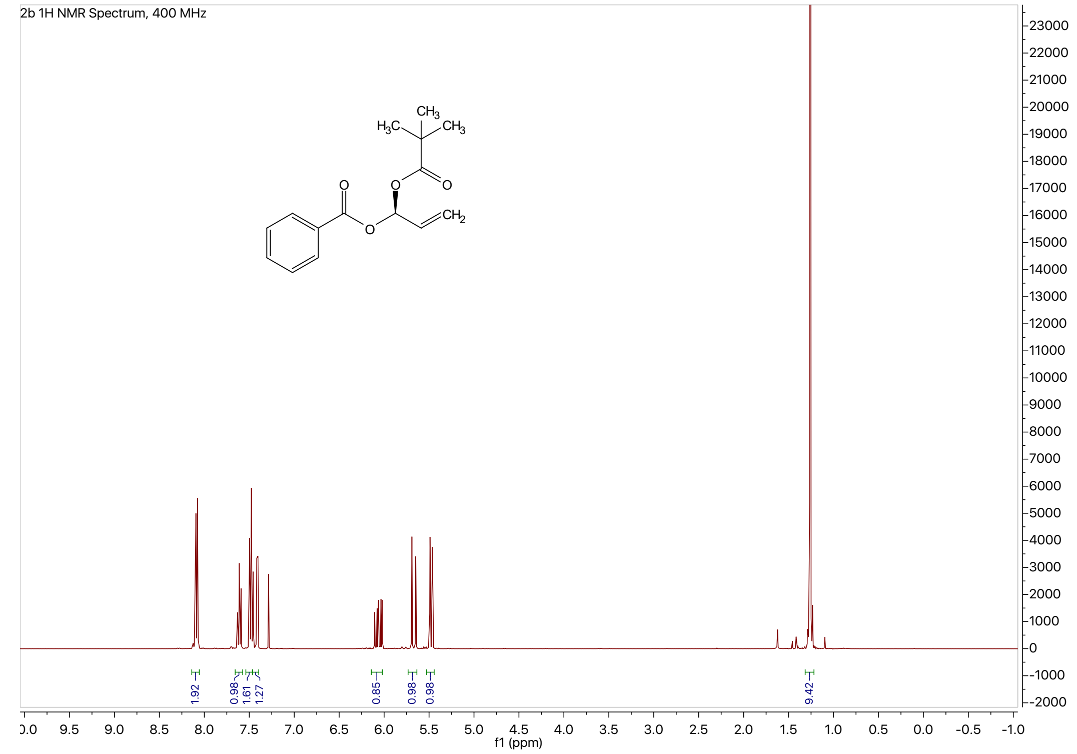

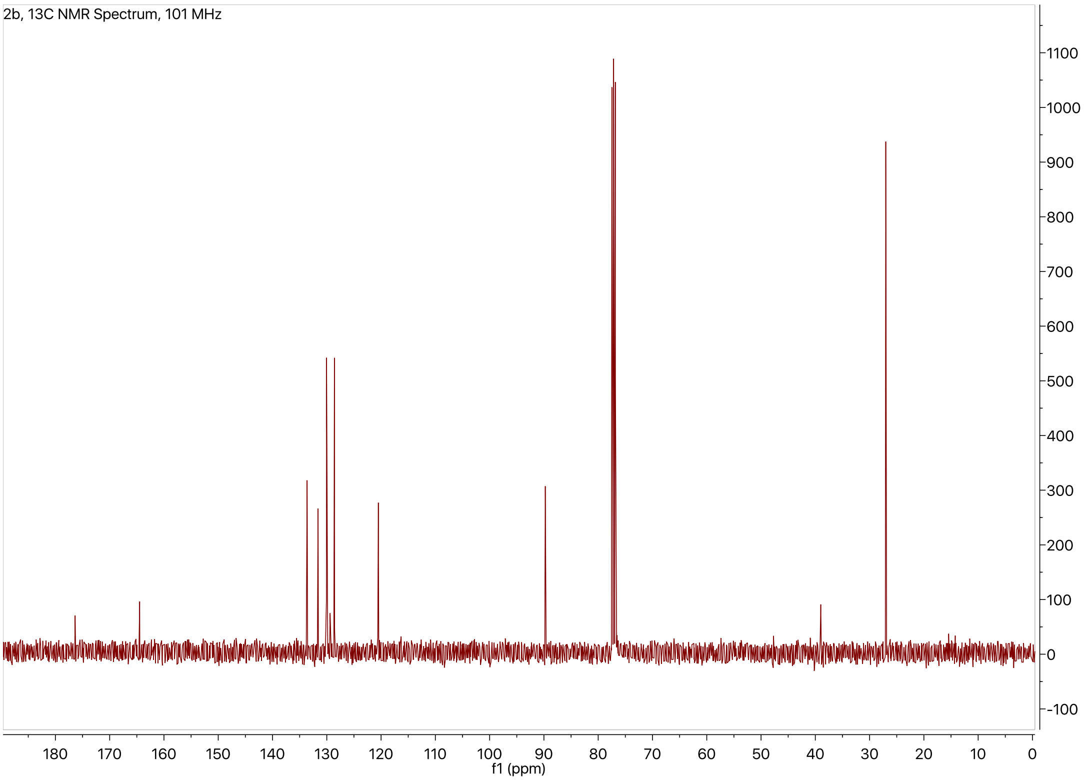

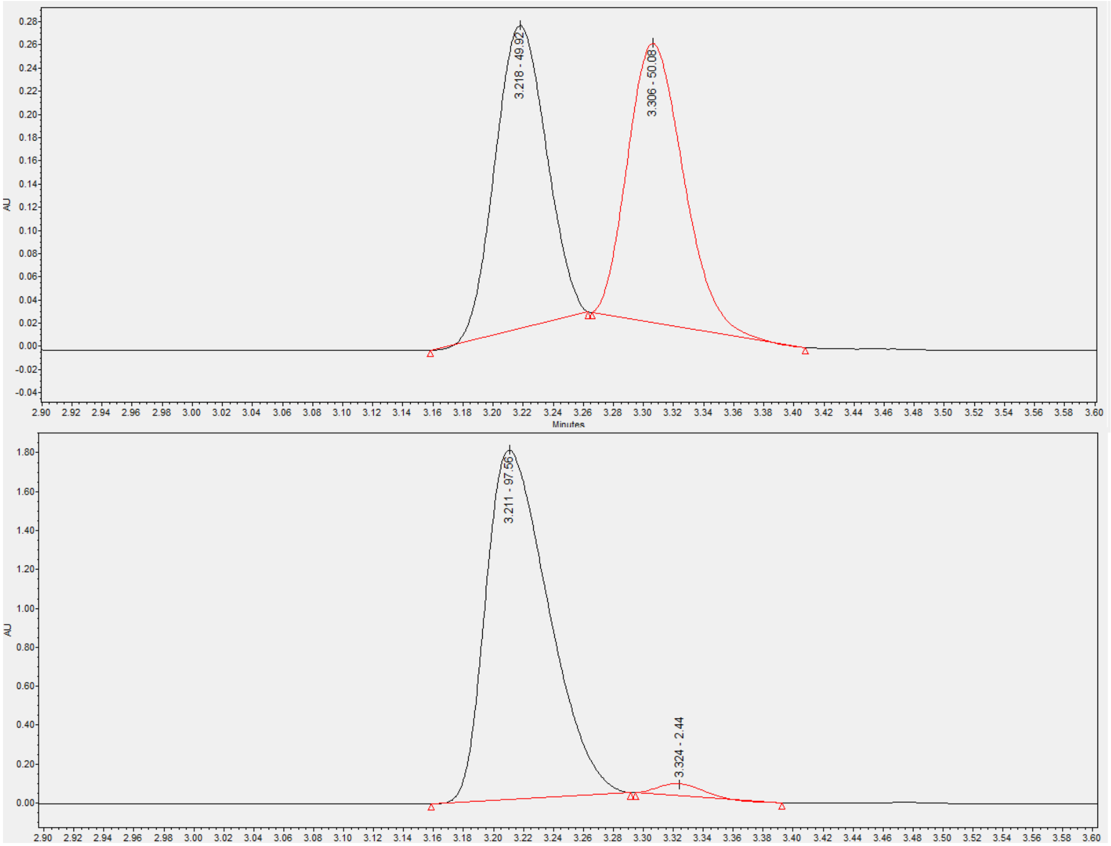

Supplementary Figure 3: ^{1}H, ^{13}C NMR and SFC trace for **2c**

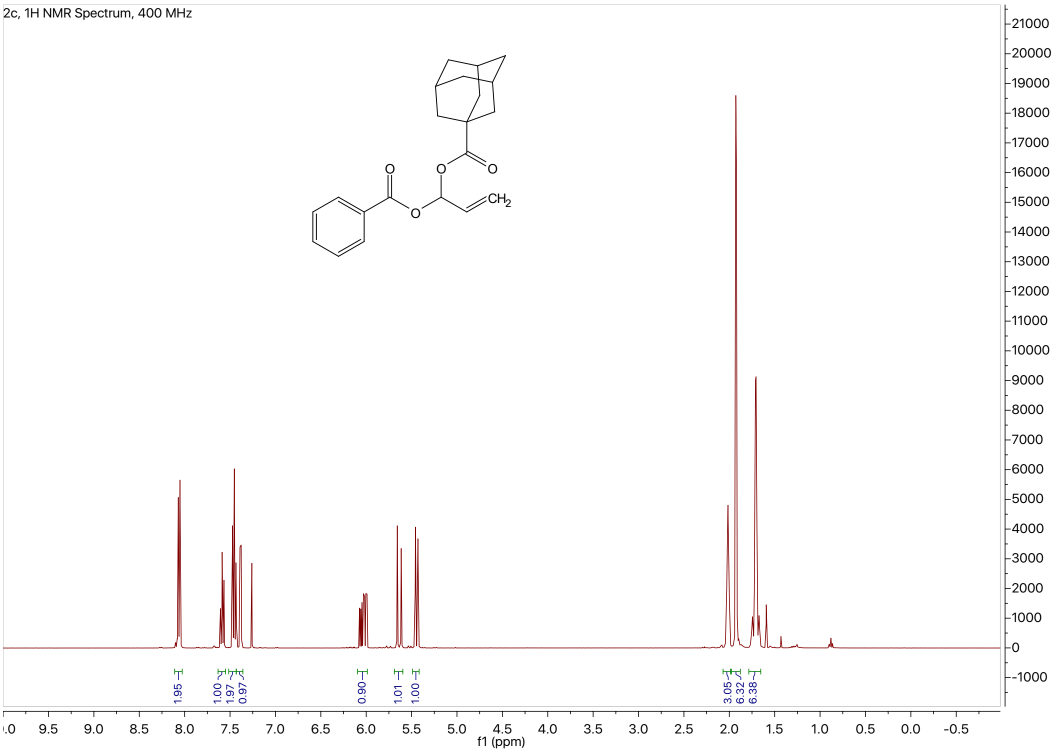

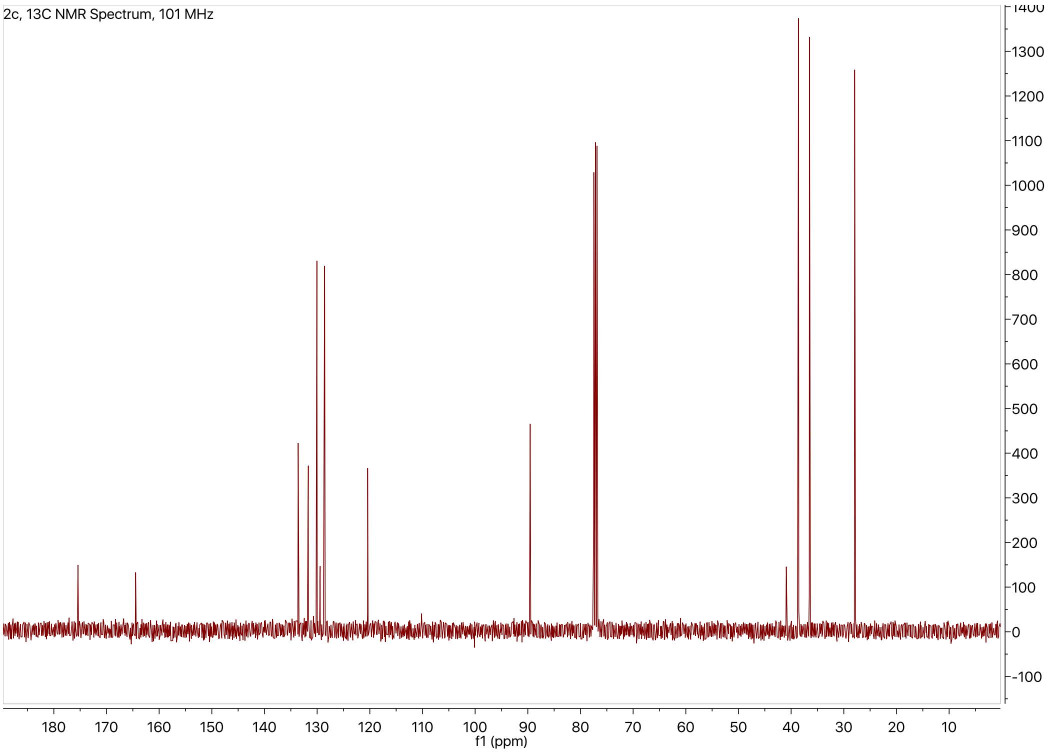
2c, 13C NMR Spectrum, 101 MHz
f1 (ppm)

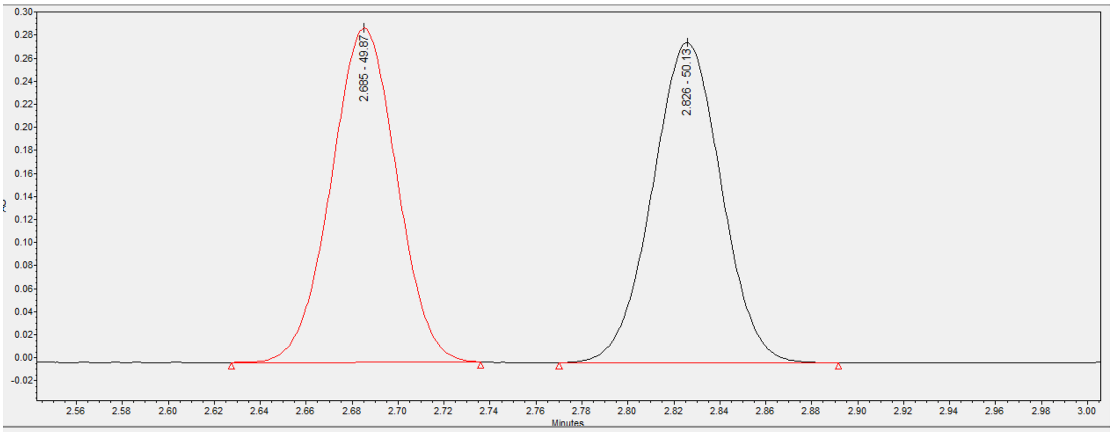
2.685 - 49.87
2.826 - 50.13
Minutes

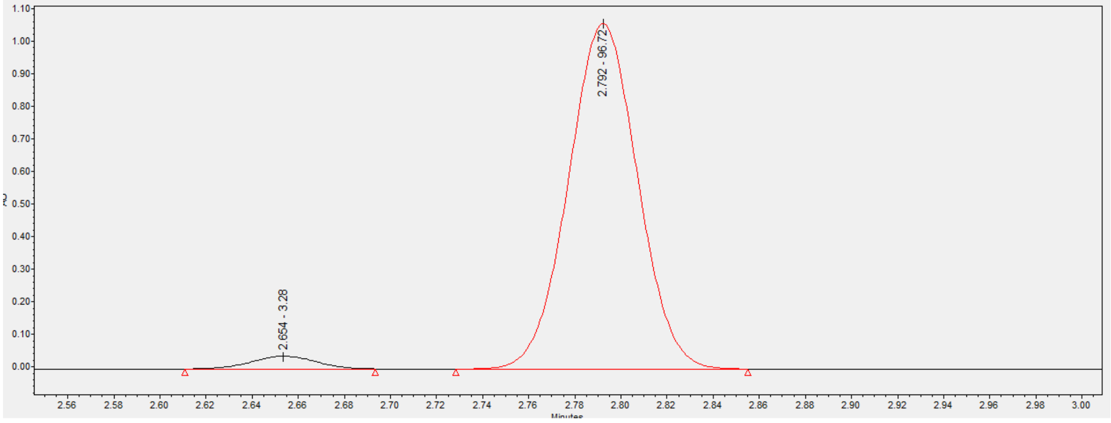
2.654 - 3.28
2.792 - 96.72
Minutes

Supplementary Figure 4: ^{1}H, ^{13}C NMR and SFC trace for **2d**

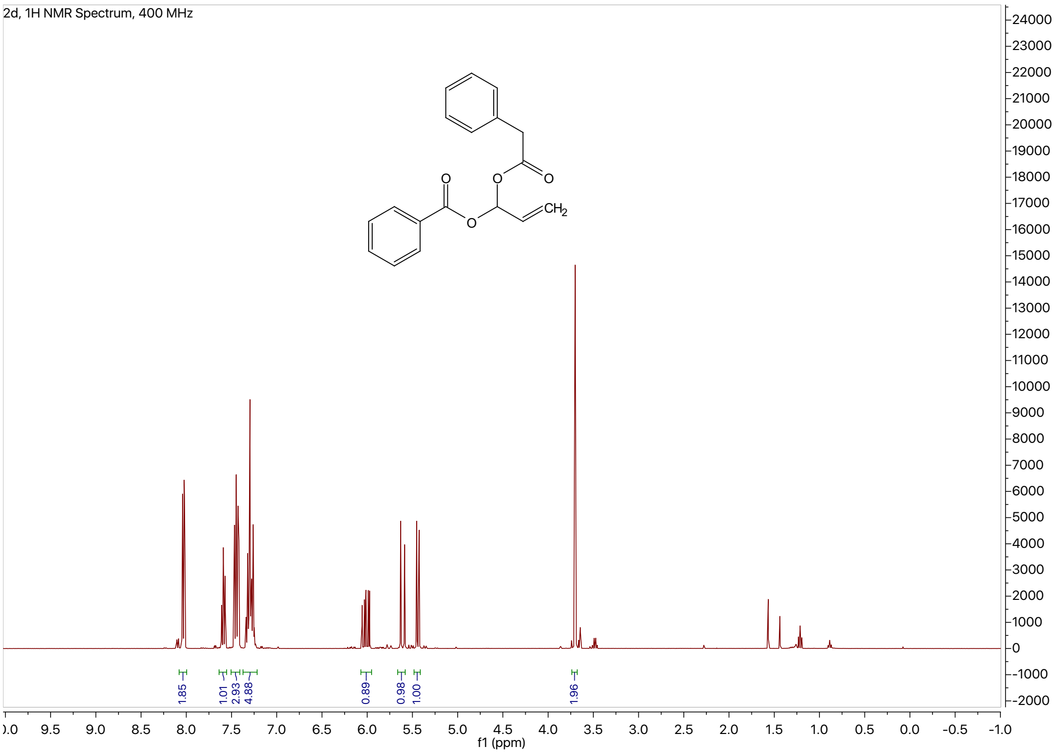

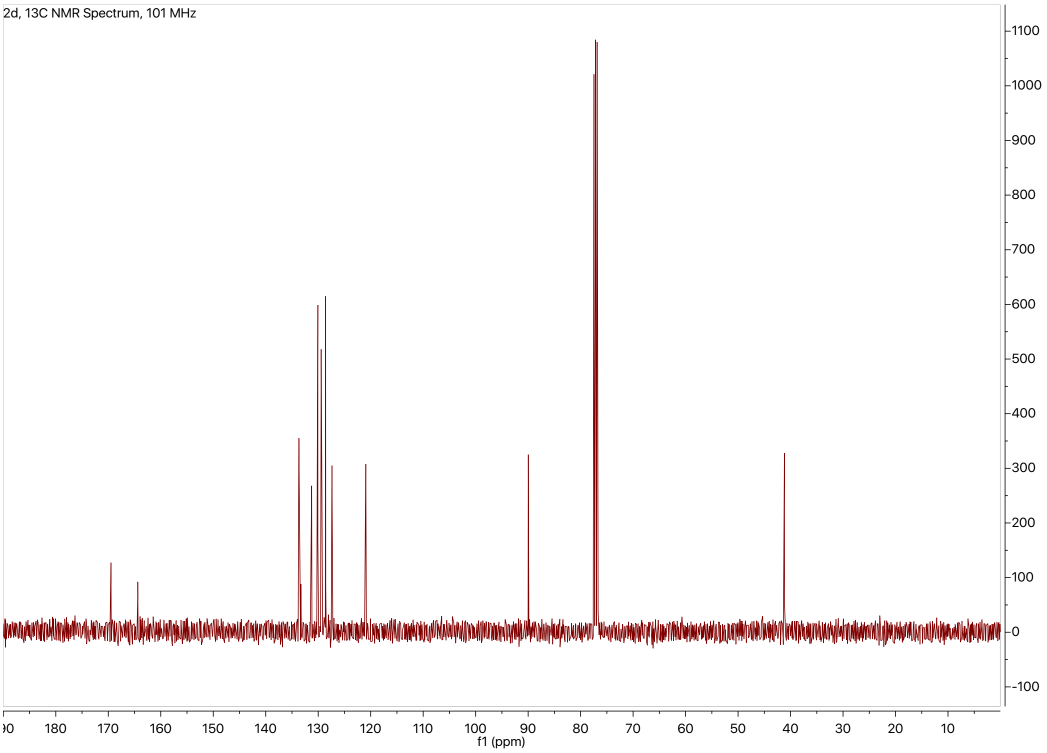

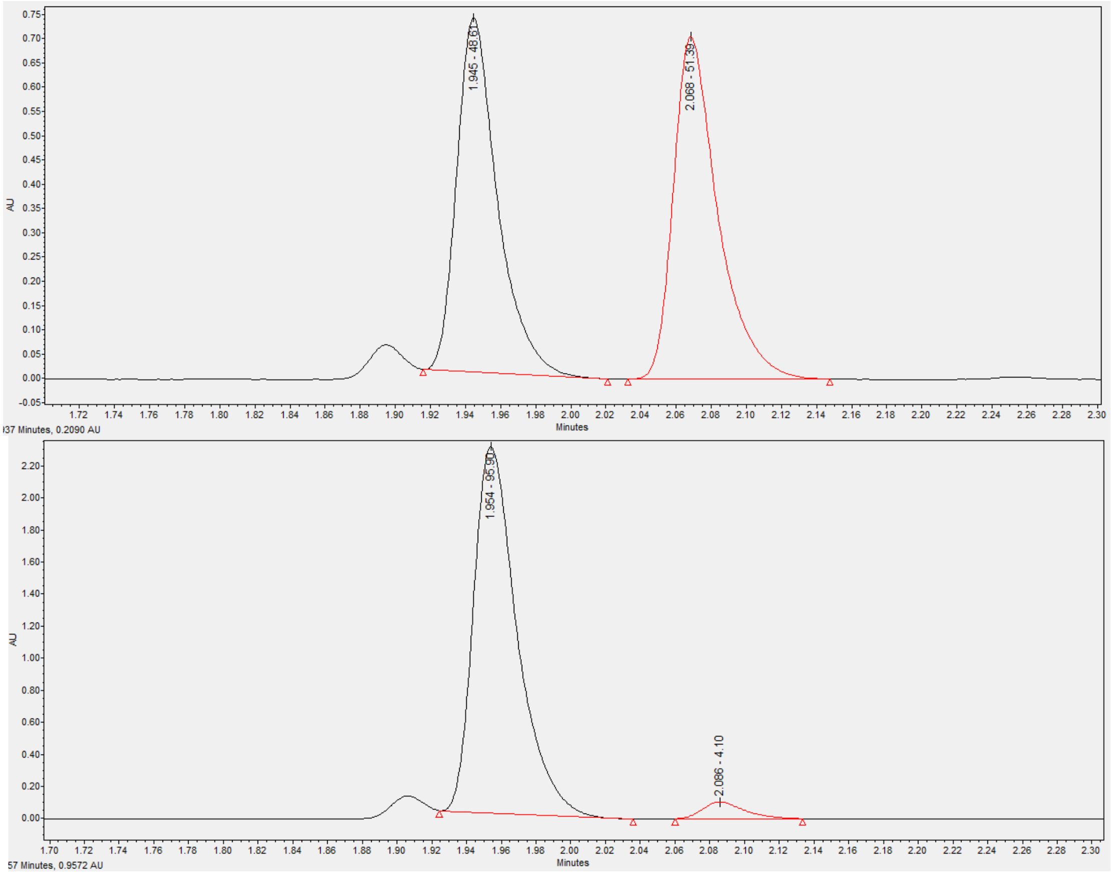

Supplementary Figure 5: ^{1}H, ^{13}C NMR and SFC trace for **2e**

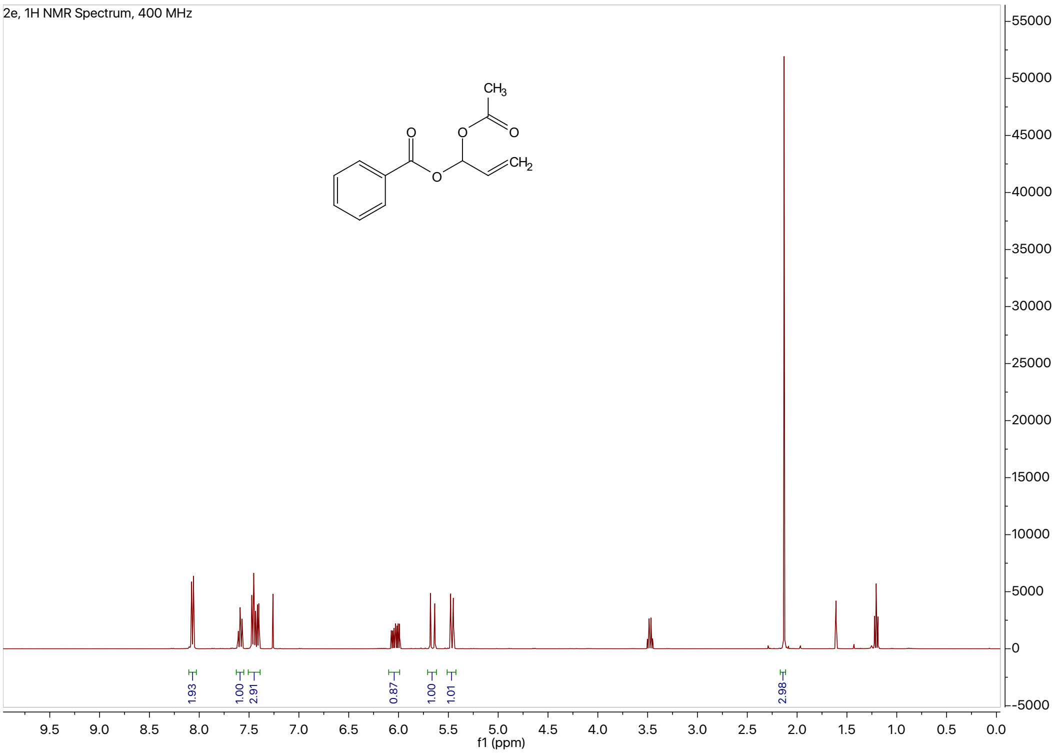

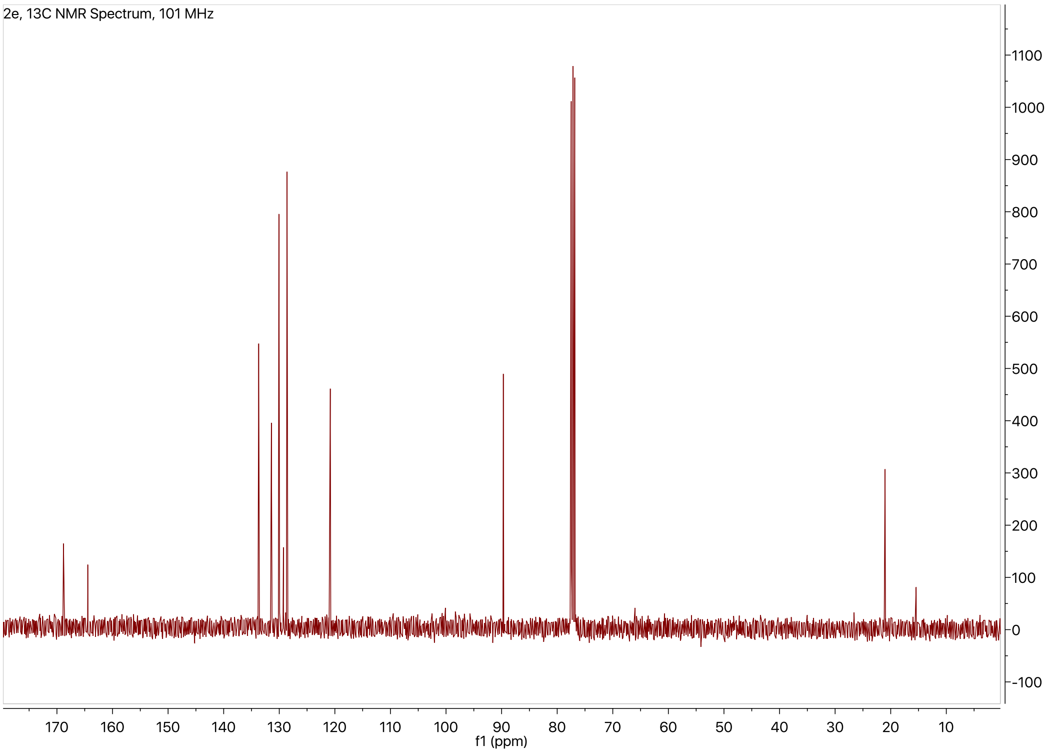
2e, 13C NMR Spectrum, 101 MHz
f1 (ppm)

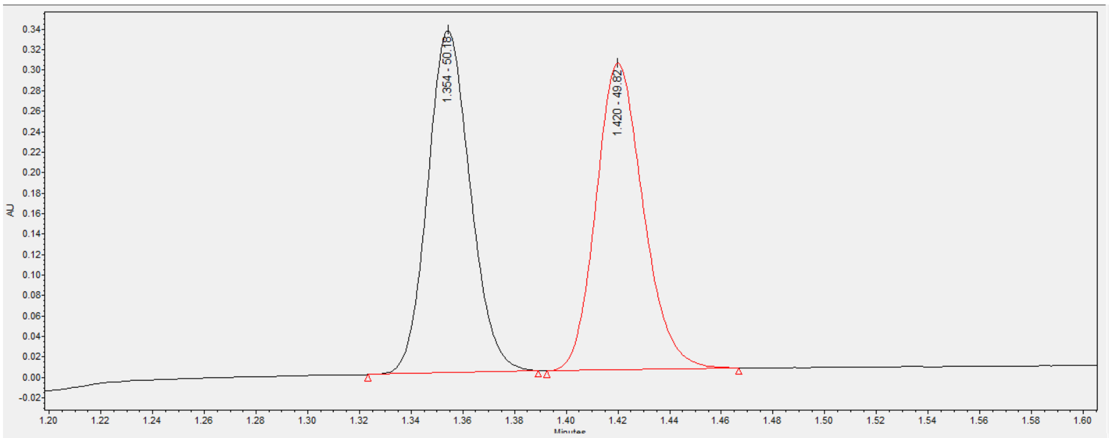
1.354 - 50.18
1.420 - 49.82
AU

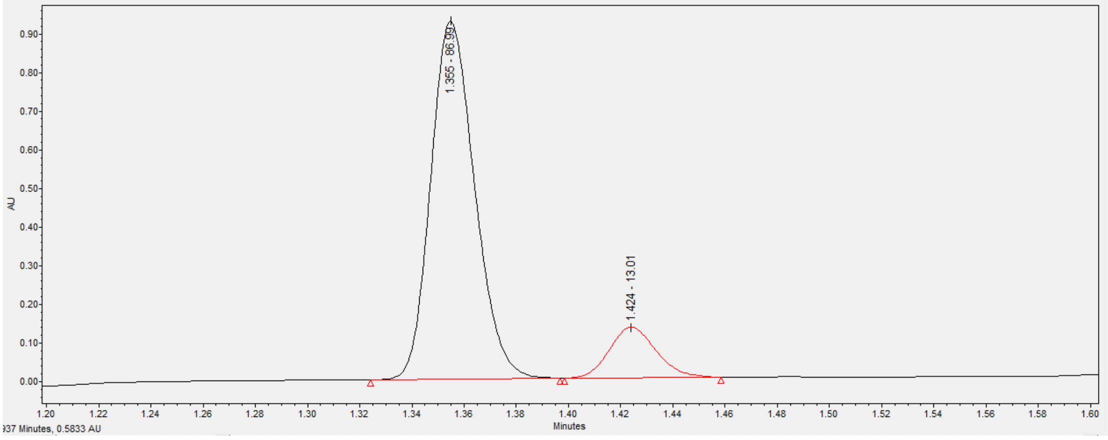
1.355 - 86.99
1.424 - 13.01
AU
Minutes
Minutes, 0.5833 AU

Supplementary Figure 6: ^{1}H, ^{13}C NMR and SFC trace for **2f**

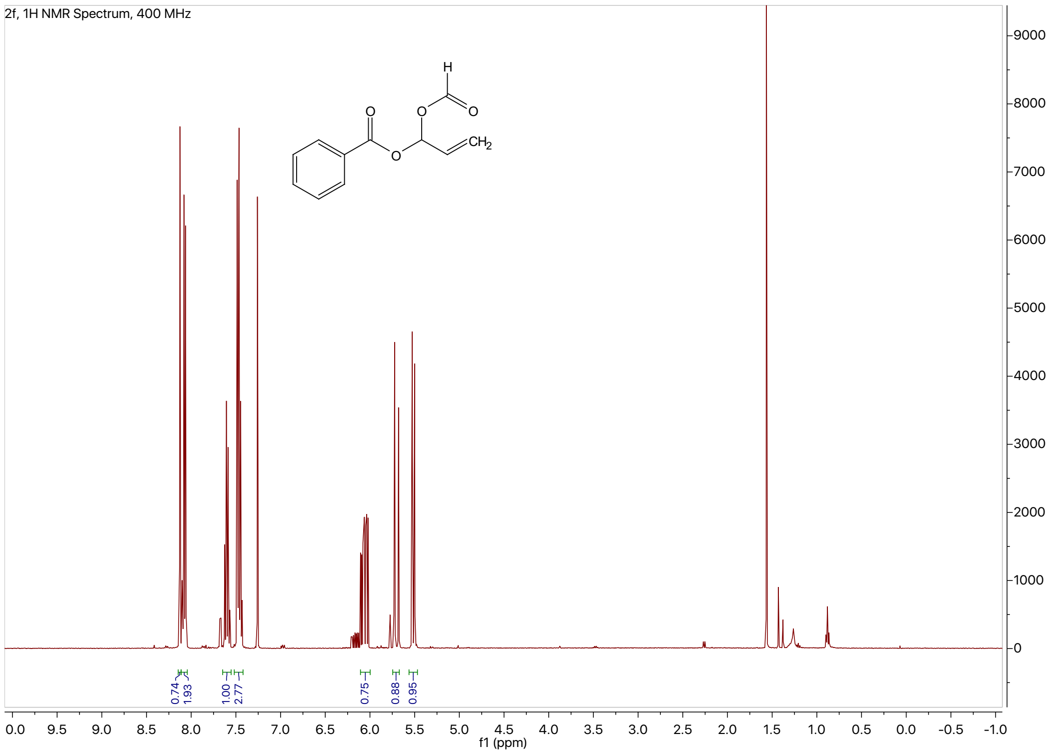

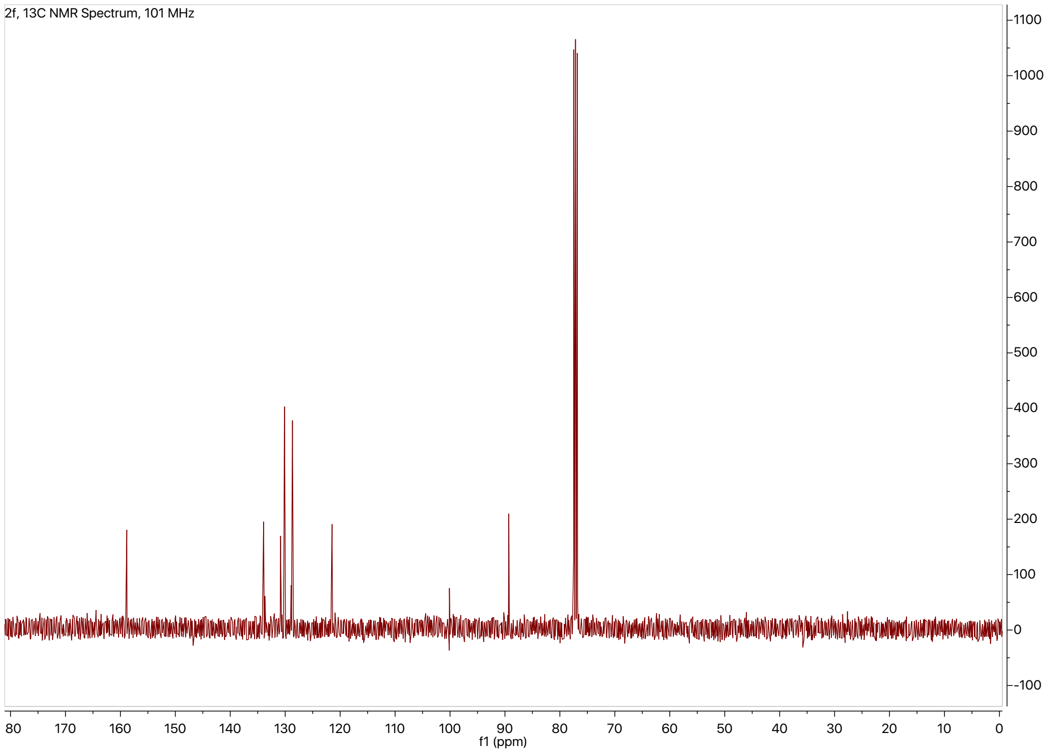

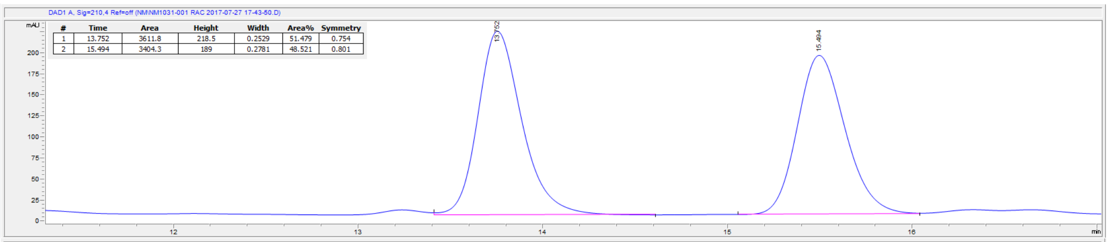

#	Time	Area	Height	Width	Area%	Symmetry
1	13.752	3611.8	218.5	0.2529	51.479	0.754
2	15.494	3404.3	189	0.2781	48.521	0.801

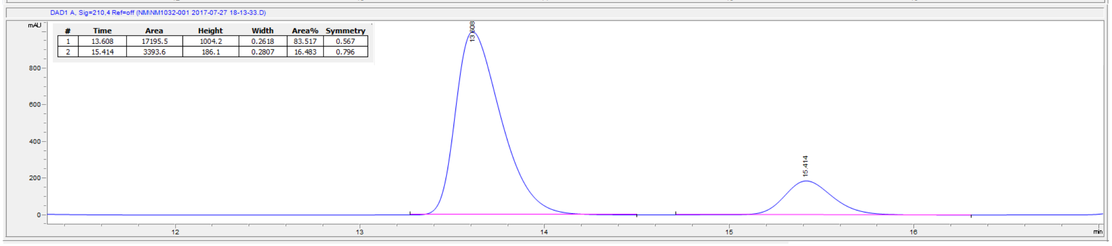

#	Time	Area	Height	Width	Area%	Symmetry
1	13.608	17195.5	1004.2	0.2618	83.517	0.567
2	15.414	3393.6	186.1	0.2807	16.483	0.796

Supplementary Figure 7: ^{1}H, ^{13}C NMR and SFC trace for **2g**

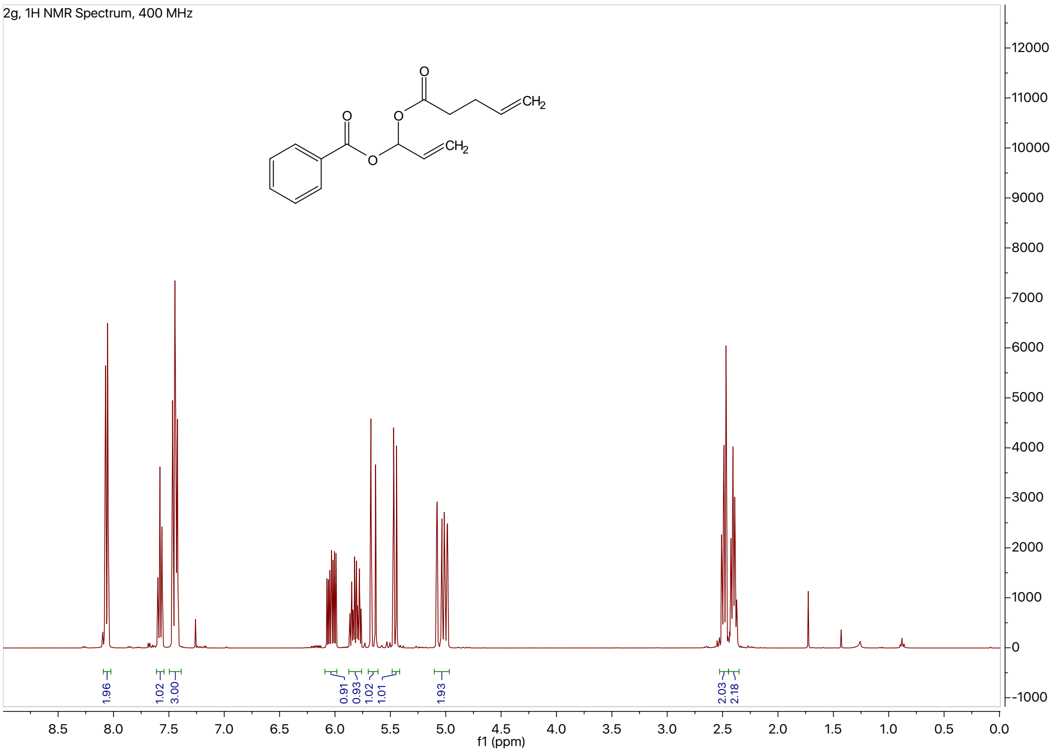

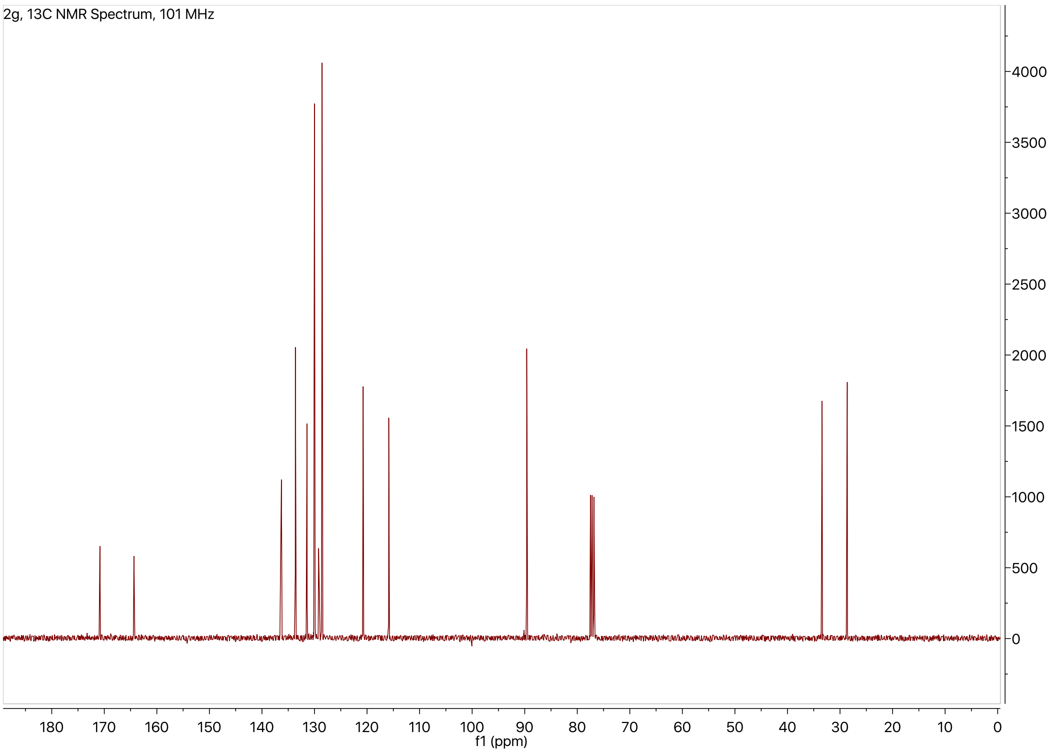
2g, 13C NMR Spectrum, 101 MHz
f1 (ppm)

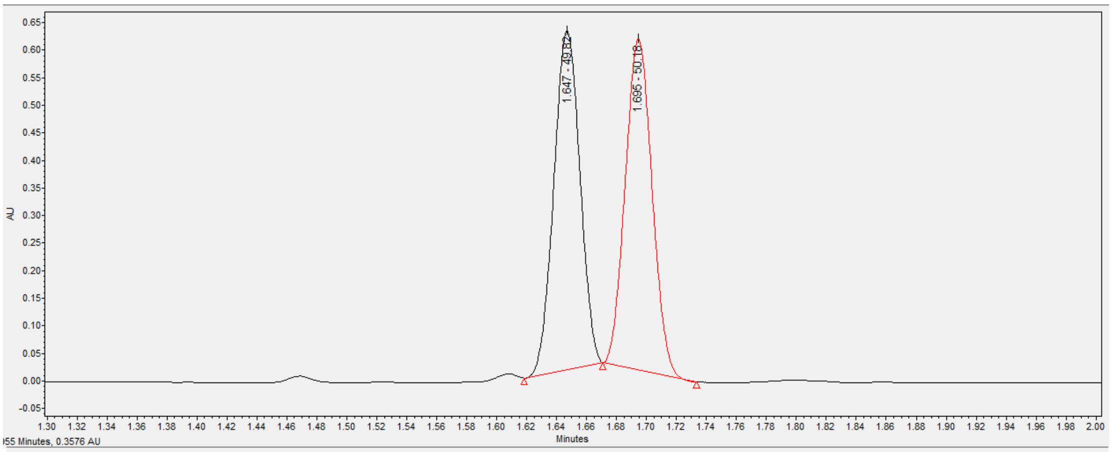
1.647 - 49.82
1.695 - 50.18
AU
Minutes
55 Minutes, 0.3576 AU

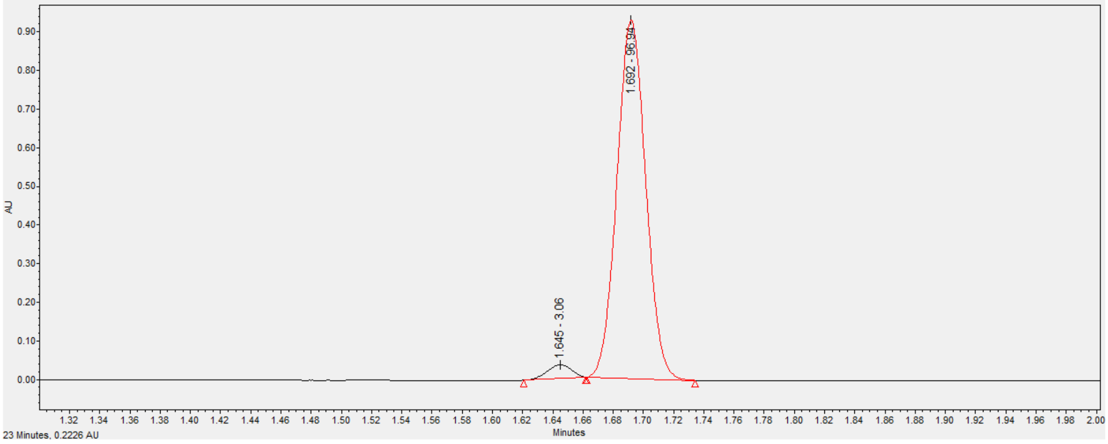
1.645 - 3.06
1.692 - 96.94
AU
Minutes
23 Minutes, 0.2226 AU

Supplementary Figure 8: ^{1}H, ^{13}C NMR and SFC trace for **2h**

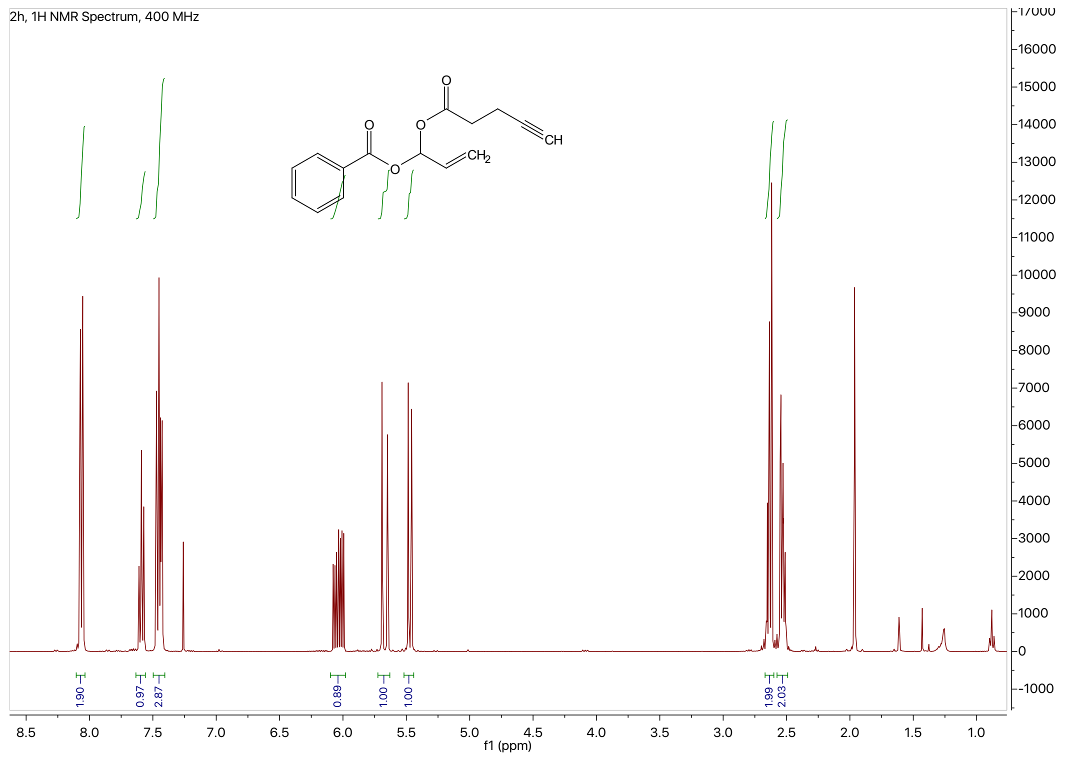

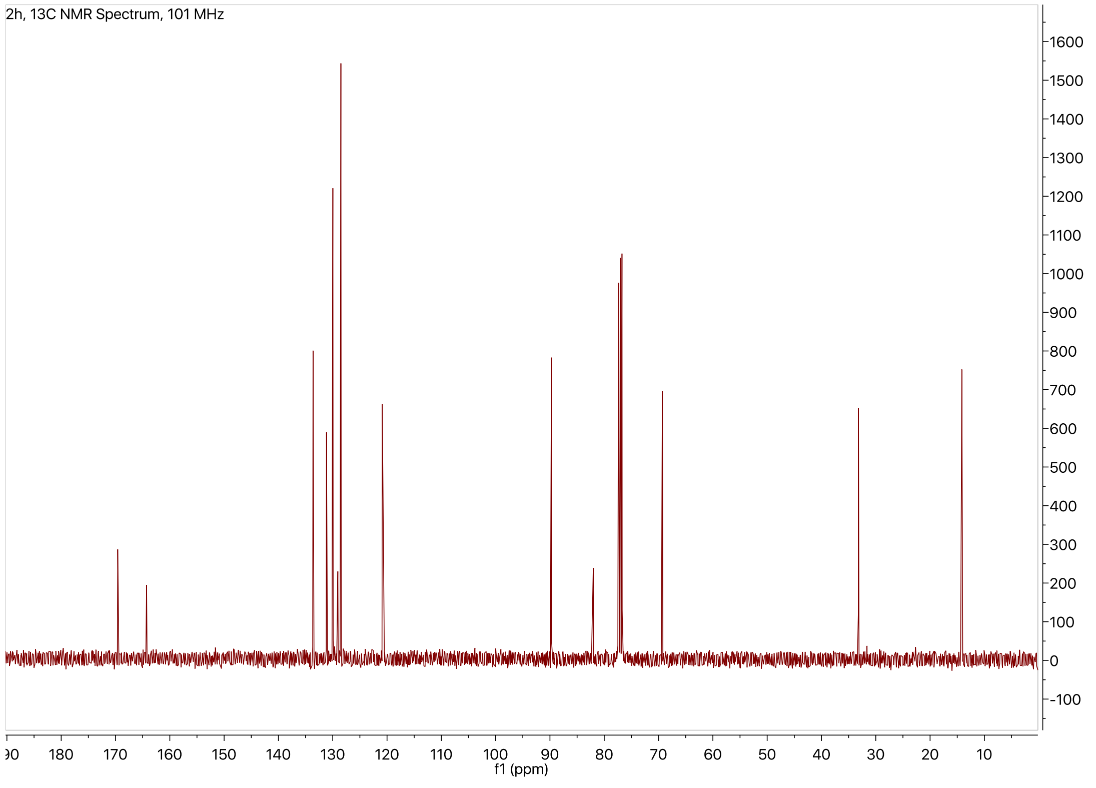

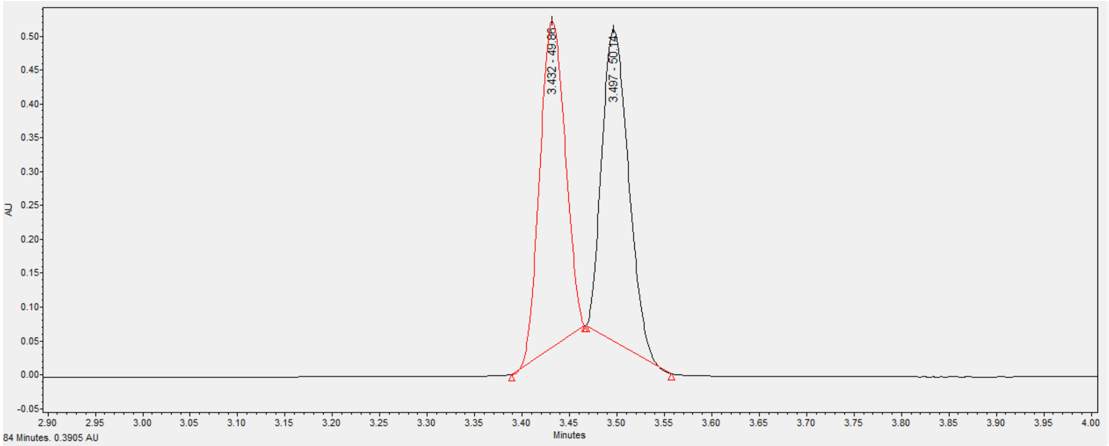

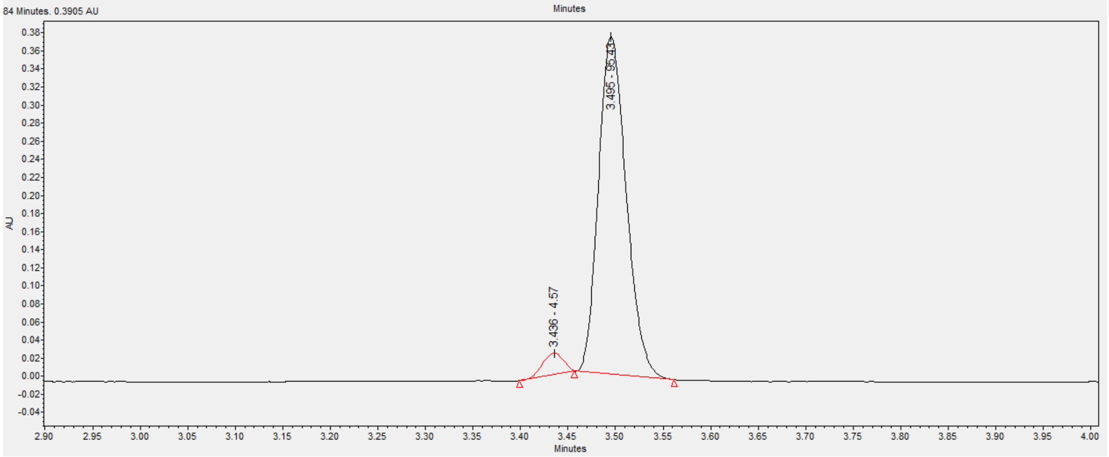

Supplementary Figure 9: ^{1}H, ^{13}C NMR and SFC trace for **2i**

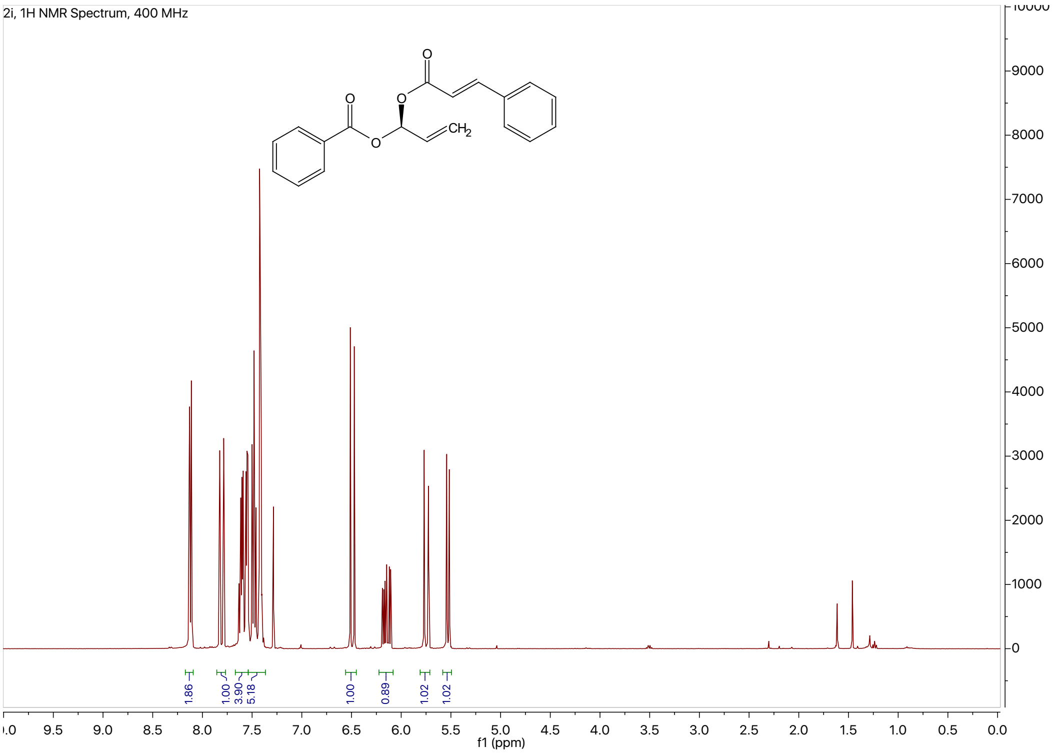

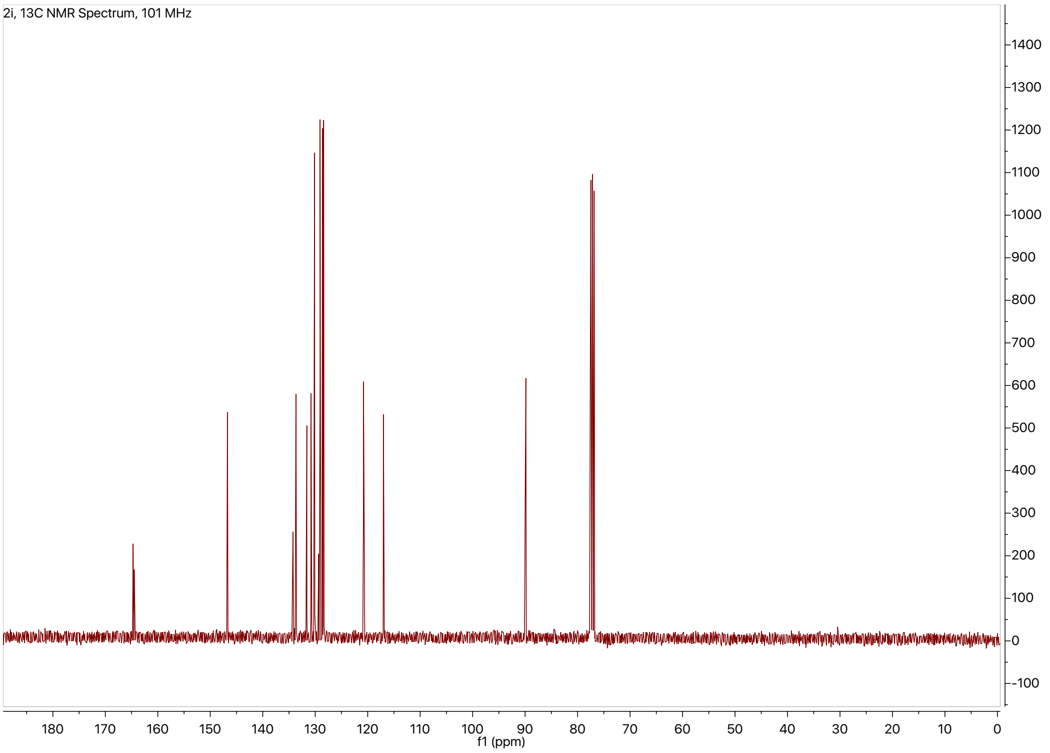
2i, 13C NMR Spectrum, 101 MHz
f1 (ppm)

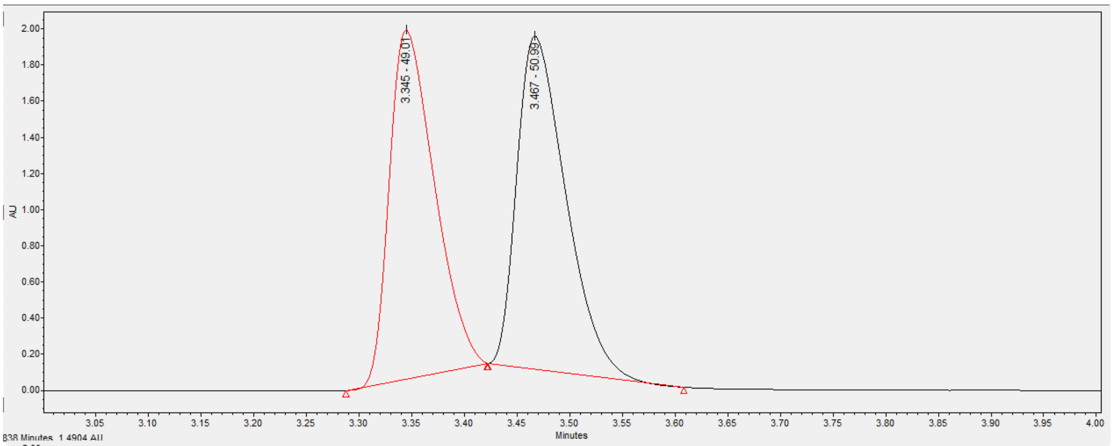
3.345 - 49.01
3.467 - 50.99
AU
Minutes

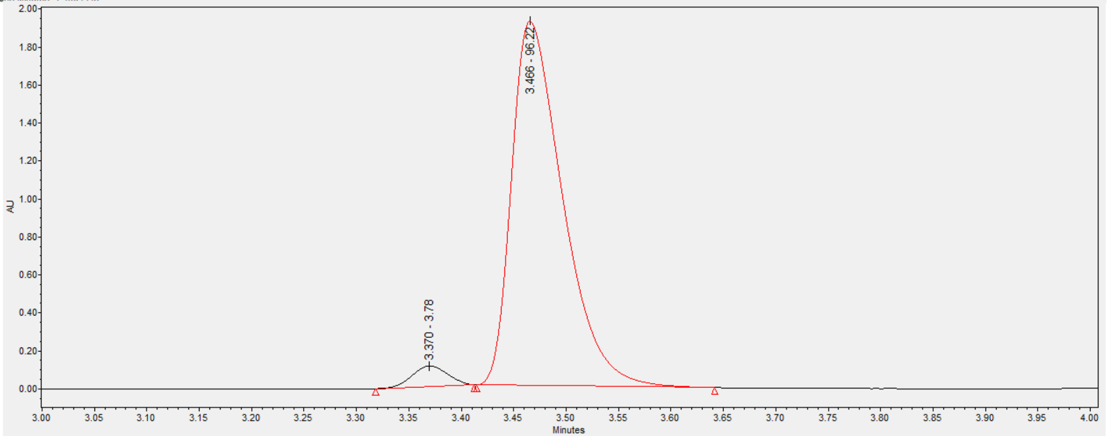
3.370 - 3.78
3.466 - 96.22
AU
Minutes

Supplementary Figure 10: ^{1}H, ^{13}C NMR and SFC trace for **2j**

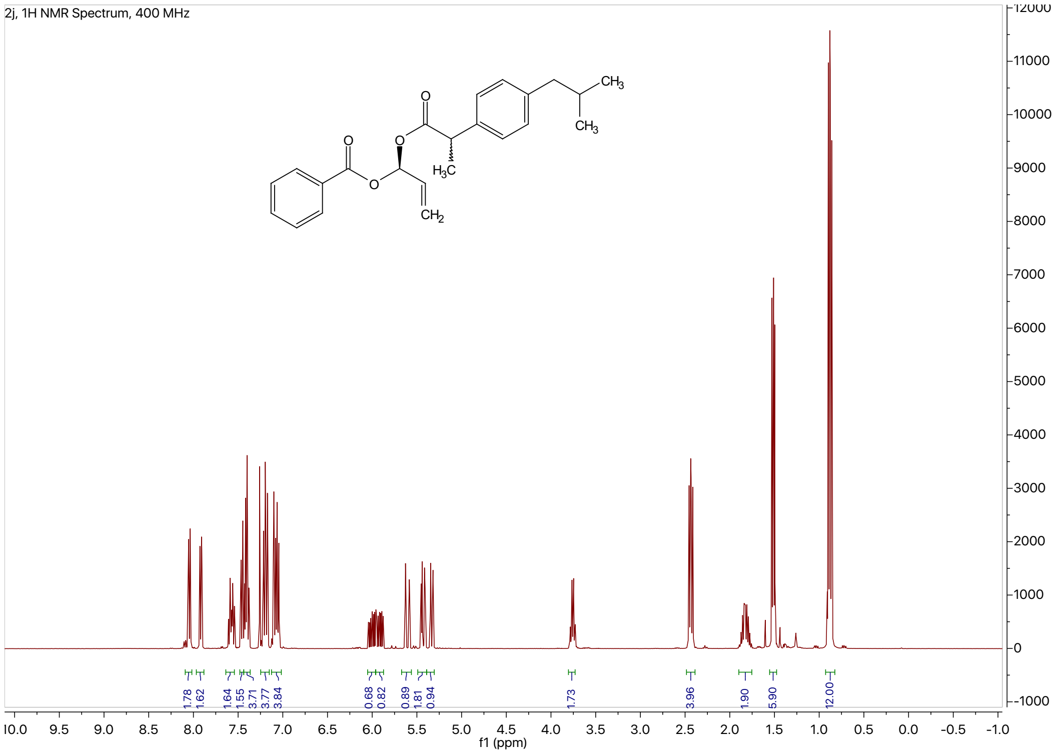

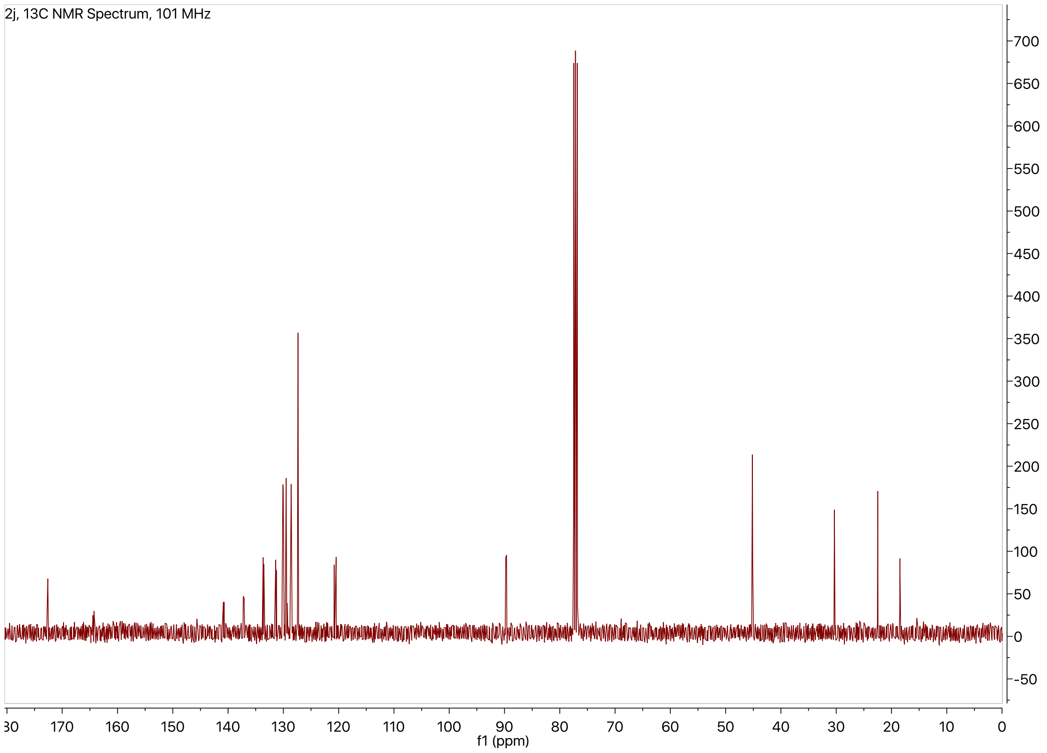

D1:

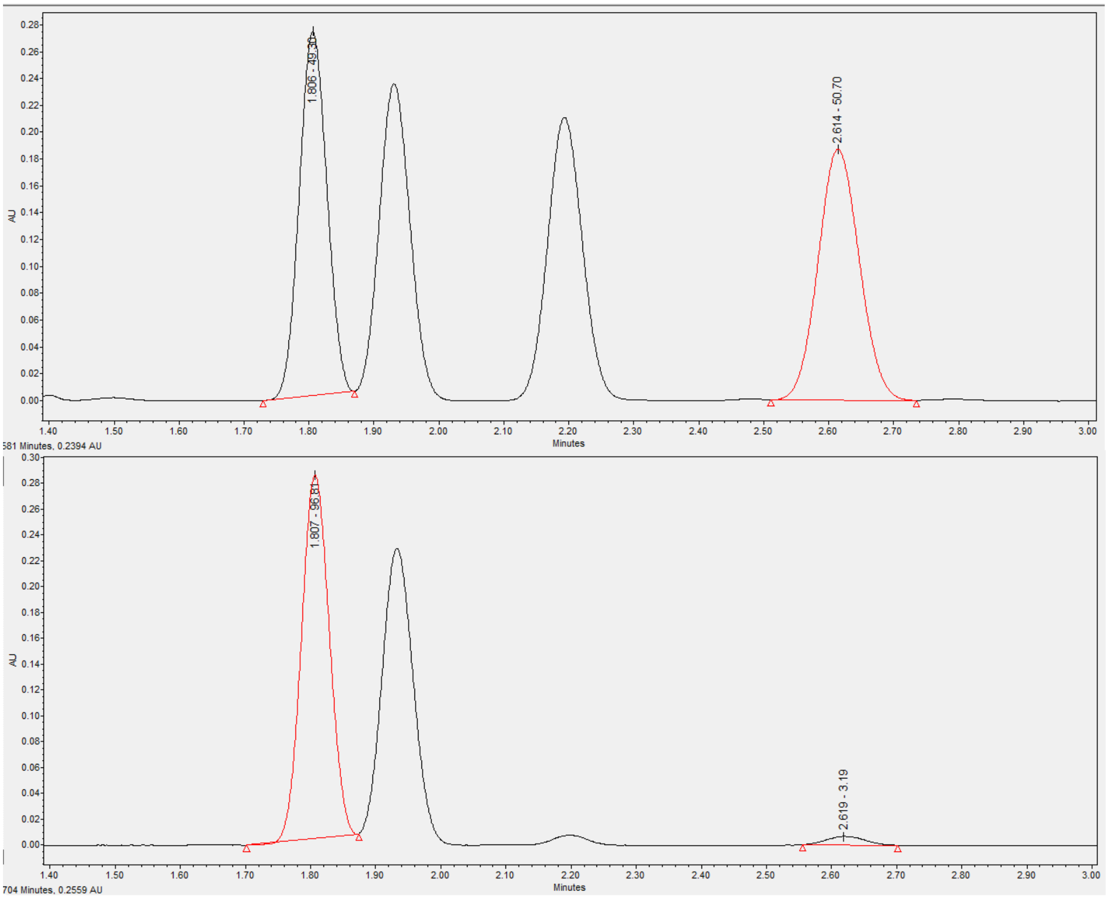

1.806 - 49.30
2.614 - 50.70
AU
Minutes
581 Minutes, 0.2394 AU
1.807 - 96.81
2.619 - 3.19
AU
Minutes
704 Minutes, 0.2559 AU

D2:

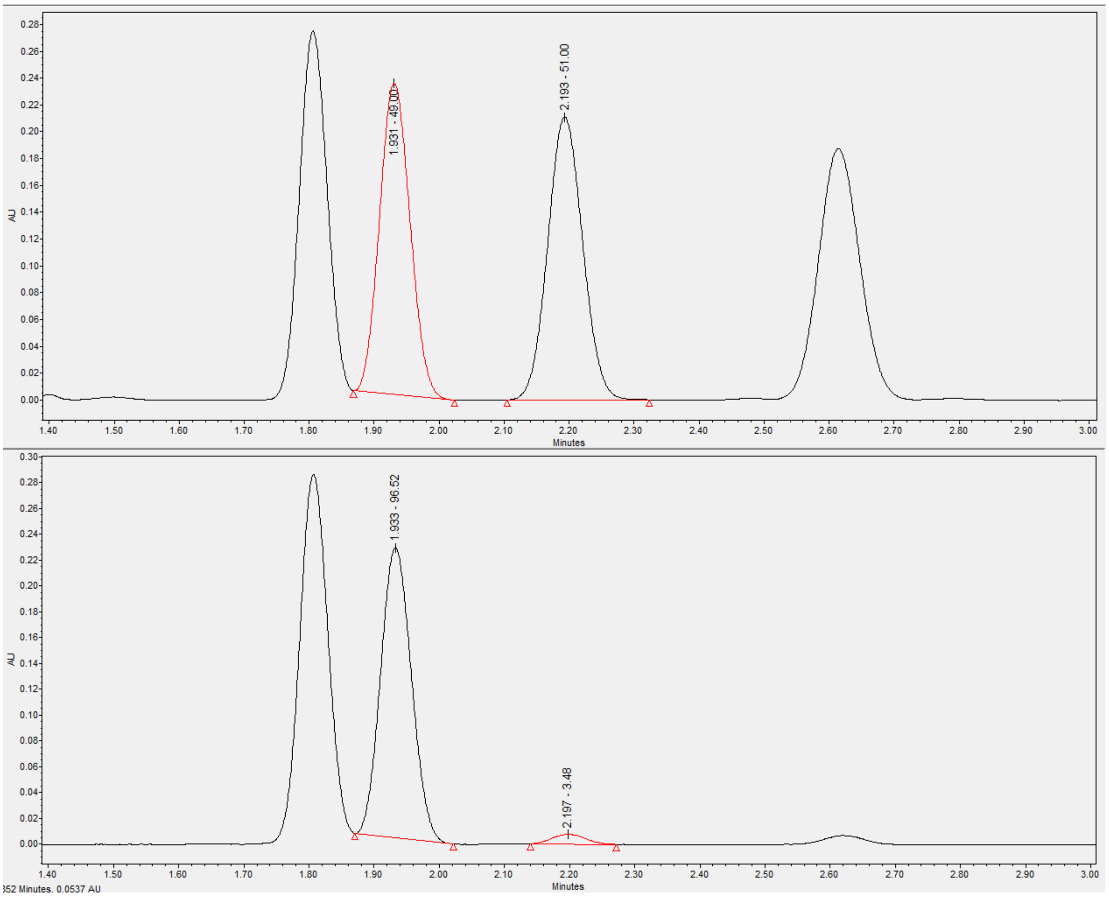

1.931 - 49.00
2.193 - 51.00
AU
Minutes
1.933 - 96.52
2.197 - 3.48
AU
Minutes
352 Minutes, 0.0537 AU

Supplementary Figure 11: ^{1}H, ^{13}C NMR and SFC trace for **2k**

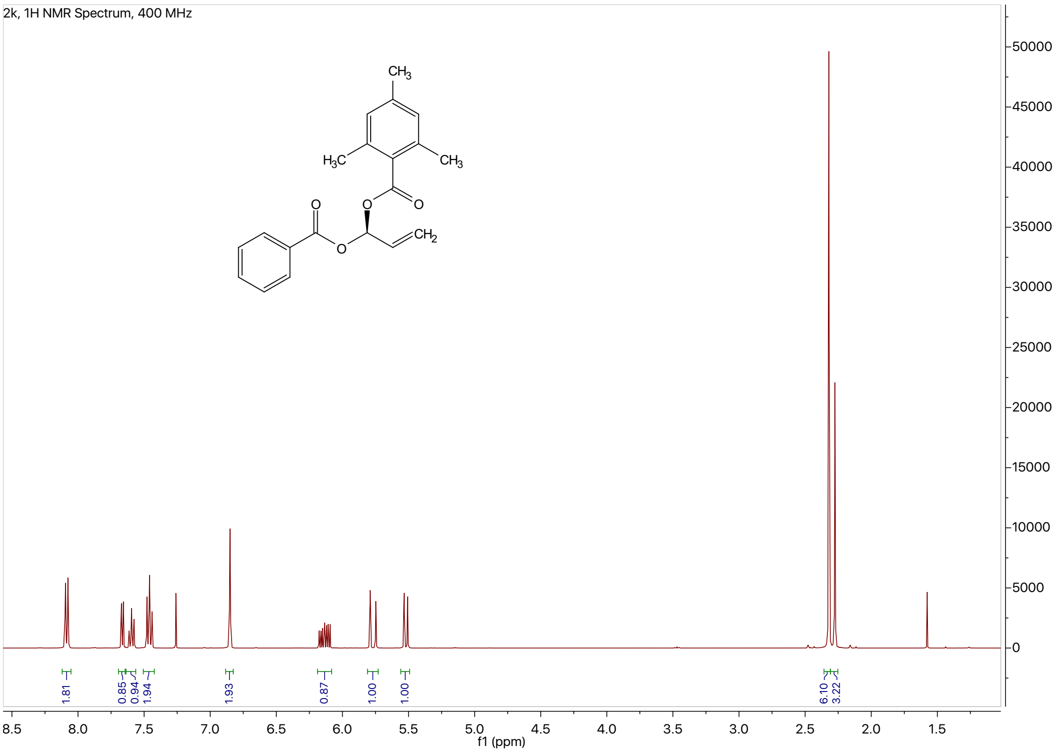

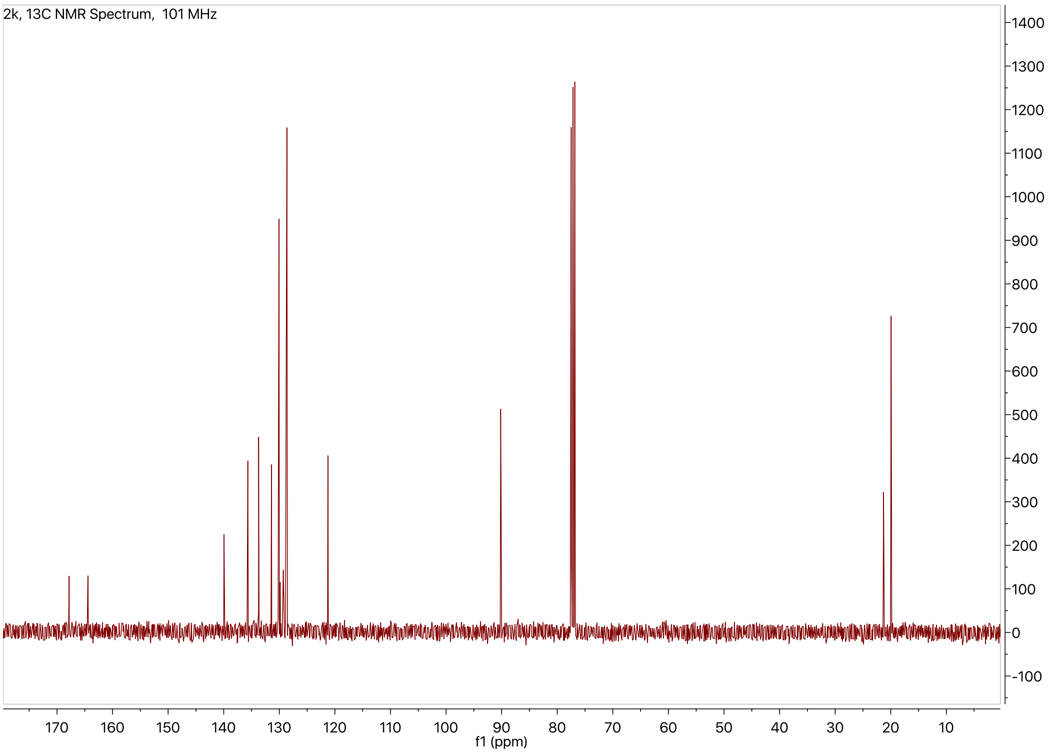

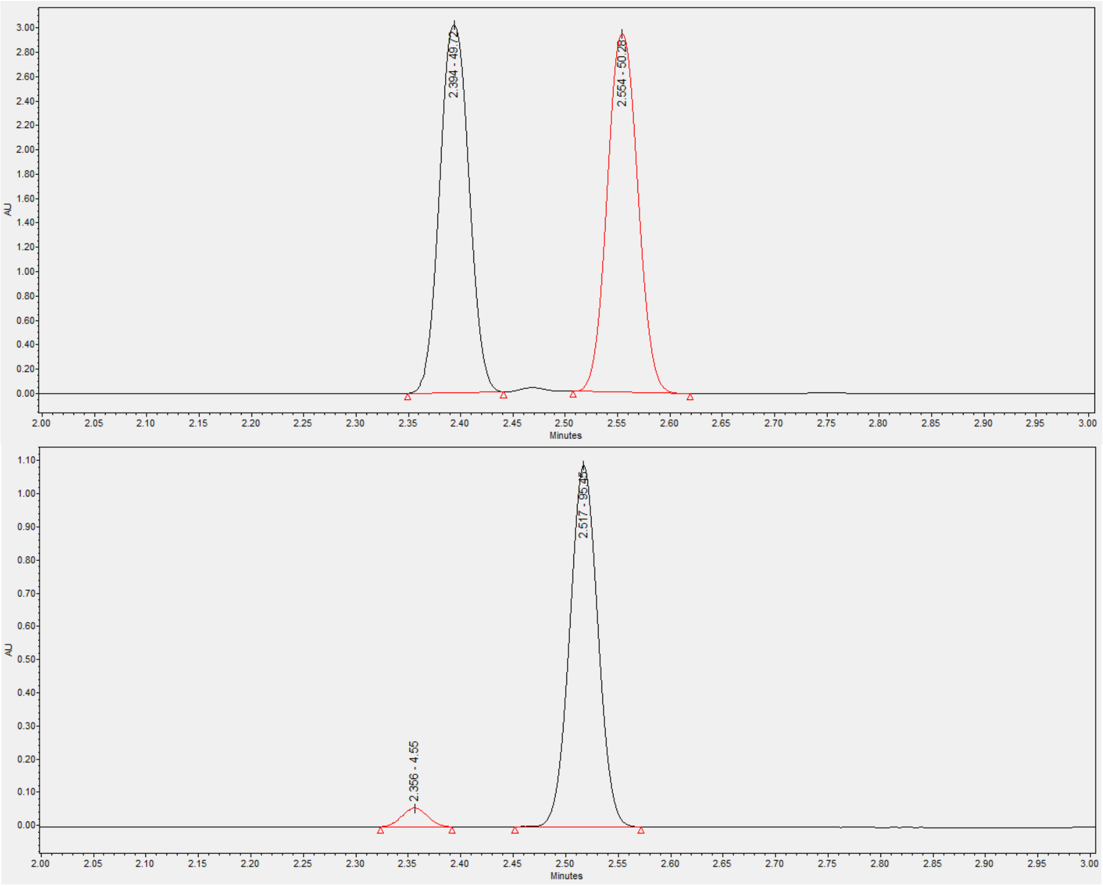

Supplementary Figure 12: ^{1}H, ^{13}C NMR and SFC trace for **2l**

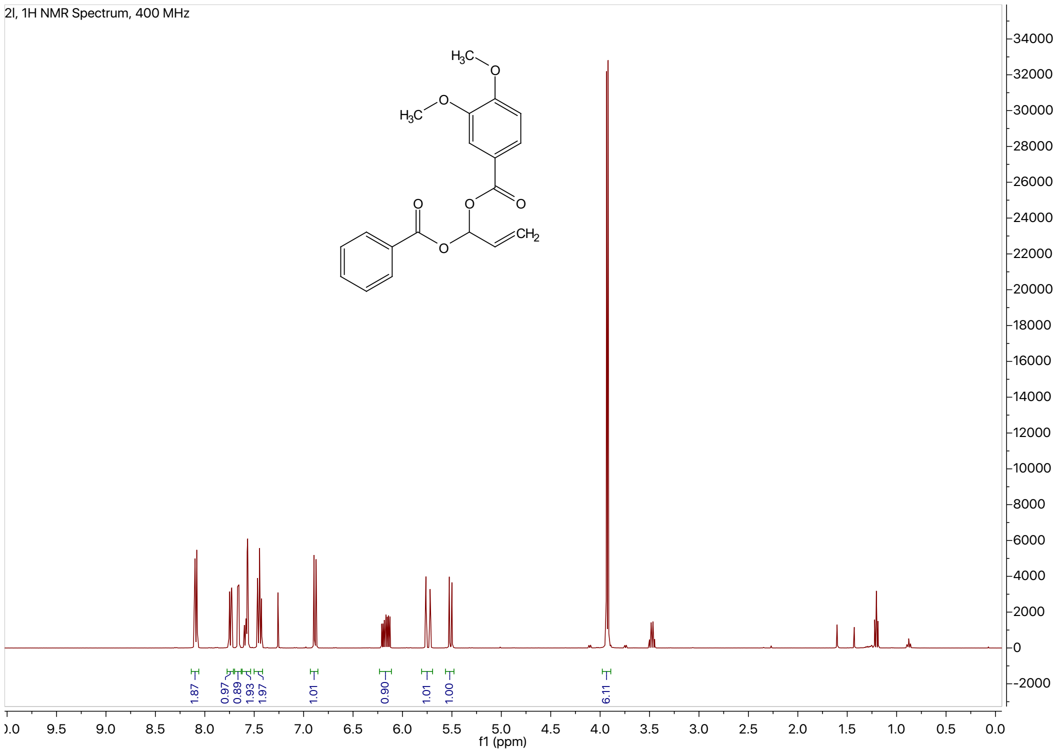

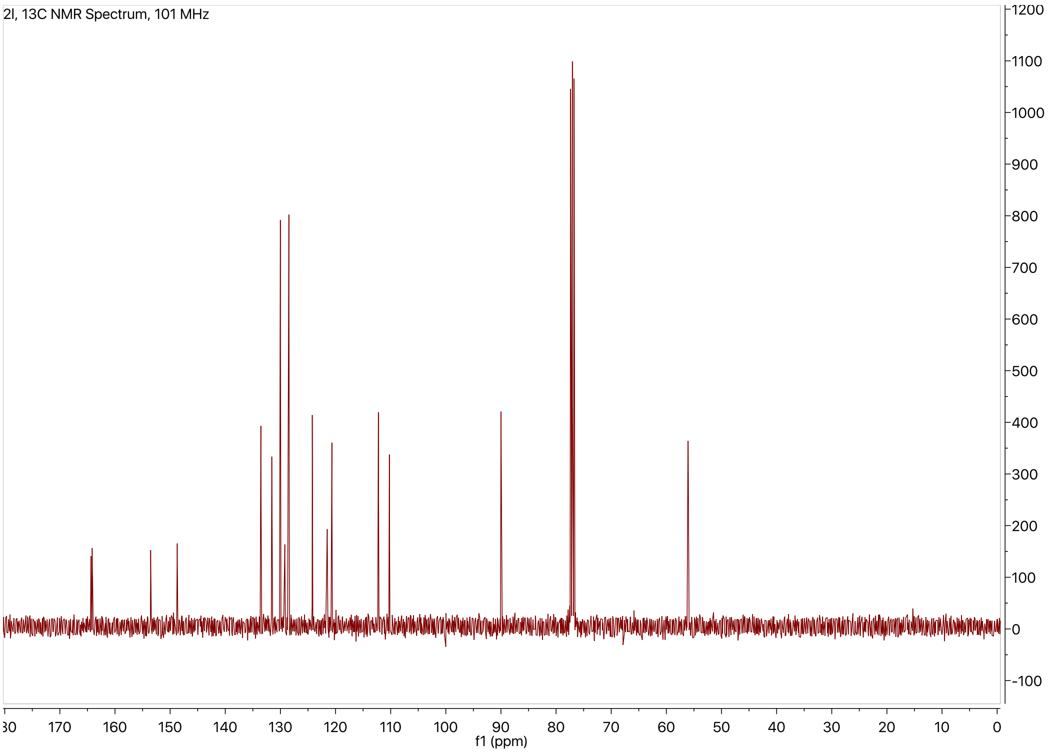
2l, 13C NMR Spectrum, 101 MHz
f1 (ppm)

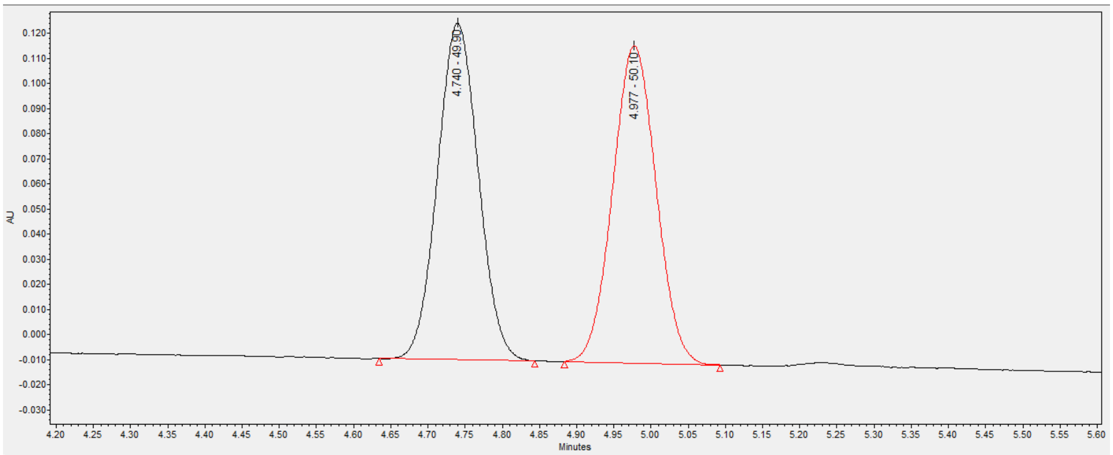
4.740 - 49.90
4.977 - 50.10
AU
Minutes

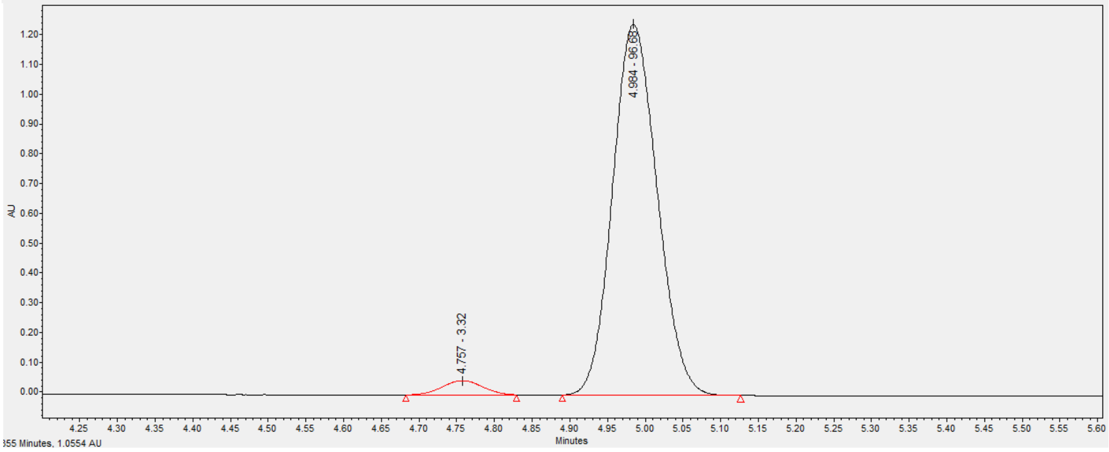
4.984 - 96.68
4.757 - 3.32
AU
Minutes
355 Minutes, 1.0554 AU

Supplementary Figure 13: ^{1}H, ^{13}C, ^{19}F NMR and SFC trace for **2m**

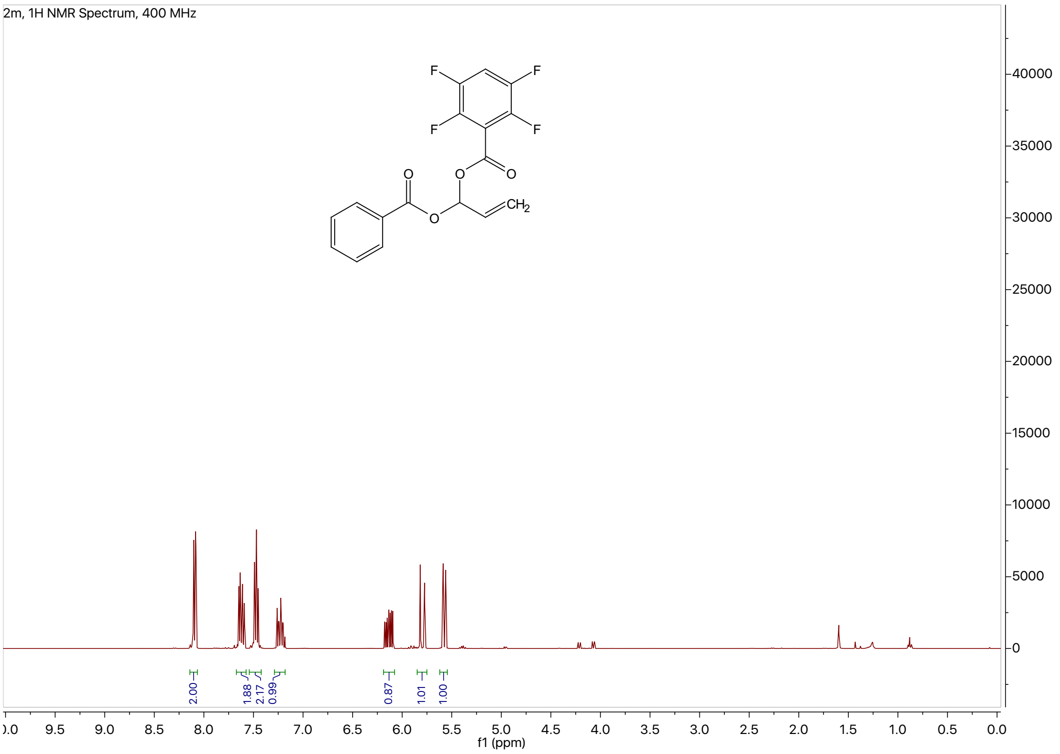

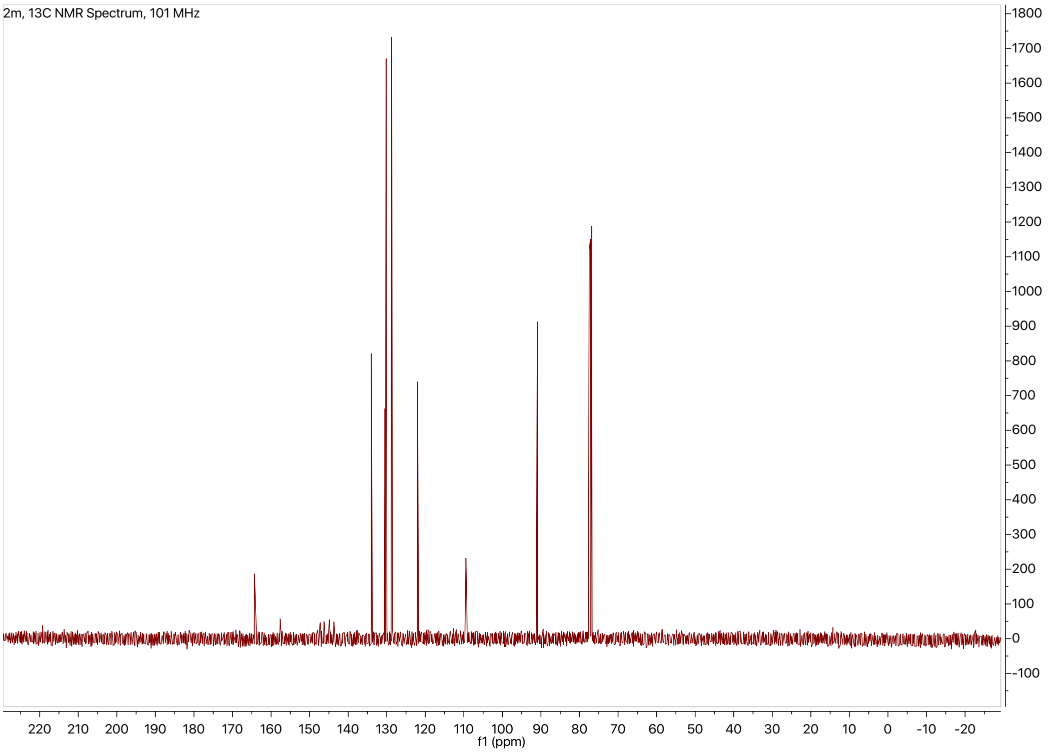

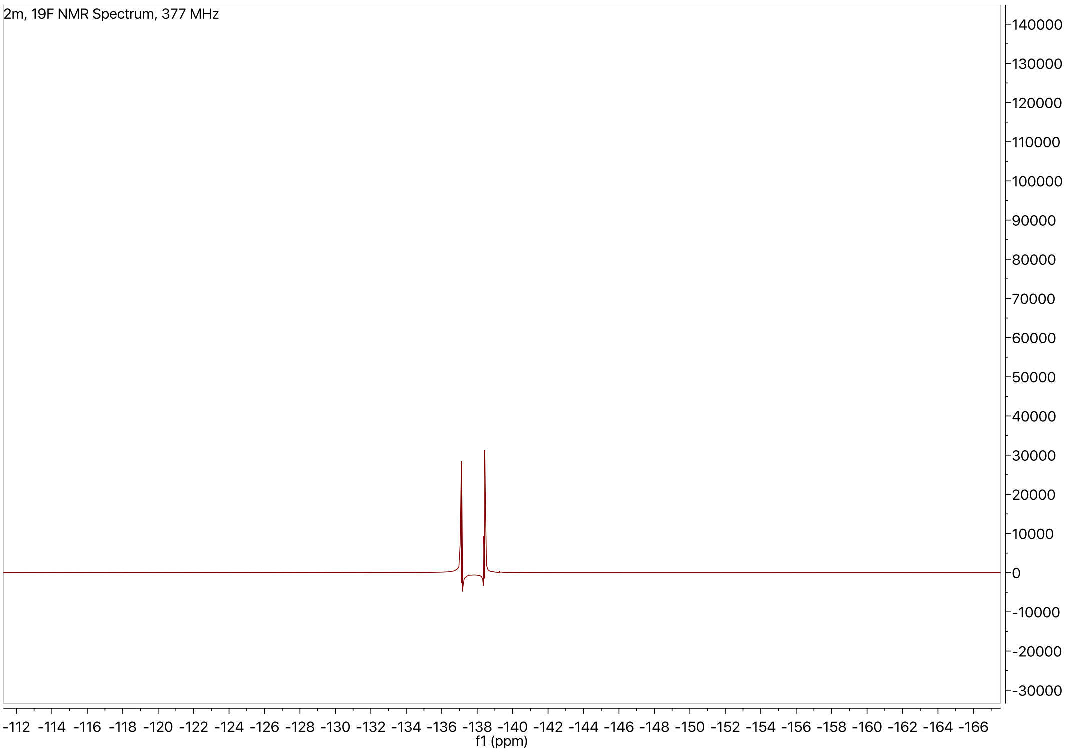
2m, 19F NMR Spectrum, 377 MHz
f1 (ppm)

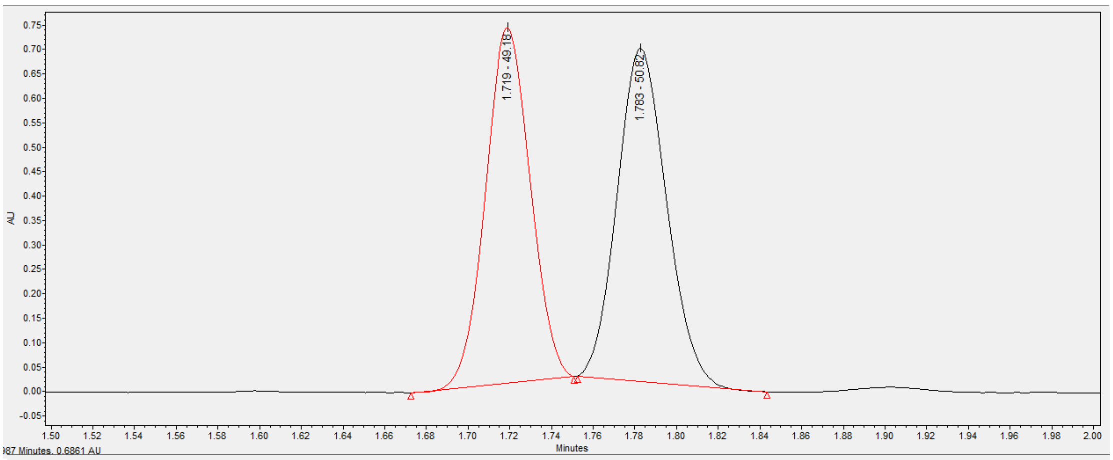
1.719 - 49.18
1.783 - 50.82
AU
Minutes

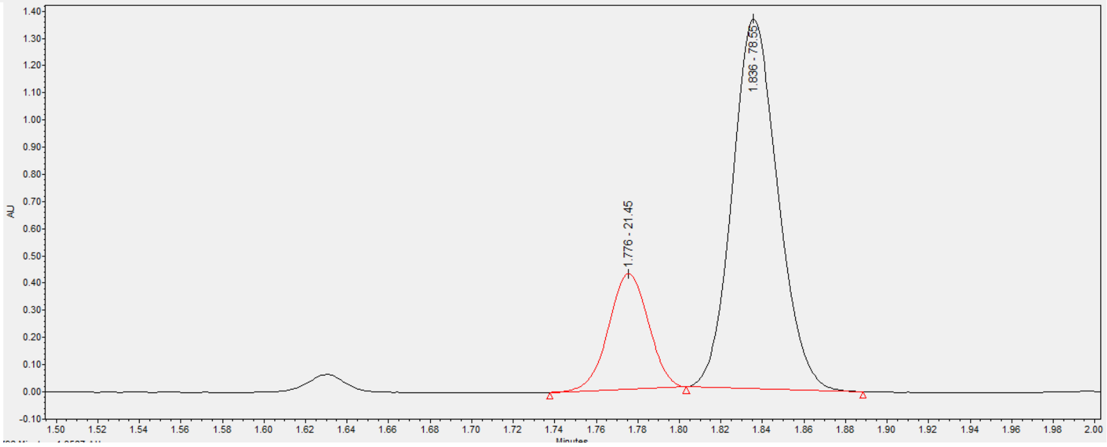
1.776 - 21.45
1.836 - 78.55
AU

Supplementary Figure 14: ^{1}H, ^{13}C NMR and SFC trace for **2n**

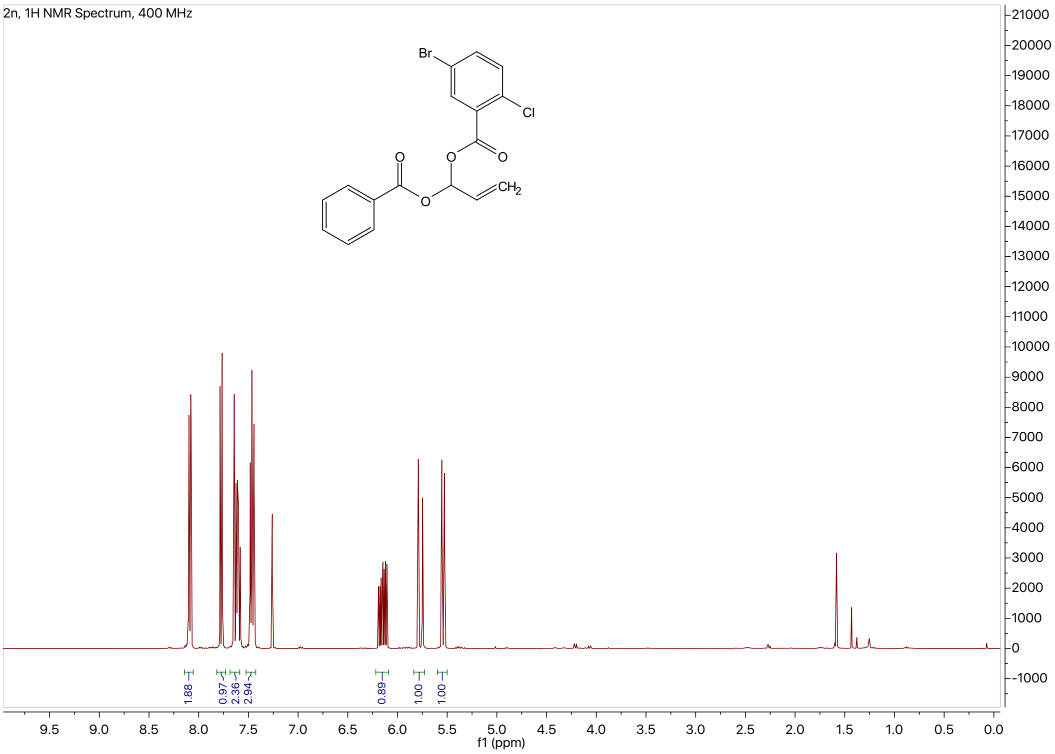

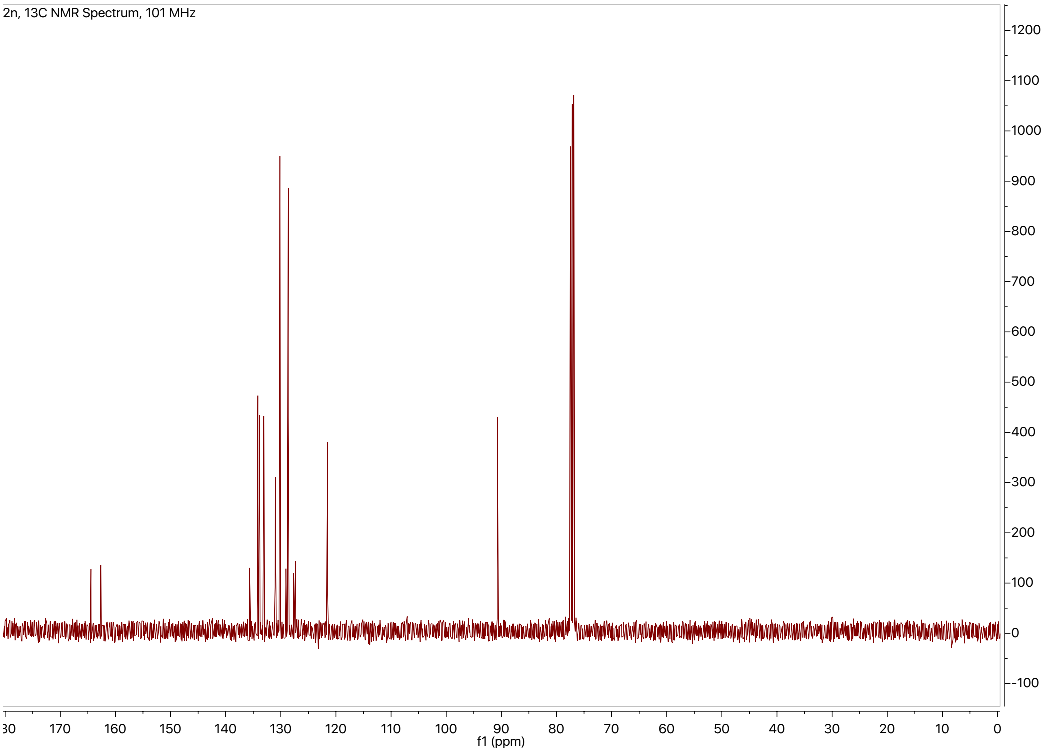

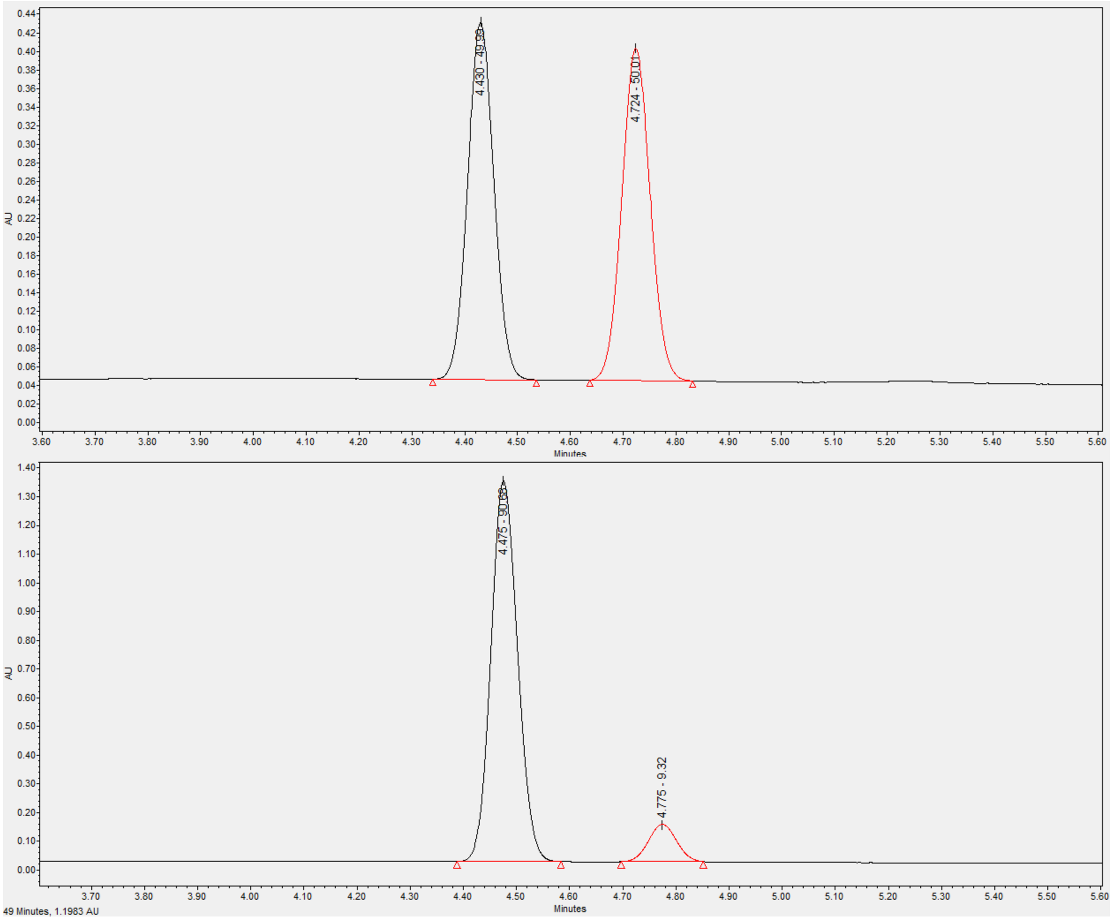

Supplementary Figure 15: ^{1}H, ^{13}C NMR and SFC trace for **2o**

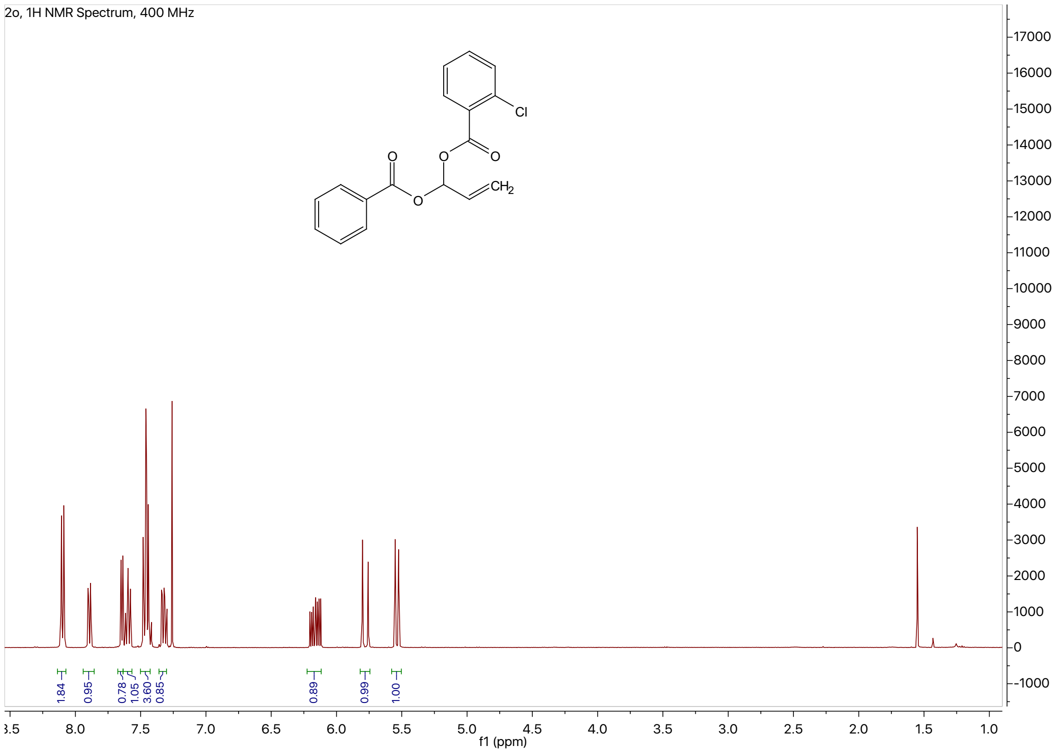

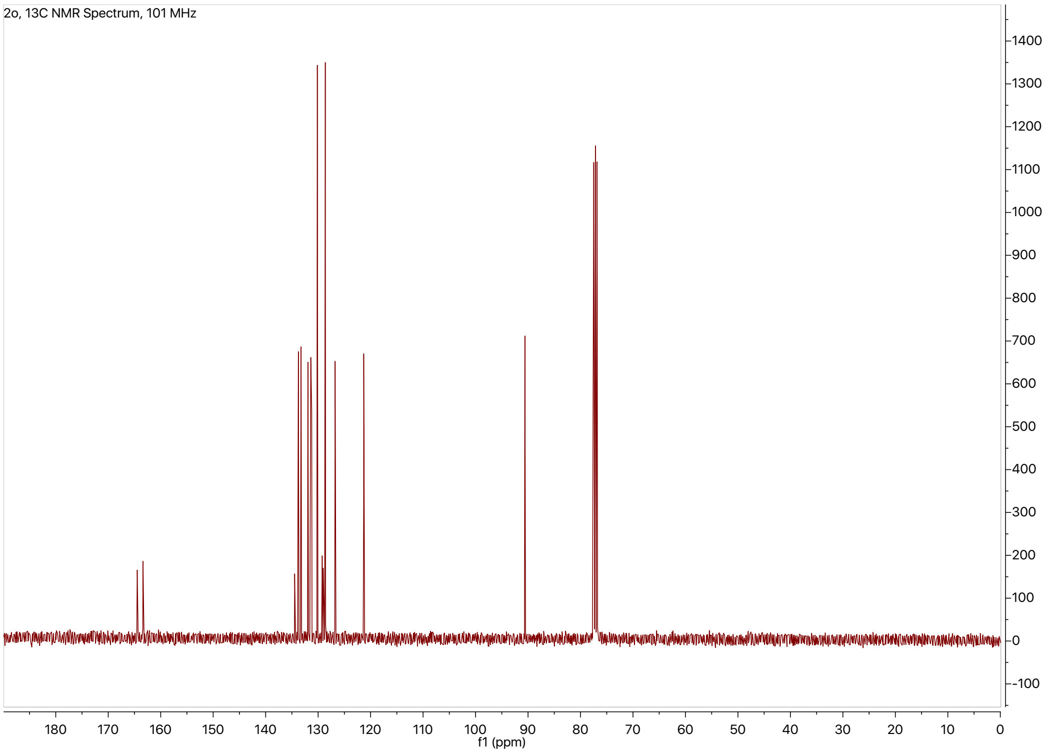
2o, 13C NMR Spectrum, 101 MHz
f1 (ppm)

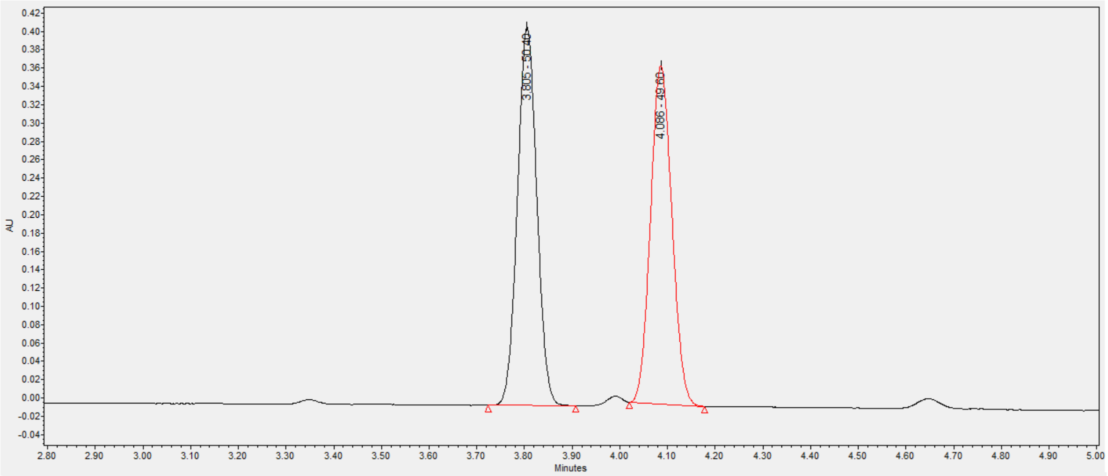
AU
Minutes
3.805 - 50.40
4.086 - 49.60

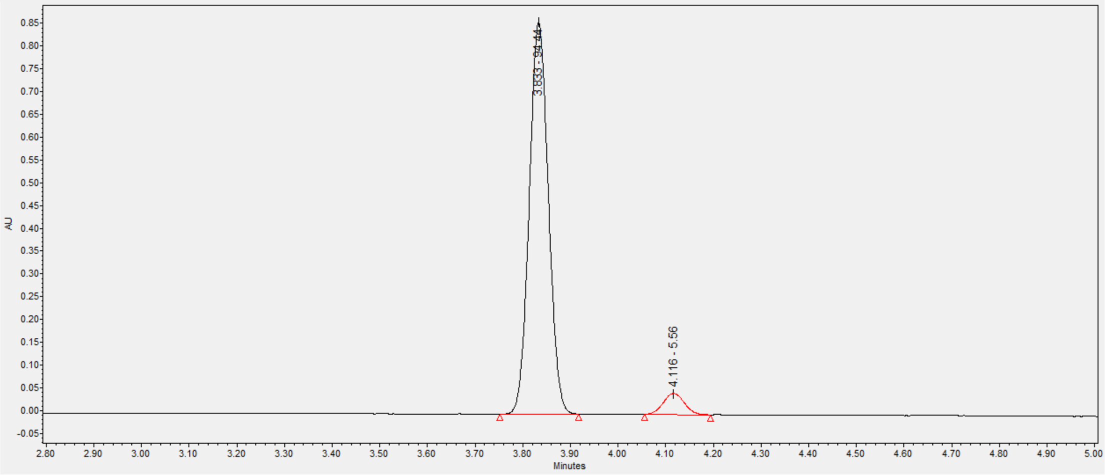
AU
Minutes
3.833 - 94.44
4.116 - 5.56

Supplementary Figure 16: ^{1}H, ^{13}C NMR and SFC trace for **2p**

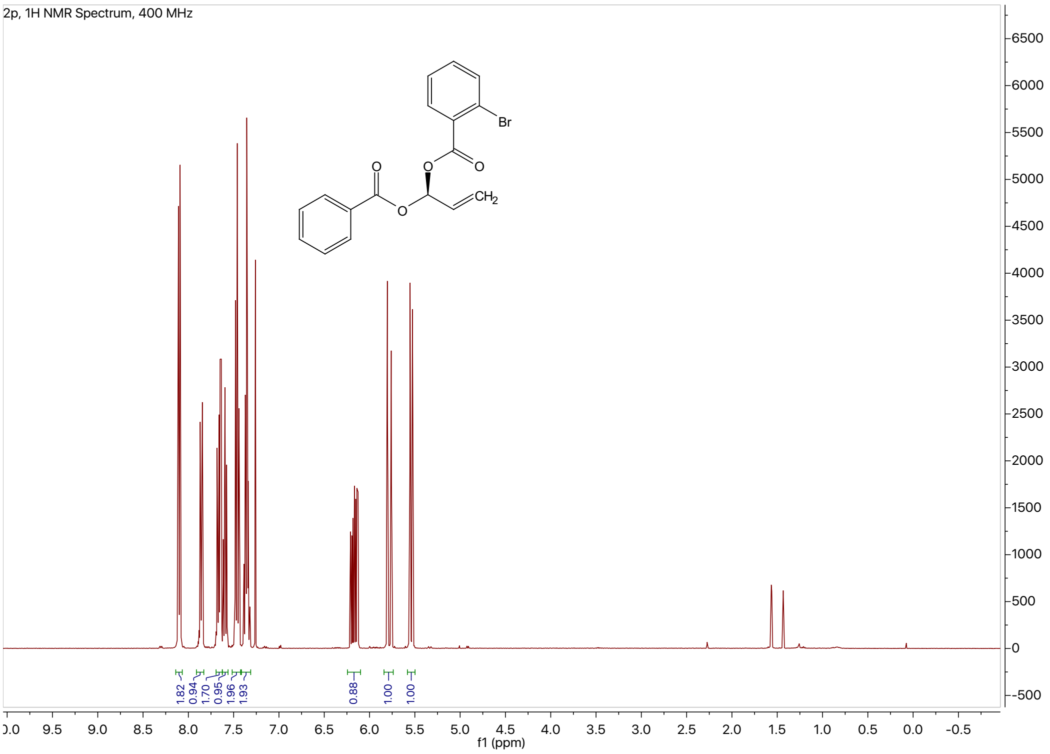

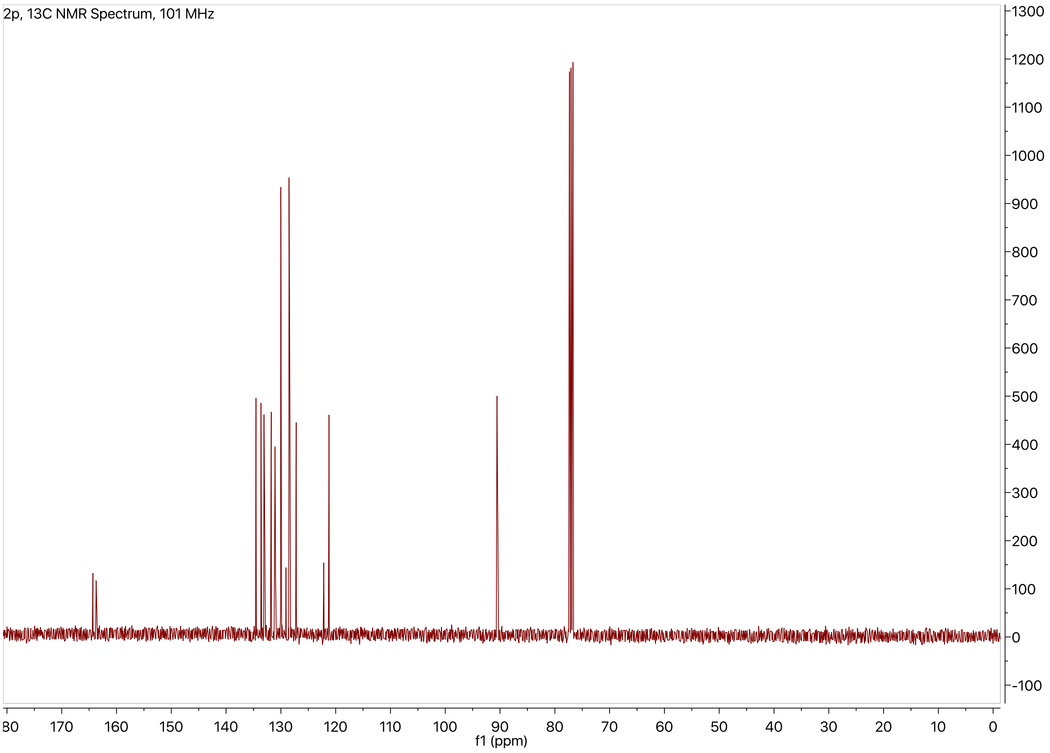

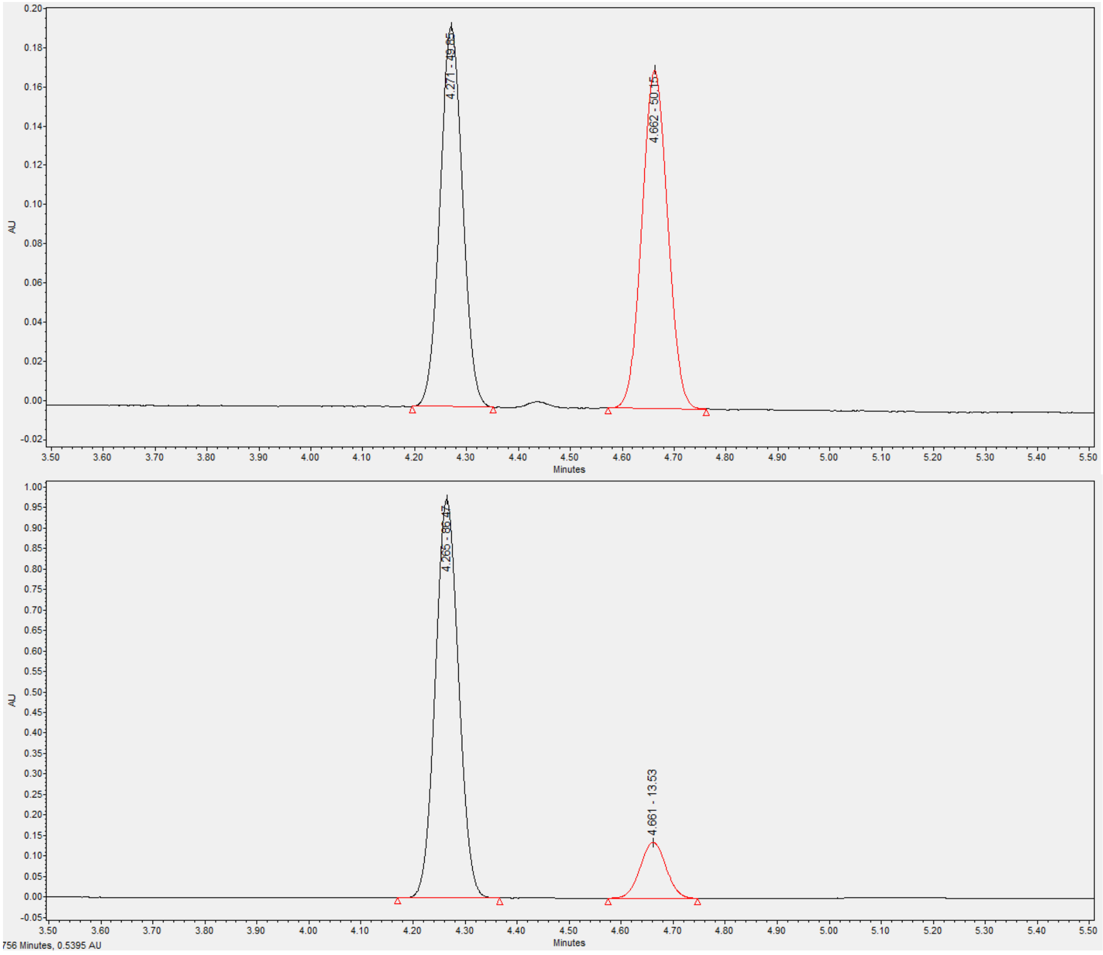

Supplementary Figure 17: ^{1}H, ^{13}C NMR and SFC trace for **2q**

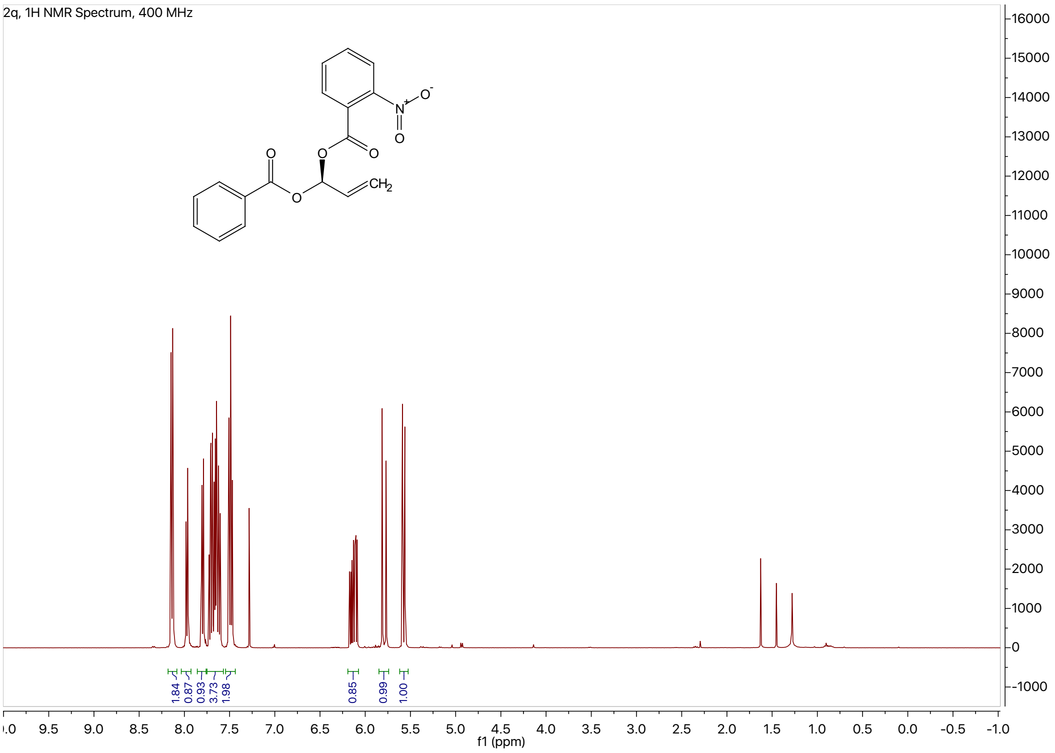

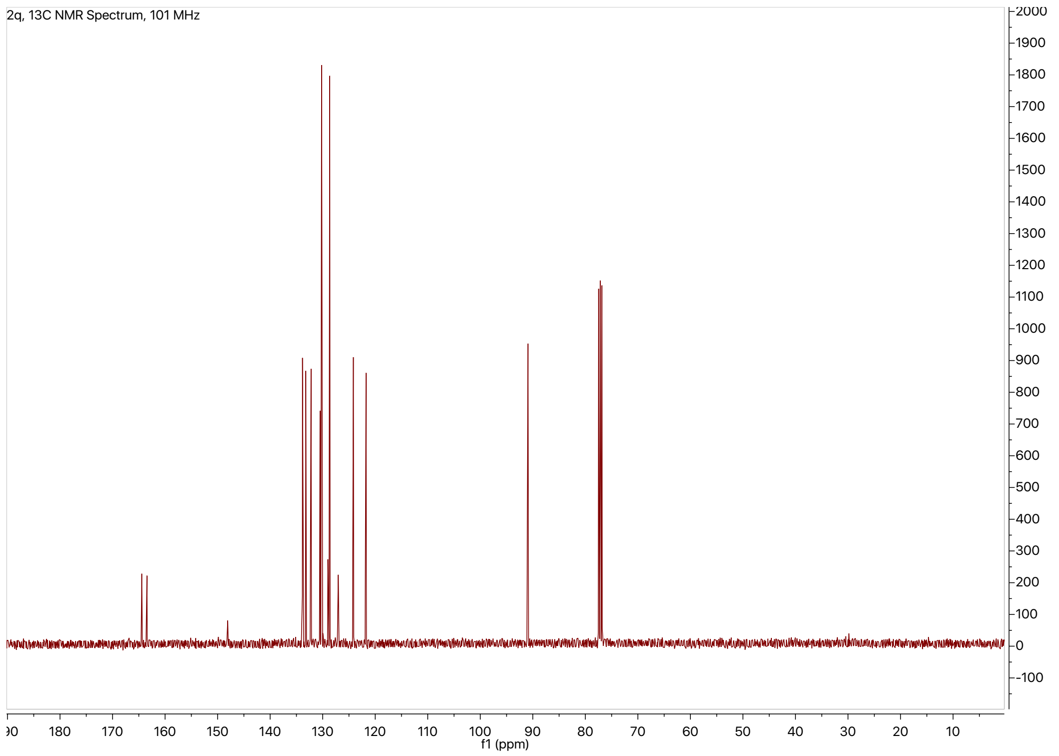
2q, 13C NMR Spectrum, 101 MHz
f1 (ppm)

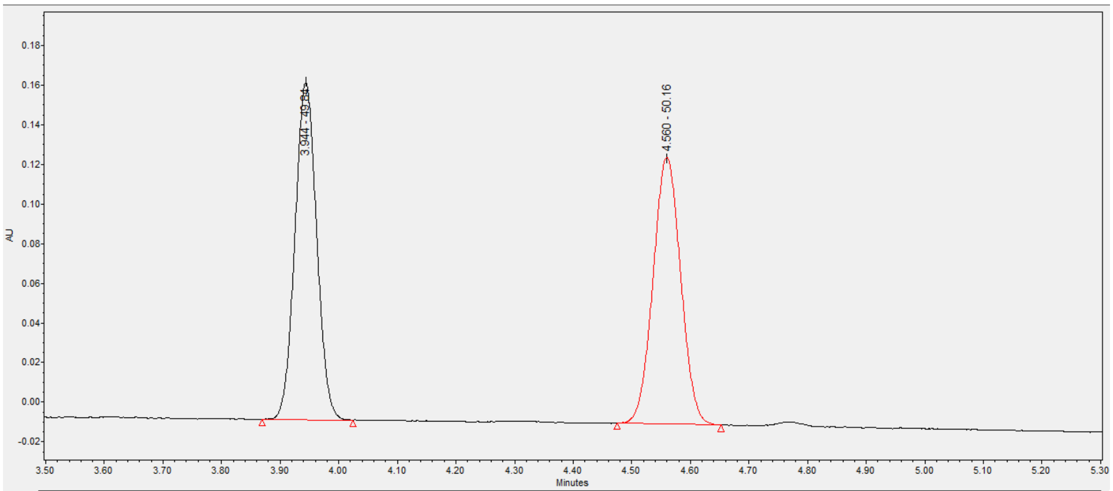
4.560 - 50.16
AU
Minutes

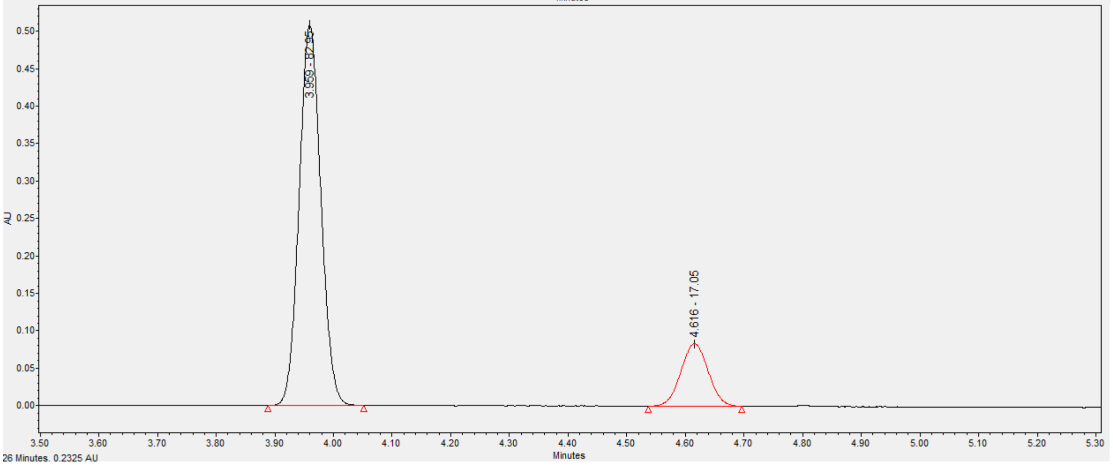
4.616 - 17.05
AU
Minutes

Supplementary Figure 18: ^{1}H, ^{13}C NMR and SFC trace for **2r**

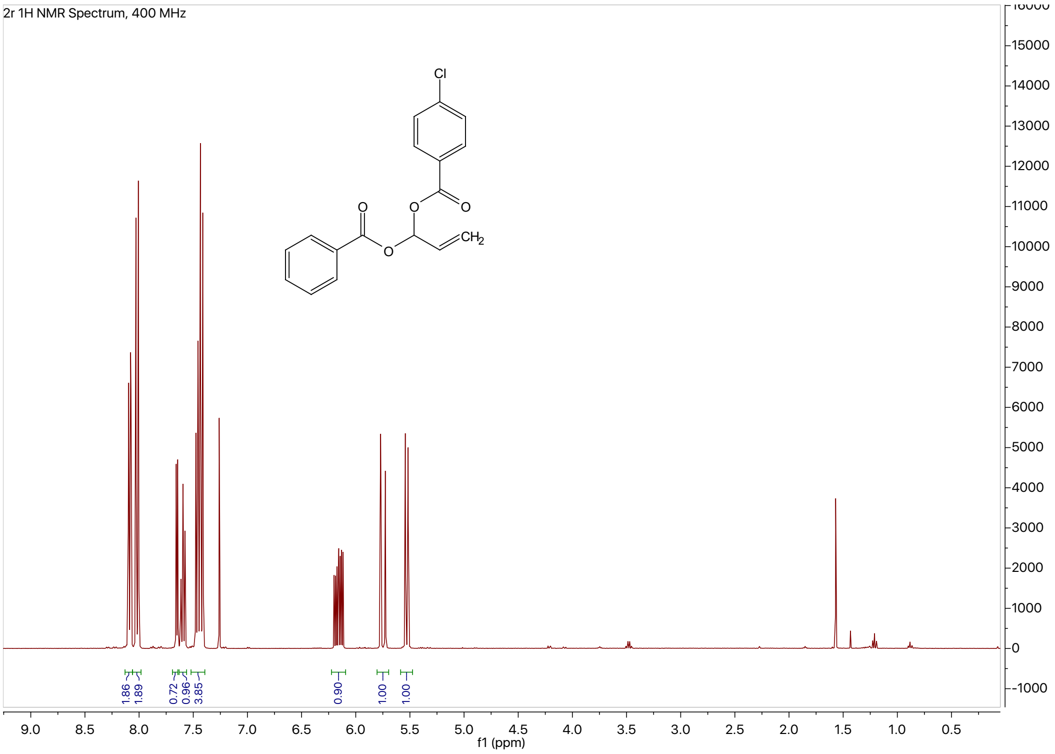

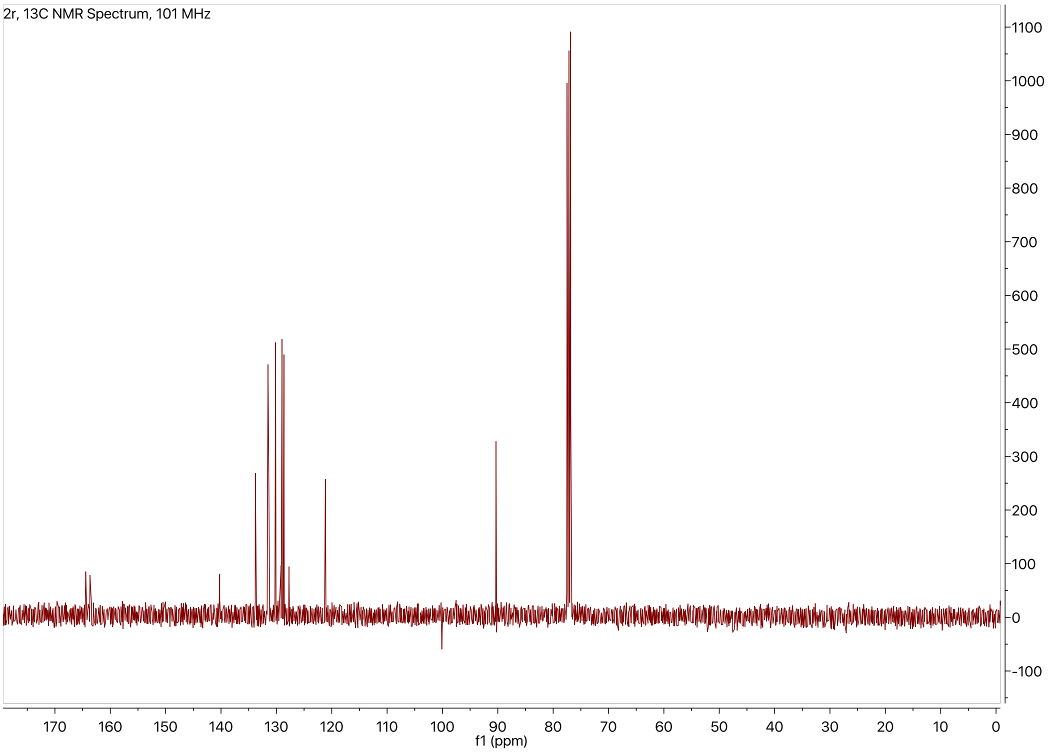

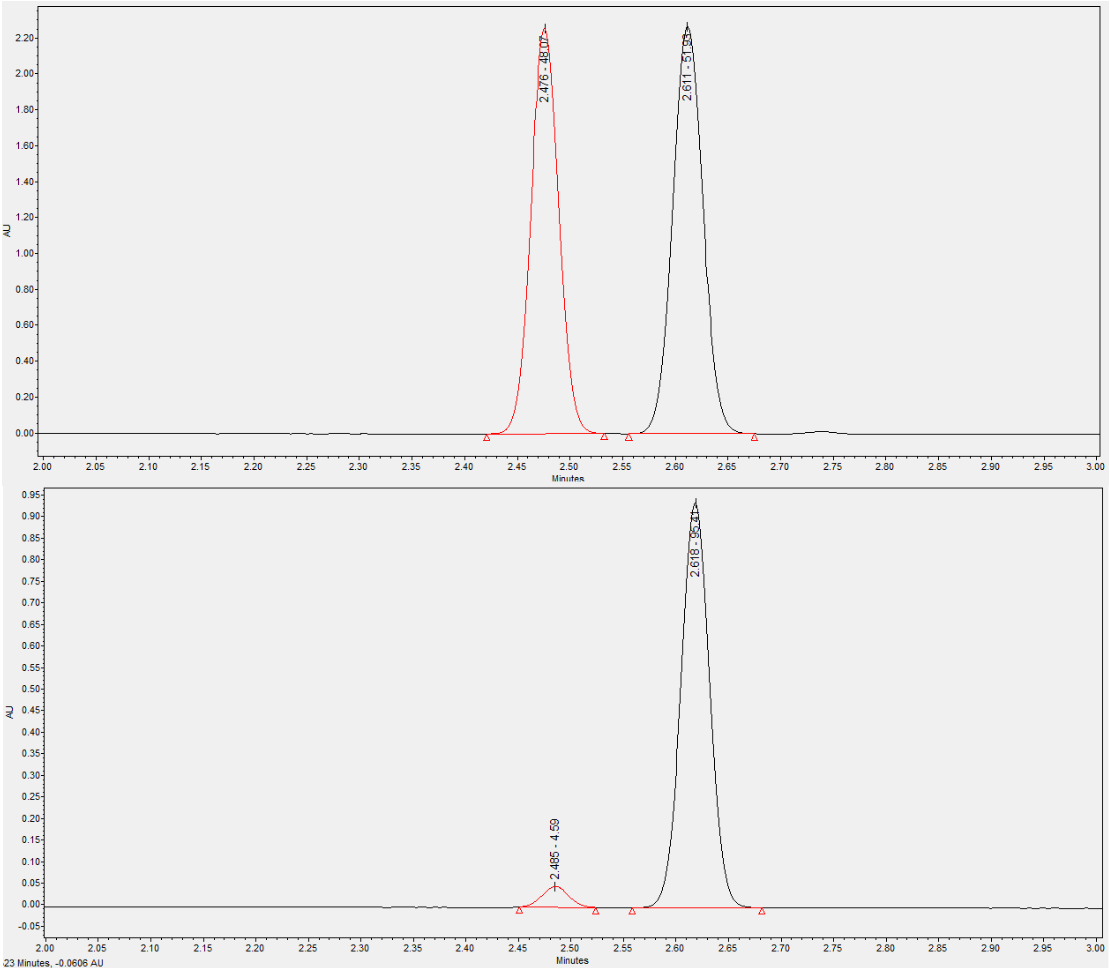

Supplementary Figure 19: ^{1}H, ^{13}C NMR and SFC trace for **2s**

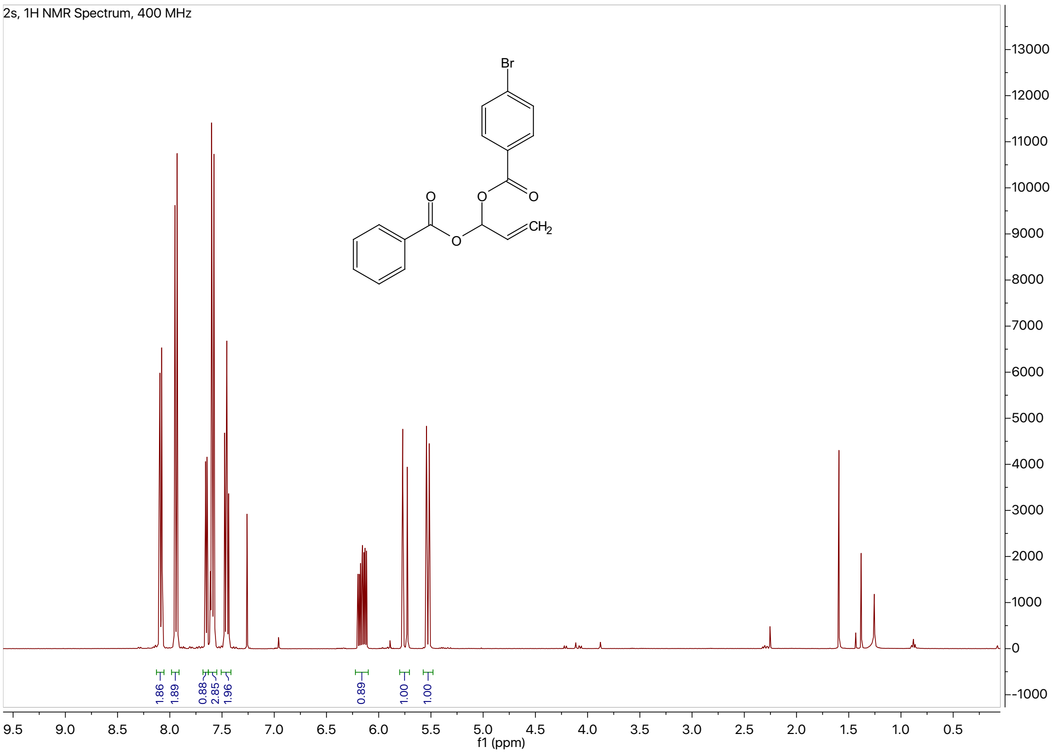

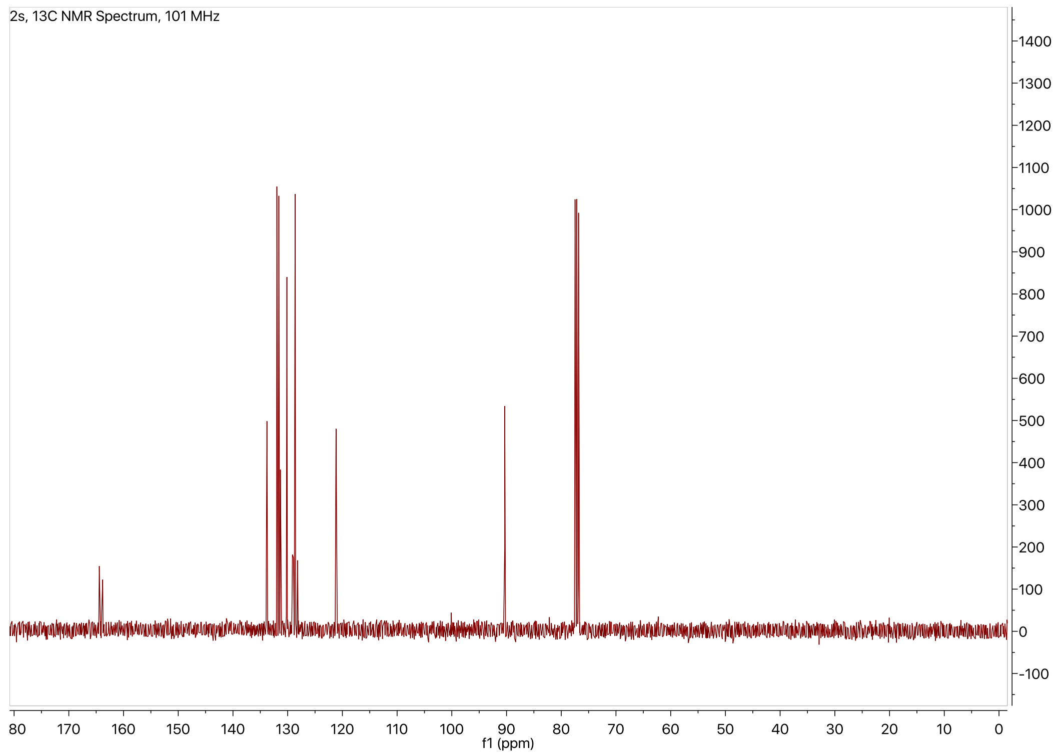

2s, 13C NMR Spectrum, 101 MHz
f1 (ppm)

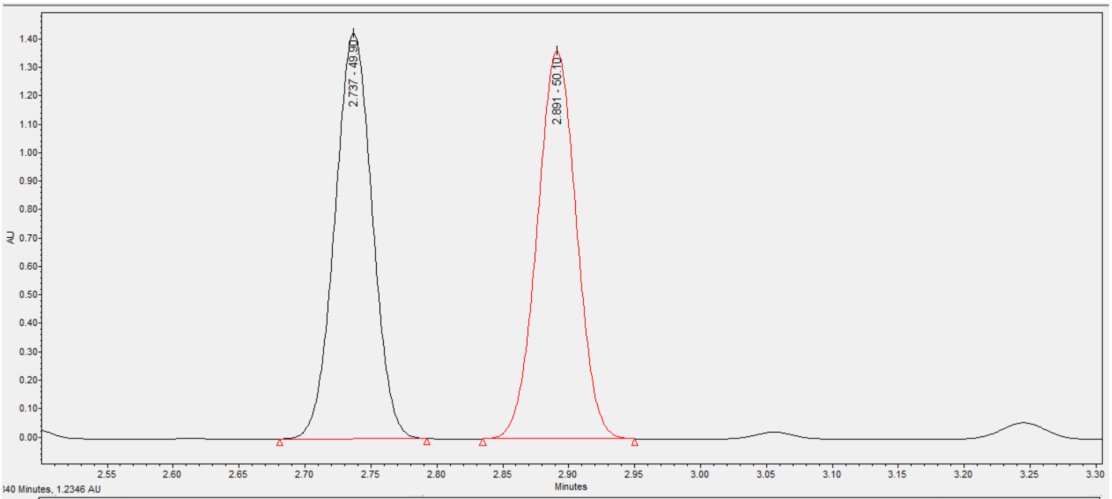

2.737 - 49.90
2.891 - 50.10
AU
Minutes

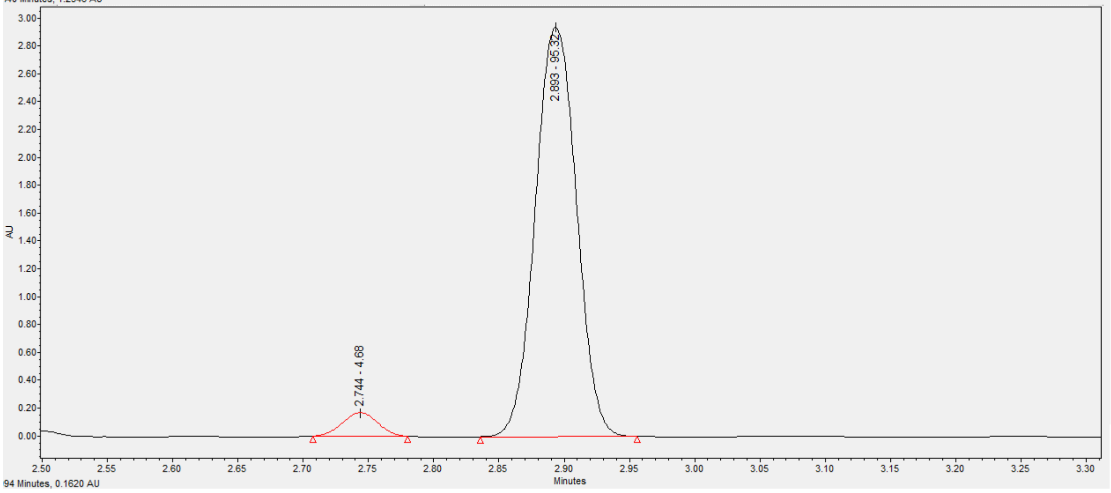

2.893 - 95.32
2.744 - 4.68
AU
Minutes

Supplementary Figure 20: ^{1}H, ^{13}C, ^{19}F NMR and SFC trace for **2t**

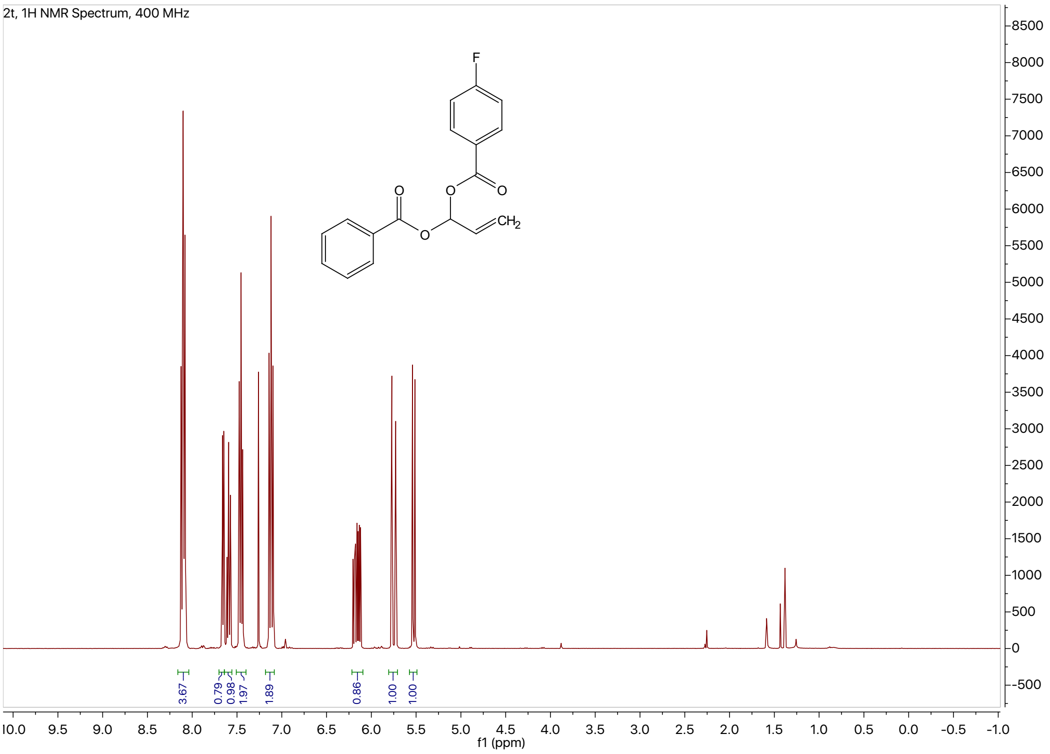

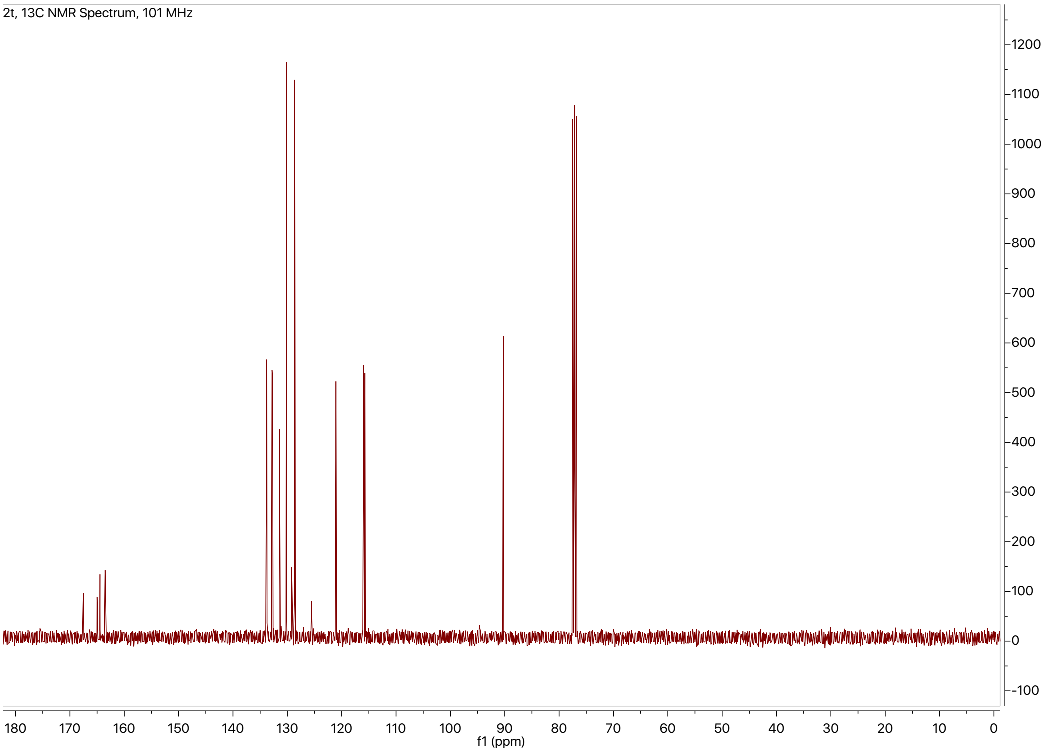

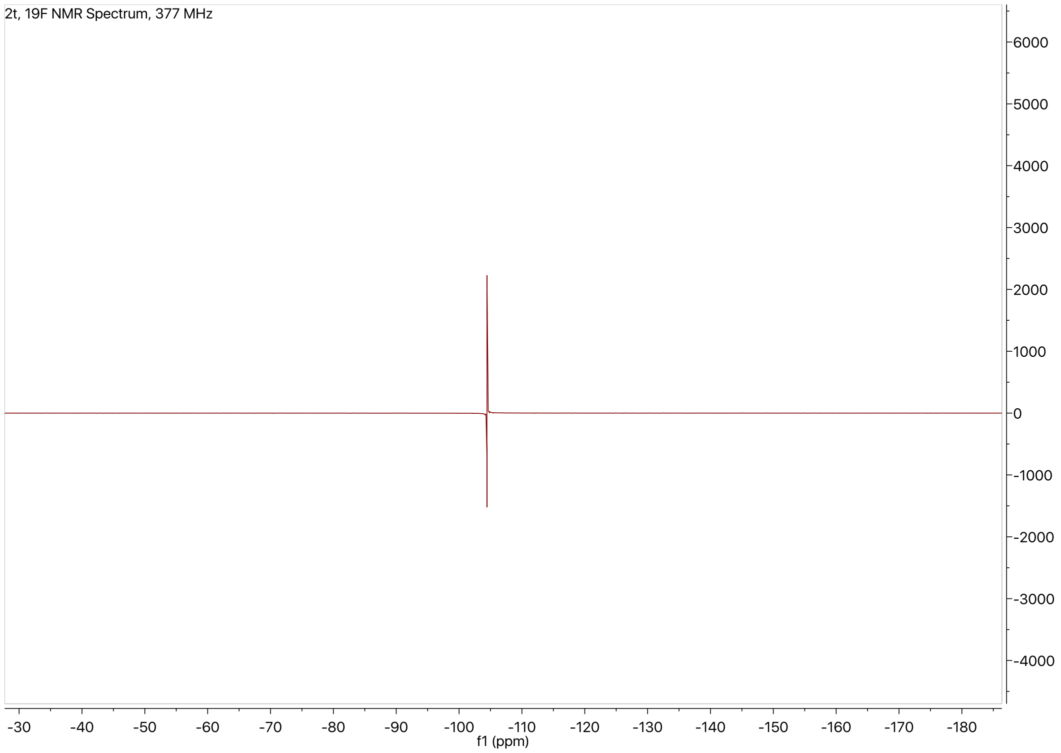
2t, 19F NMR Spectrum, 377 MHz
f1 (ppm)

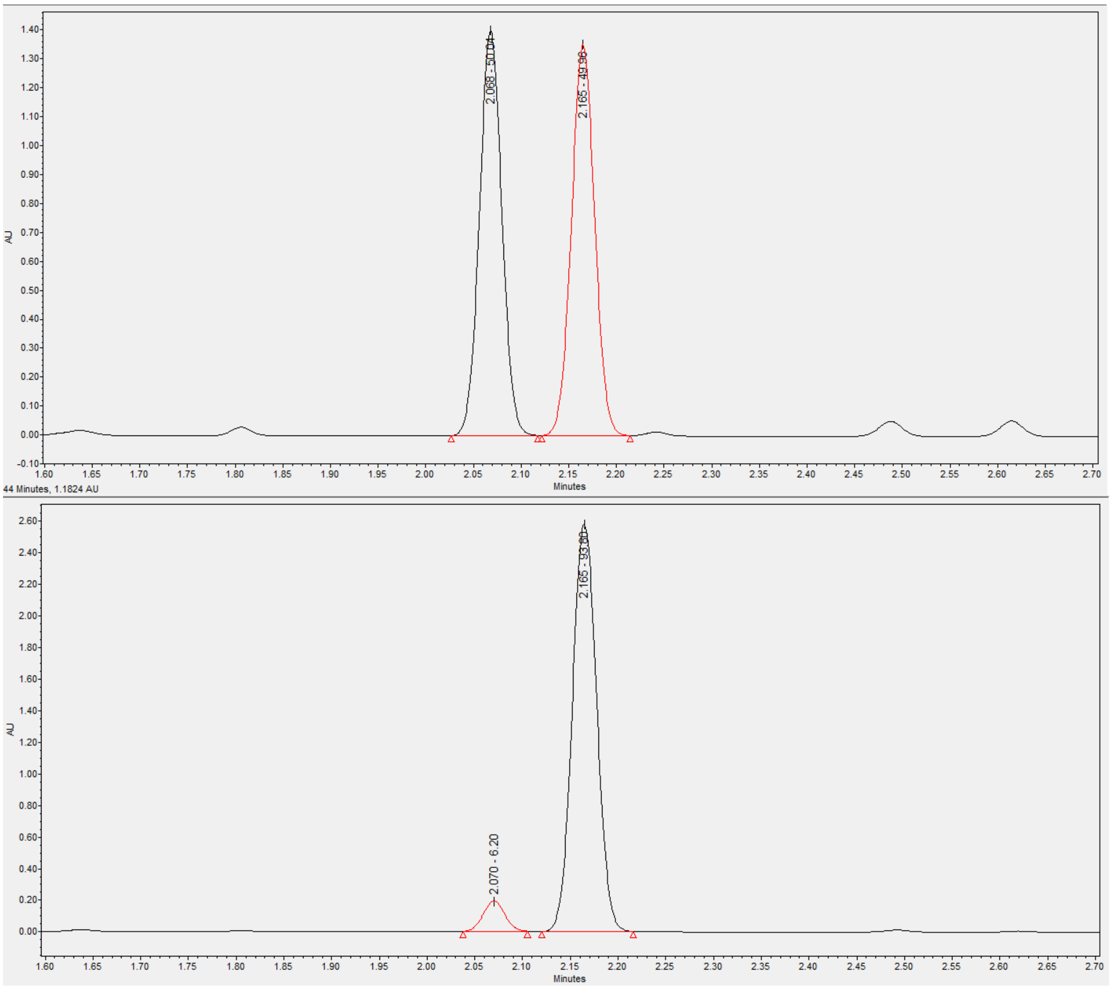
2.068 - 50.04
2.165 - 49.96
AU
Minutes
44 Minutes, 1.1824 AU
2.070 - 6.20
2.165 - 93.80
AU
Minutes

Supplementary Figure 21: ^{1}H, ^{13}C NMR and SFC trace for **2u**

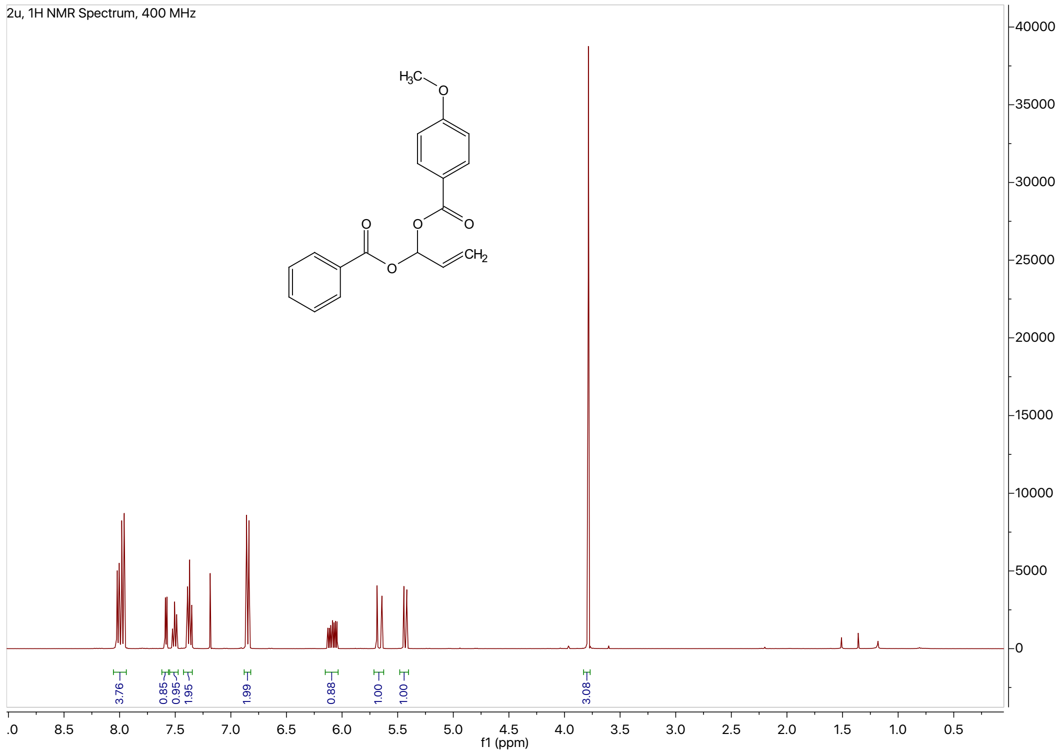

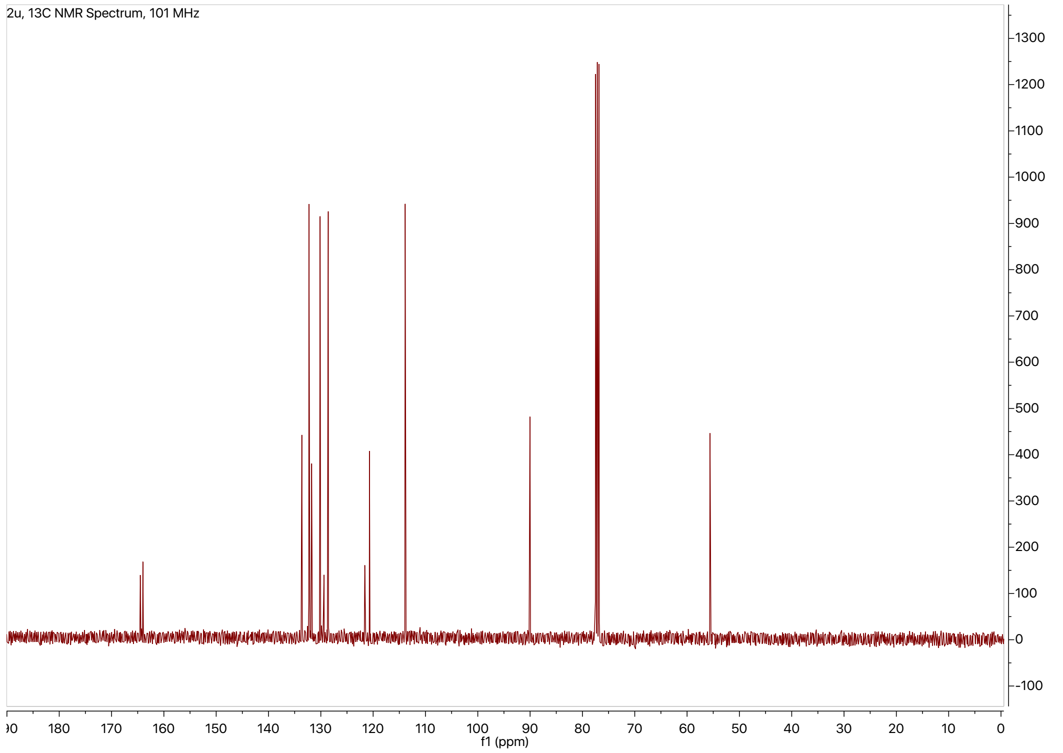

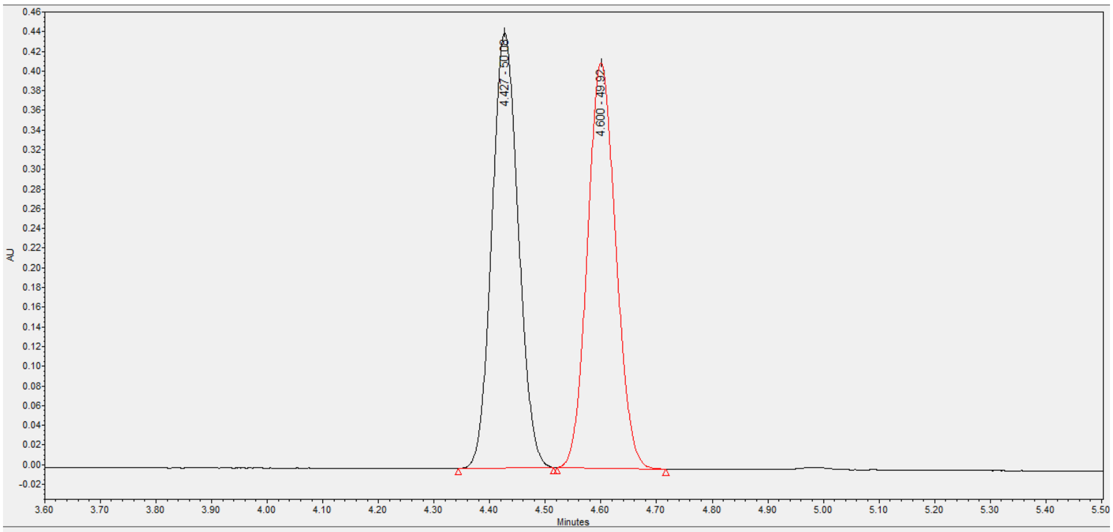
4.427 - 50.08
4.600 - 49.92
AU
Minutes

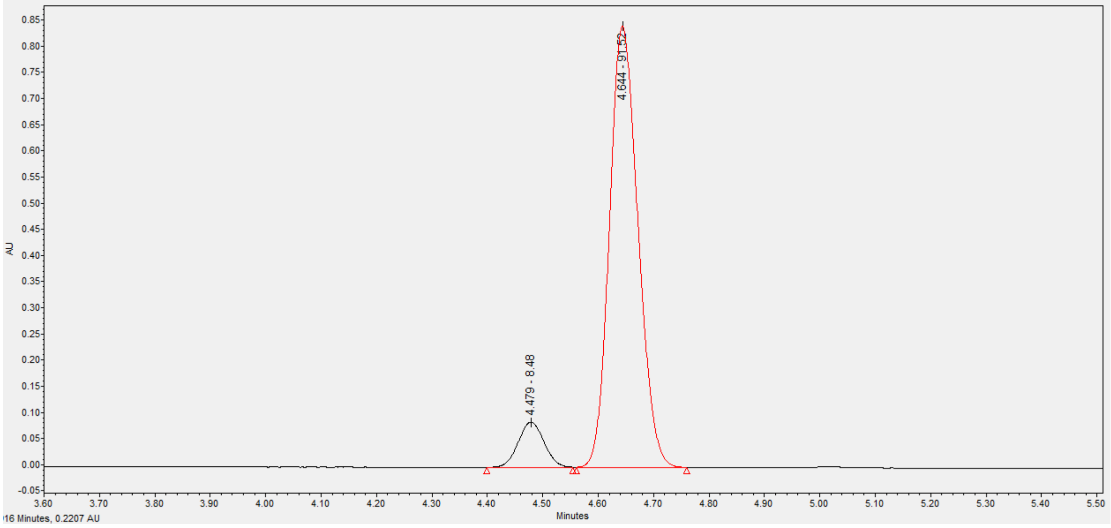
4.479 - 8.48
4.644 - 91.52
AU
Minutes
16 Minutes, 0.2207 AU

Supplementary Figure 22: ^{1}H, ^{13}C, ^{19}F NMR and SFC trace for **2v**

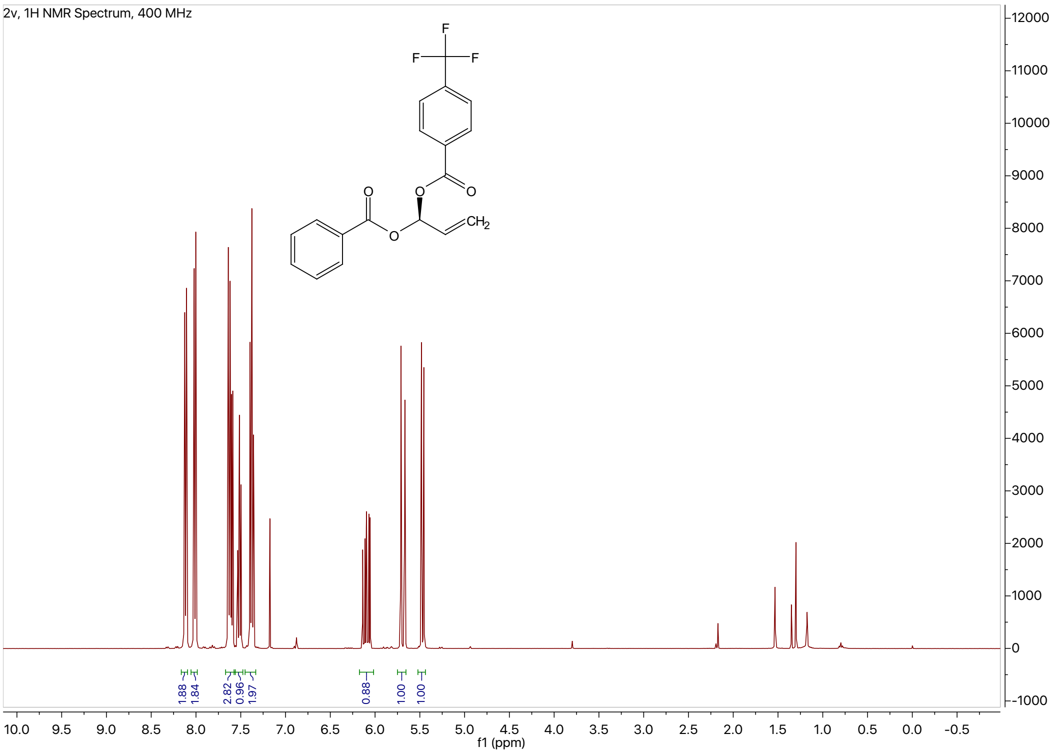

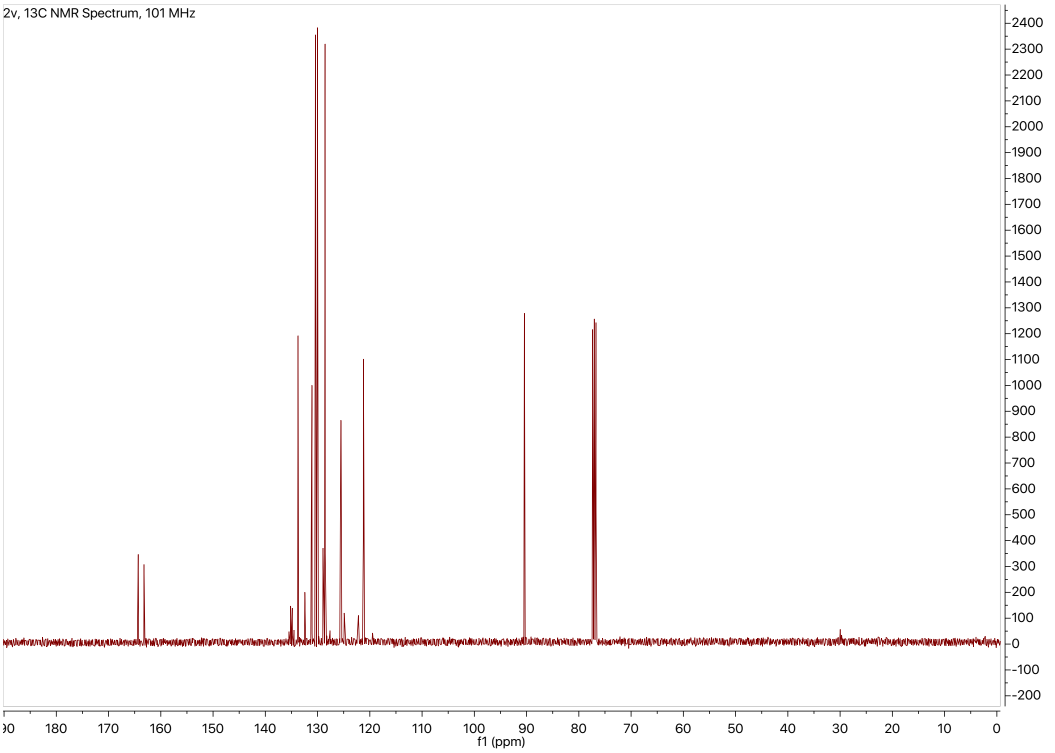

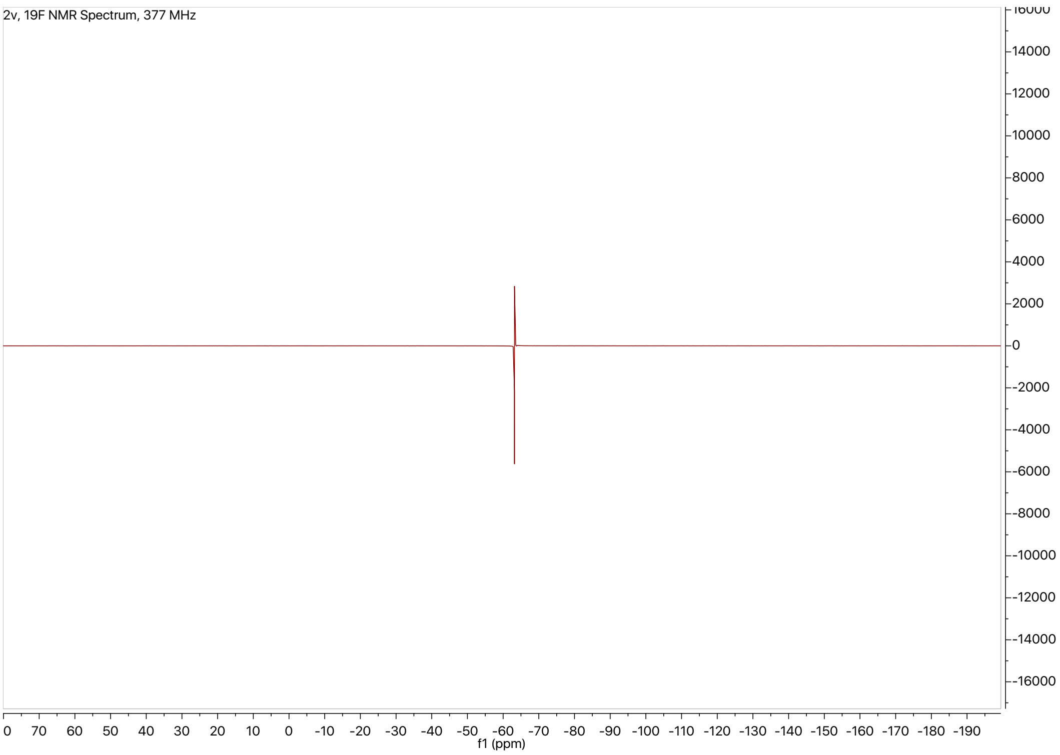
2v, 19F NMR Spectrum, 377 MHz
f1 (ppm)

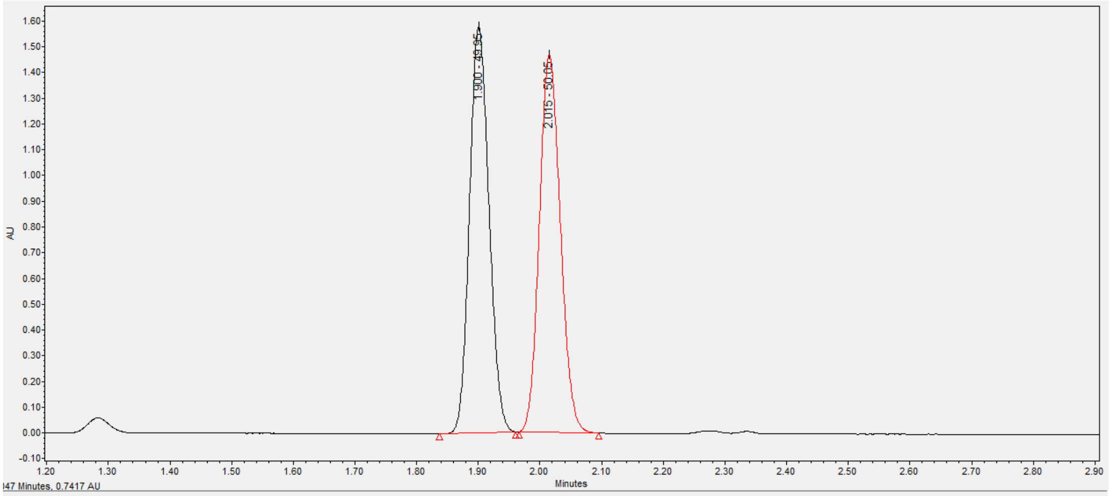
1.900 - 49.95
2.015 - 50.05
AU
Minutes

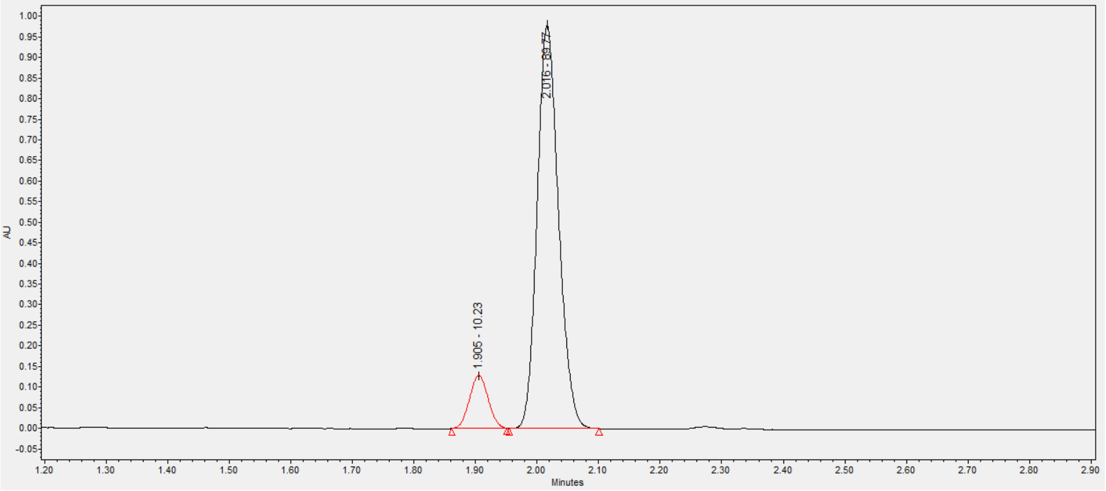
1.905 - 10.23
2.016 - 89.77
AU
Minutes

Supplementary Figure 23: ^{1}H, ^{13}C NMR and SFC trace for **2w**

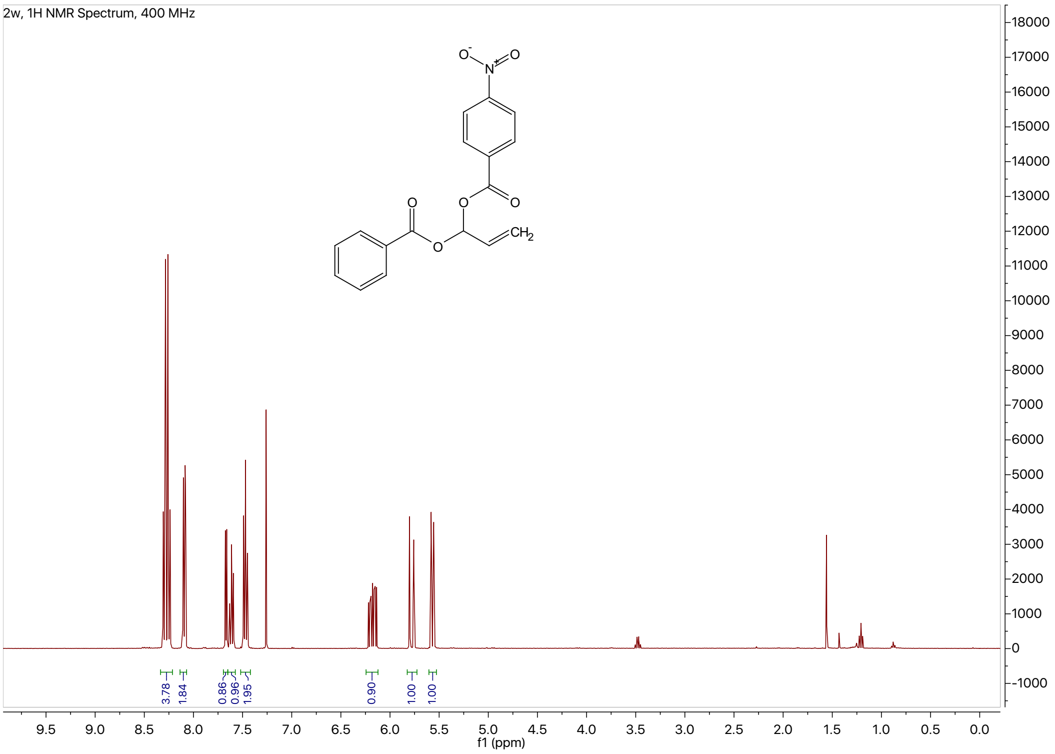

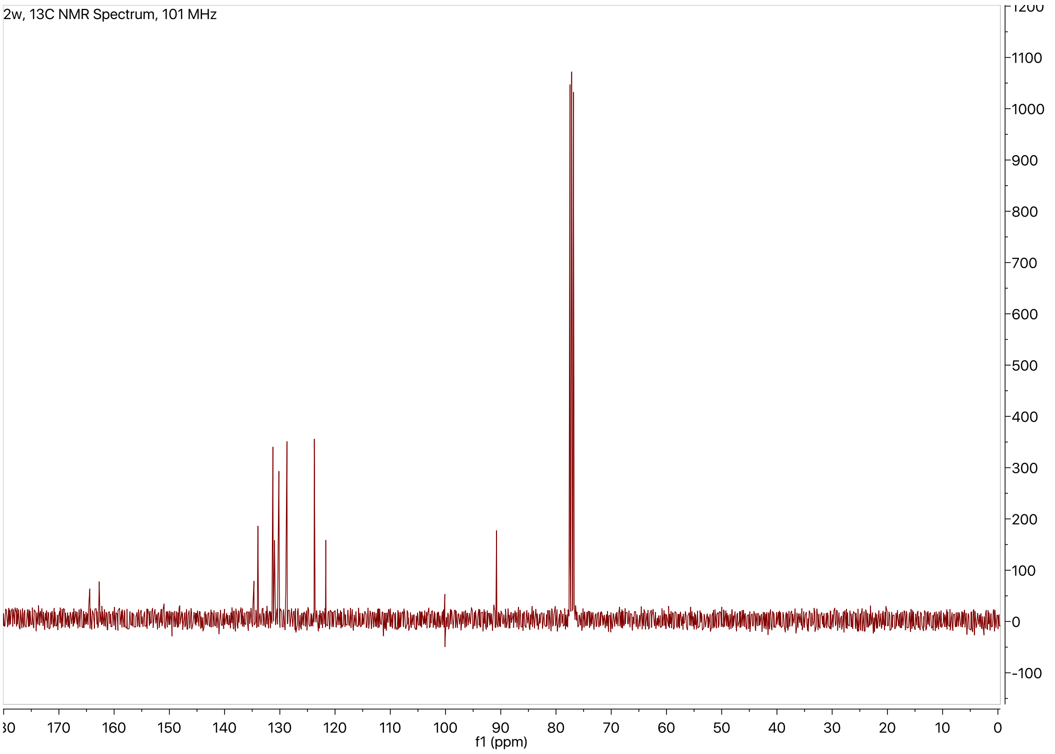

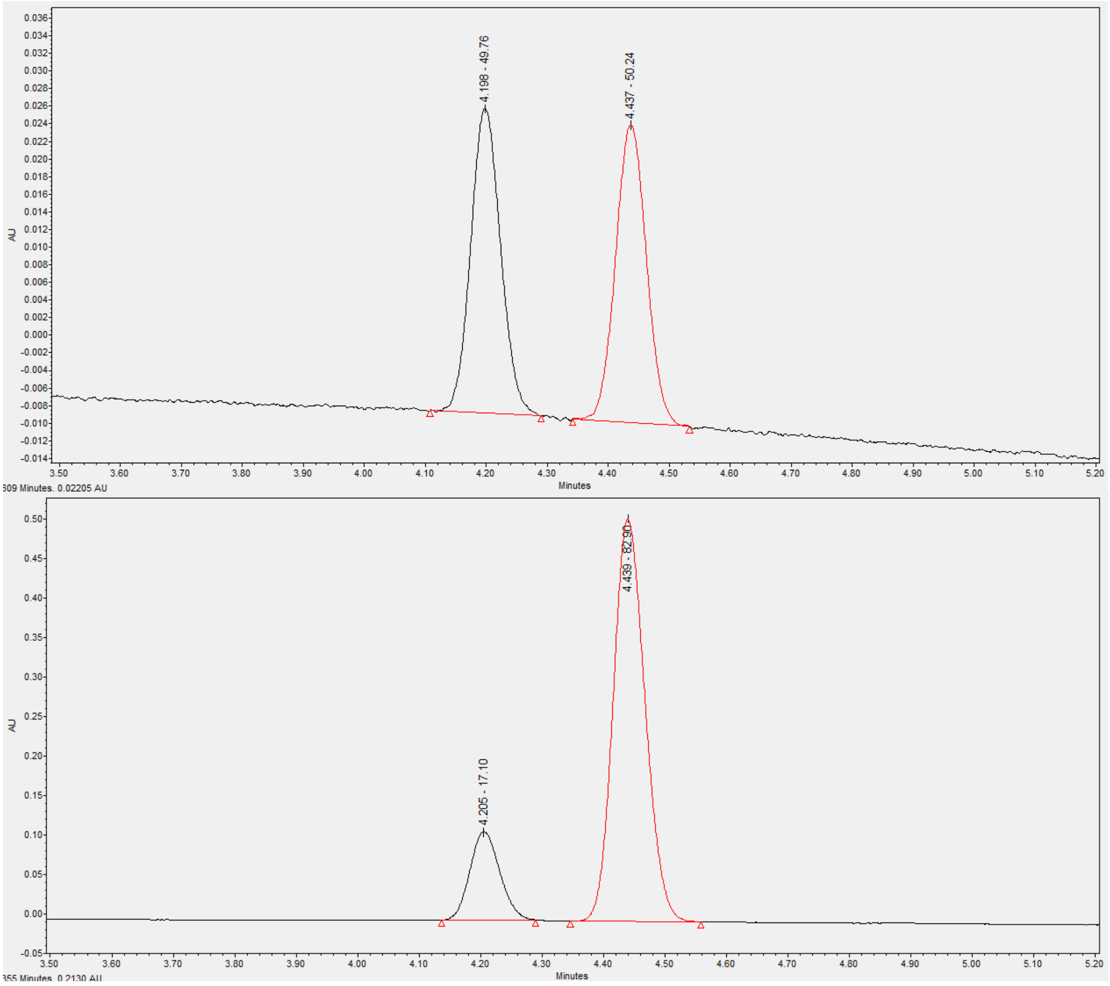

Supplementary Figure 24: ^{1}H, ^{13}C NMR and SFC trace for **2xa**

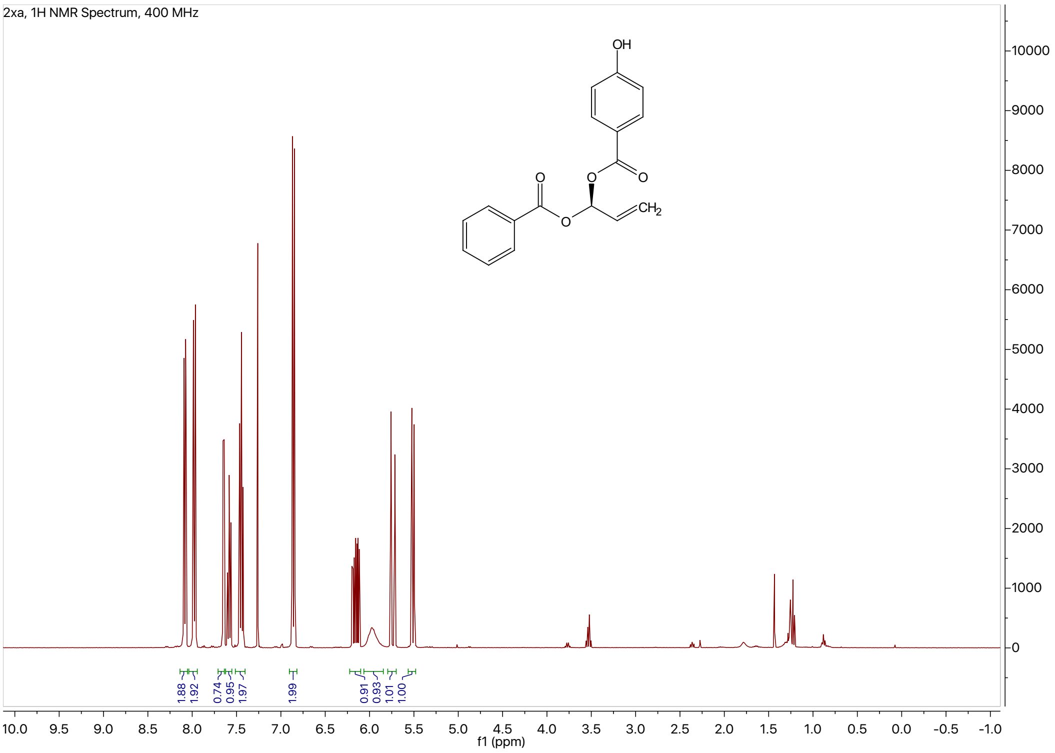

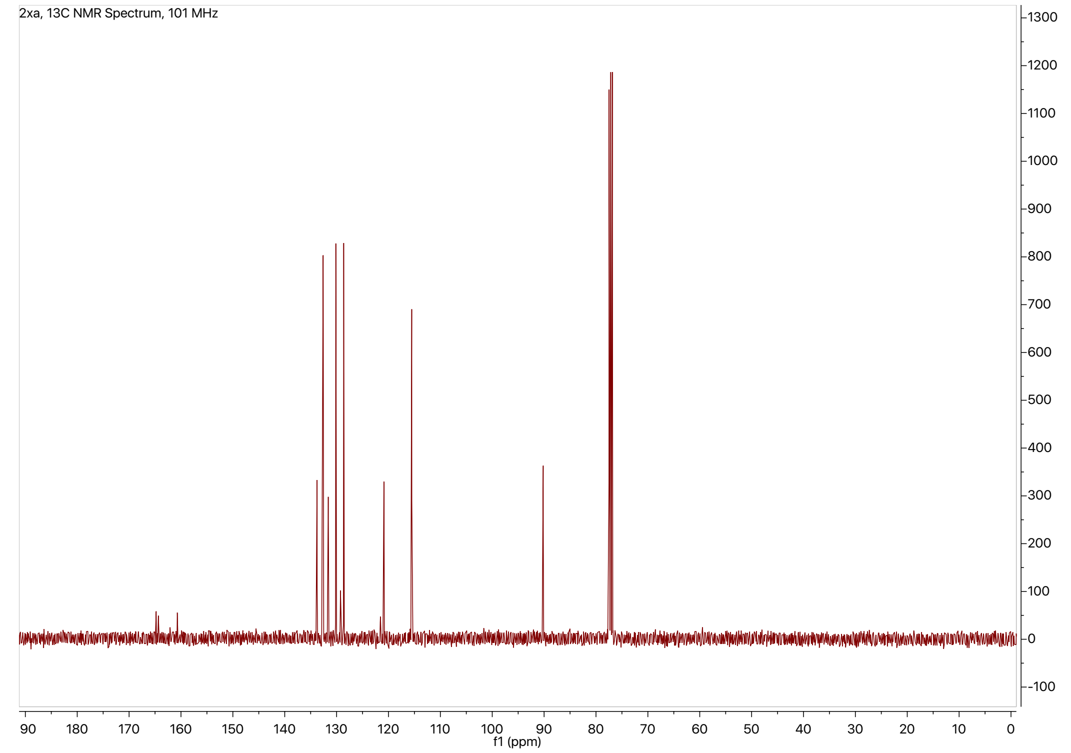
2xa, 13C NMR Spectrum, 101 MHz
f1 (ppm)

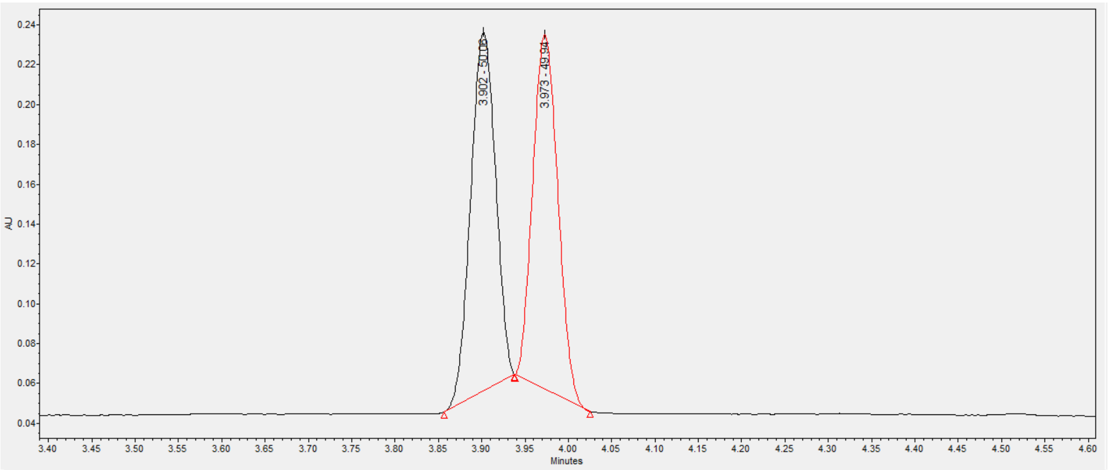
3.902 - 50.06
3.973 - 49.94
AU
Minutes

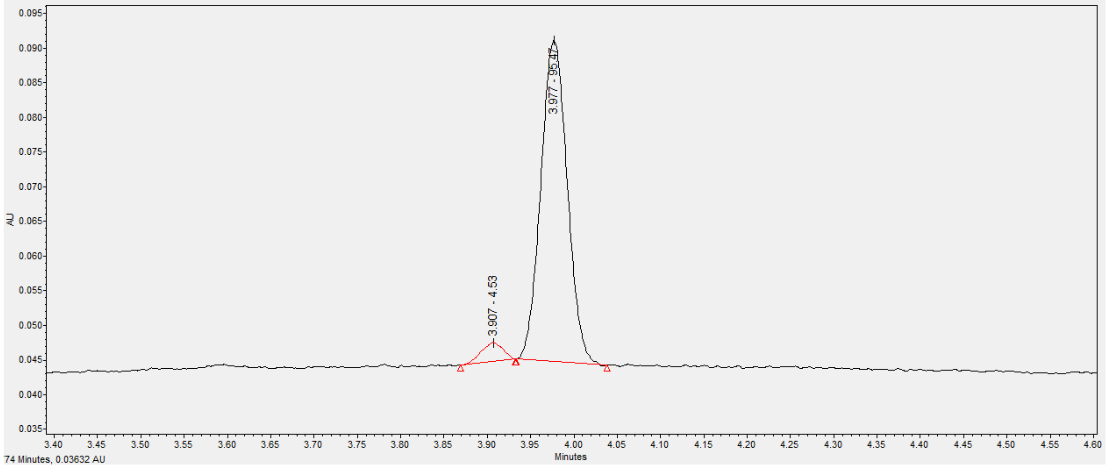
3.907 - 4.53
3.977 - 95.47
AU
Minutes
74 Minutes, 0.03632 AU

Supplementary Figure 25: ^{1}H, ^{13}C NMR and SFC trace for **2xb**

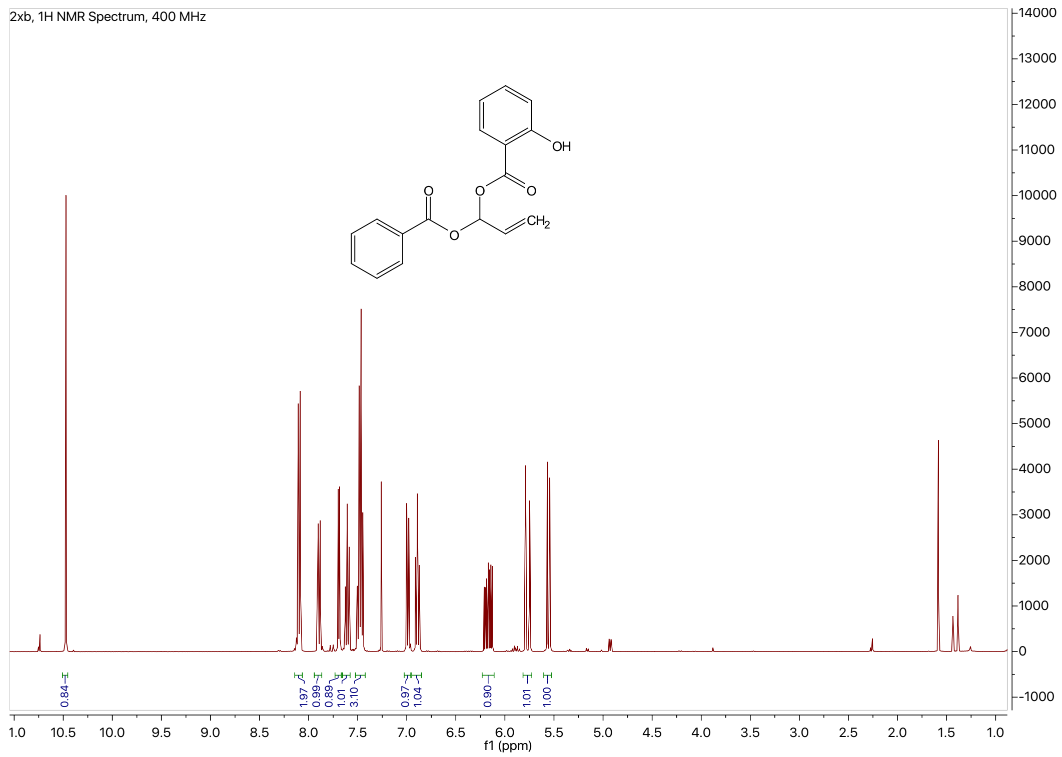

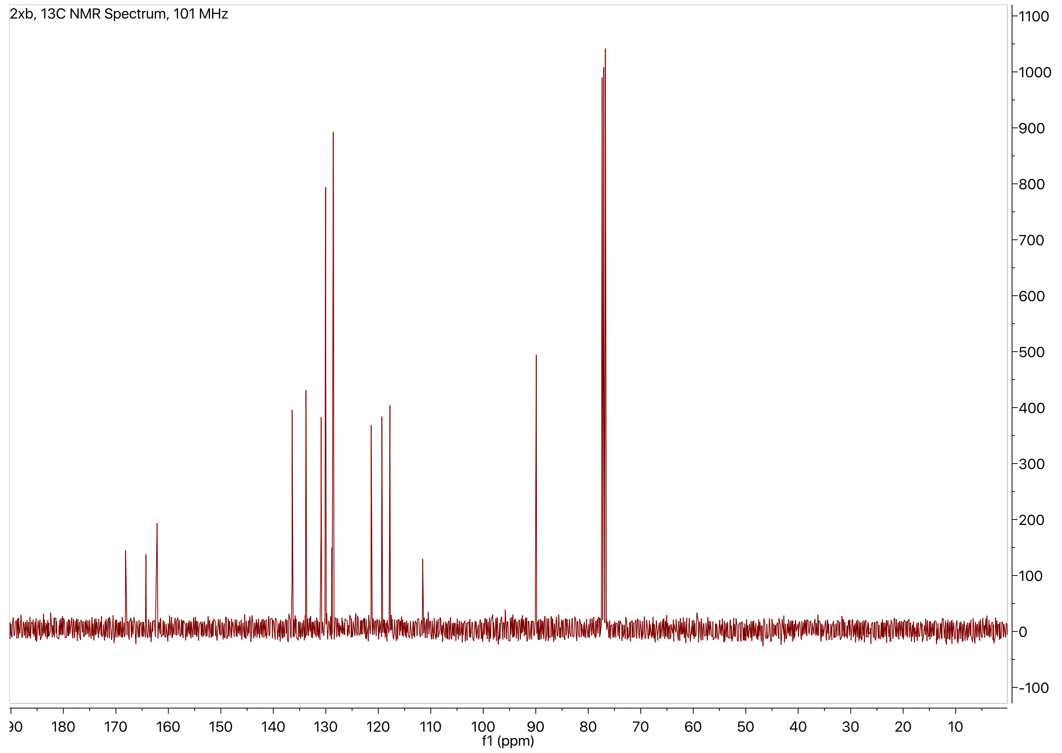

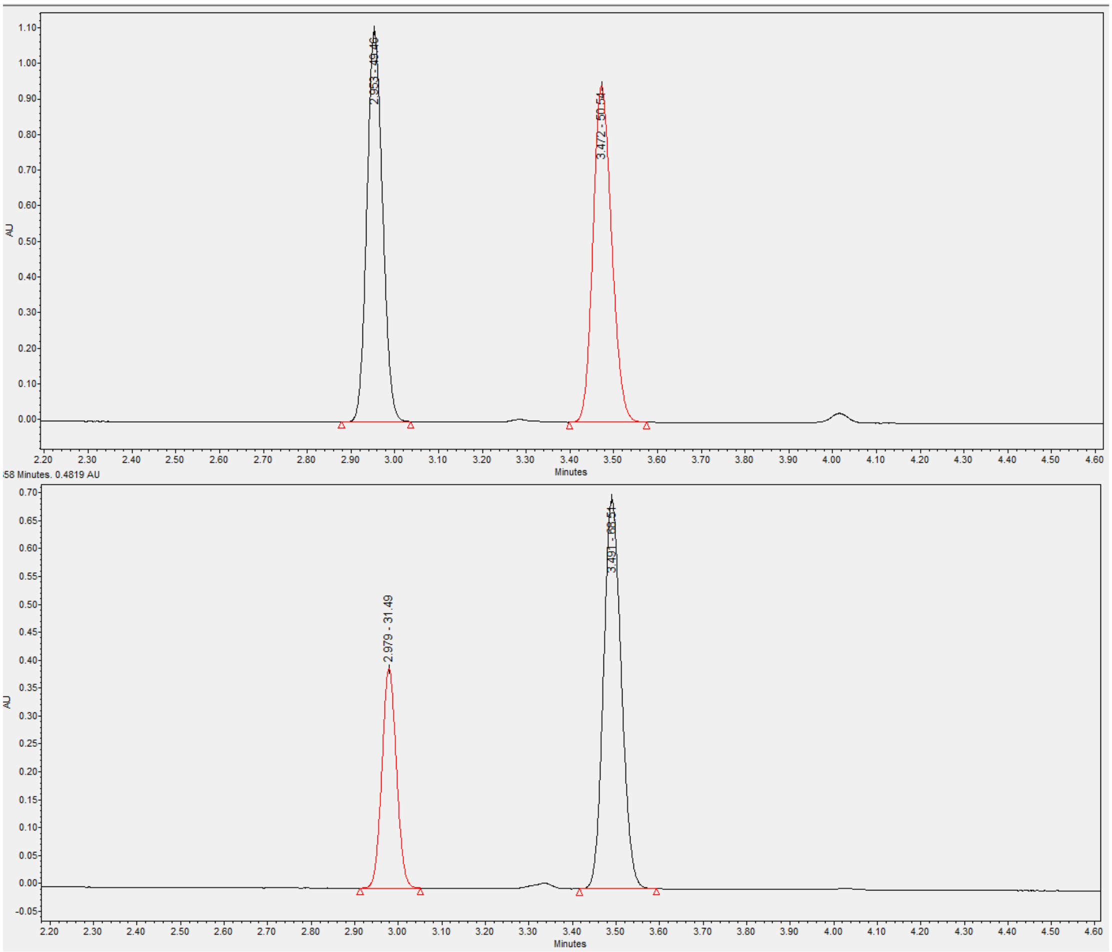

Supplementary Figure 26: ^{1}H, ^{13}C NMR and SFC trace for **2yb**

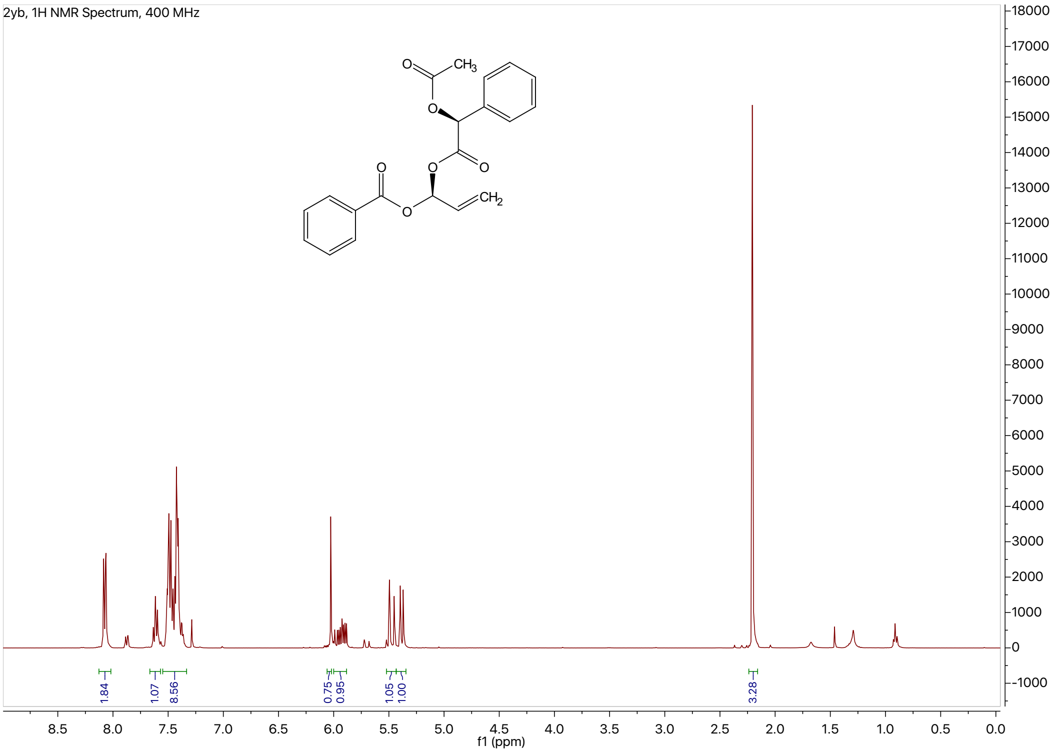

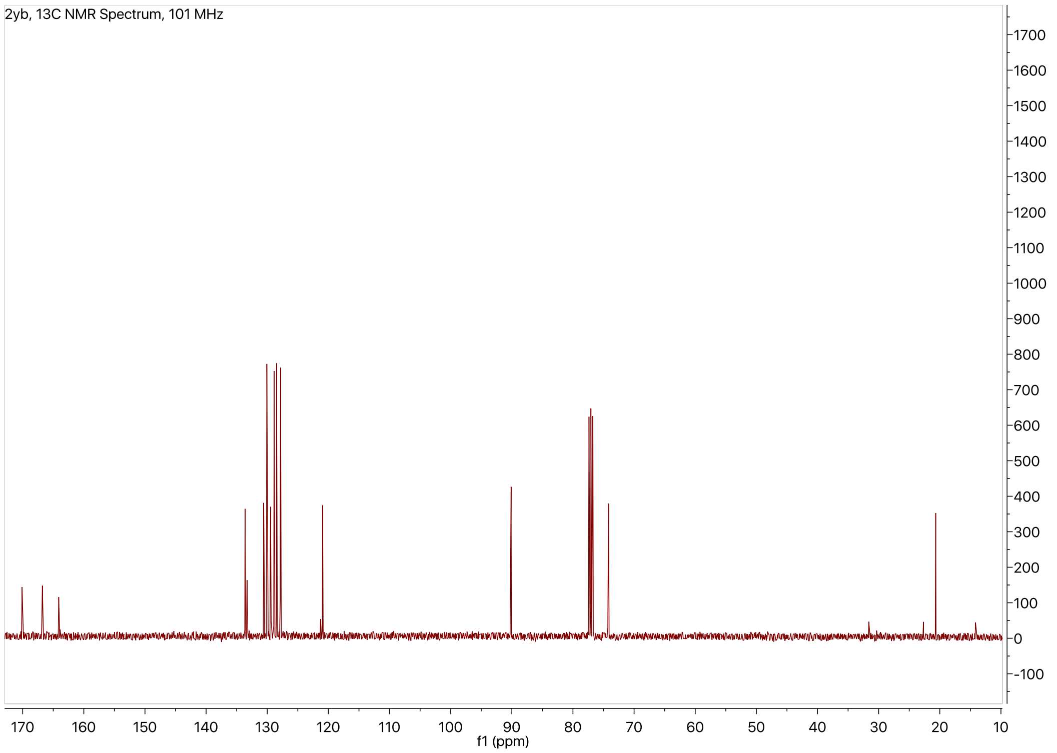
2yb, 13C NMR Spectrum, 101 MHz
f1 (ppm)

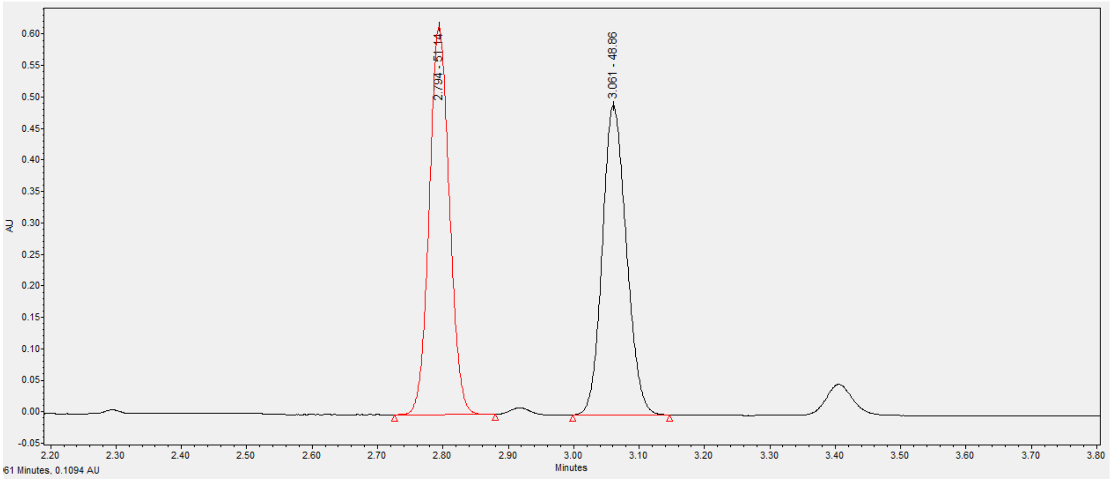
2.794 - 51.14
3.061 - 48.86
AU
Minutes

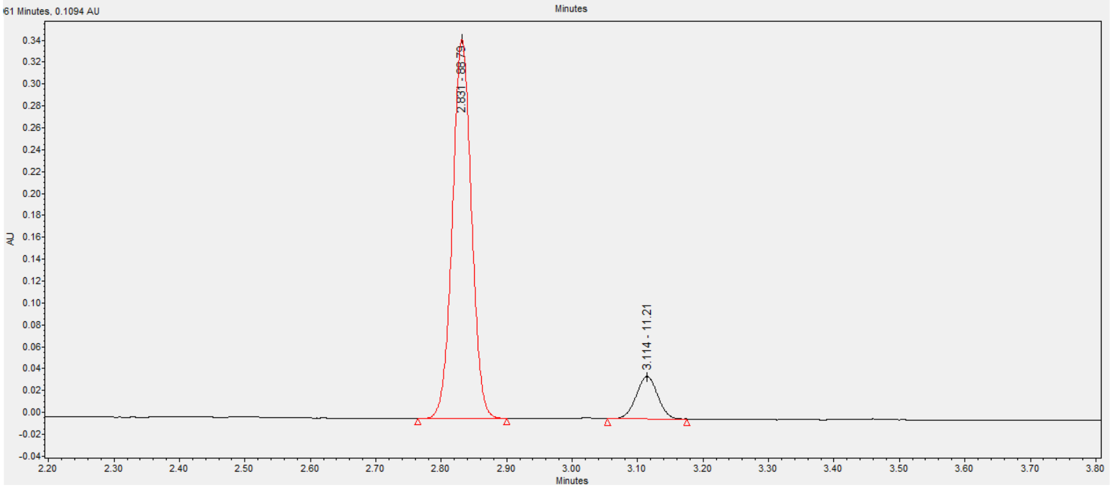
61 Minutes, 0.1094 AU
2.831 - 88.79
3.114 - 11.21
AU
Minutes

Supplementary Figure 27: ^{1}H, ^{13}C NMR, NOESY and SFC trace for **3b**

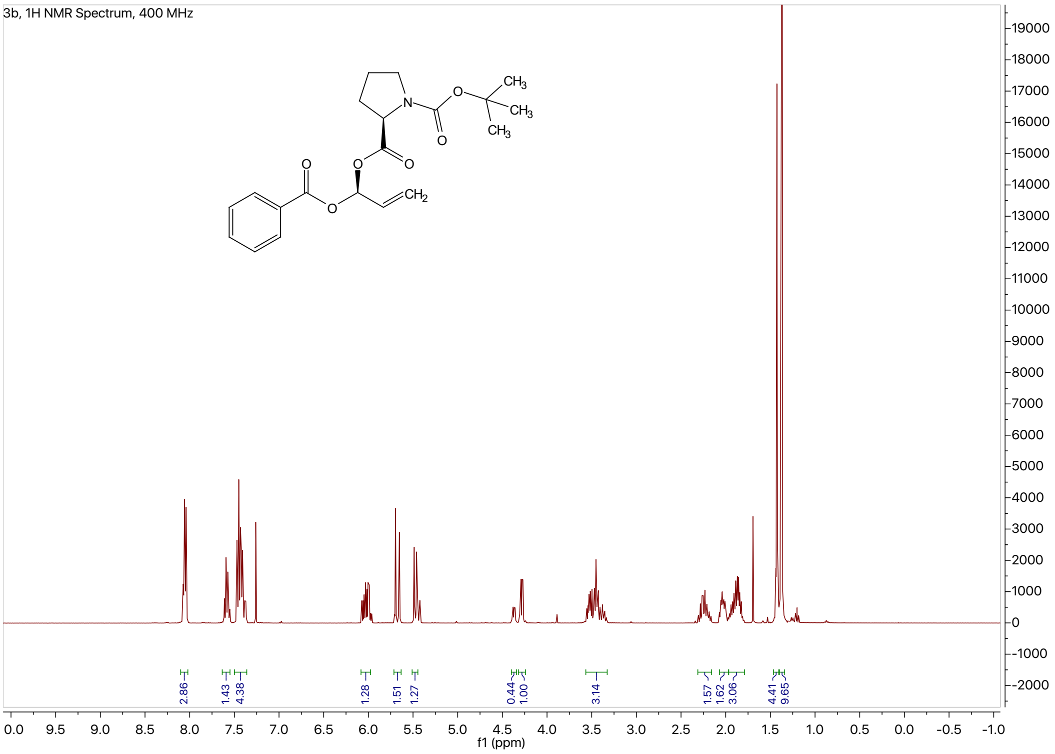

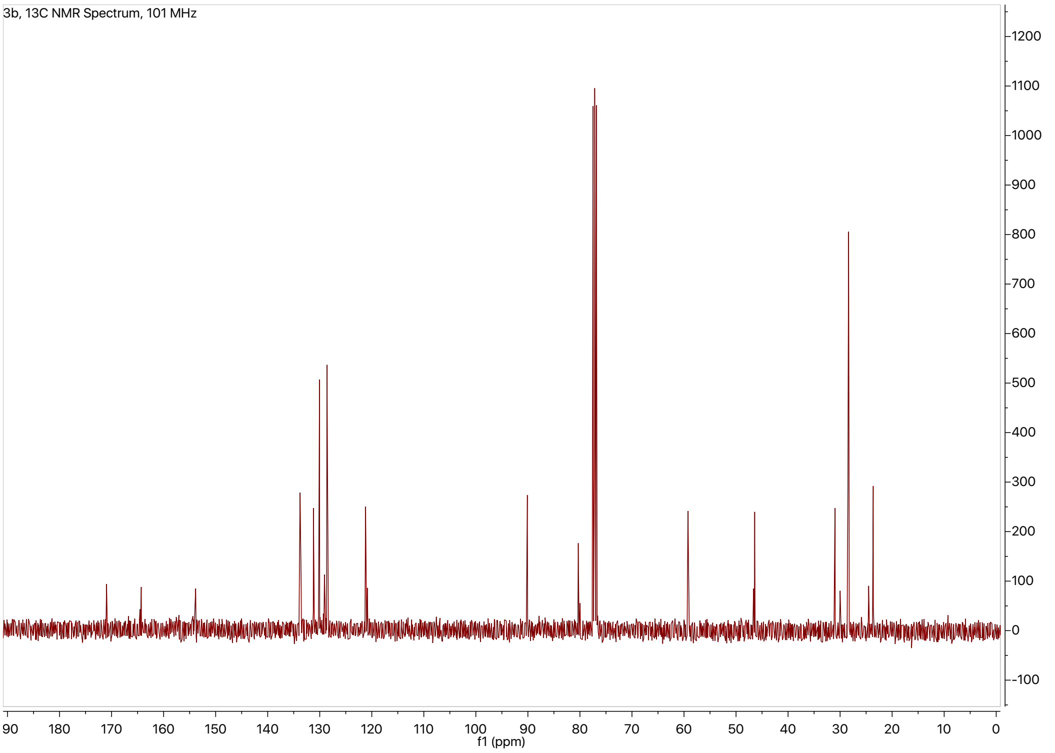

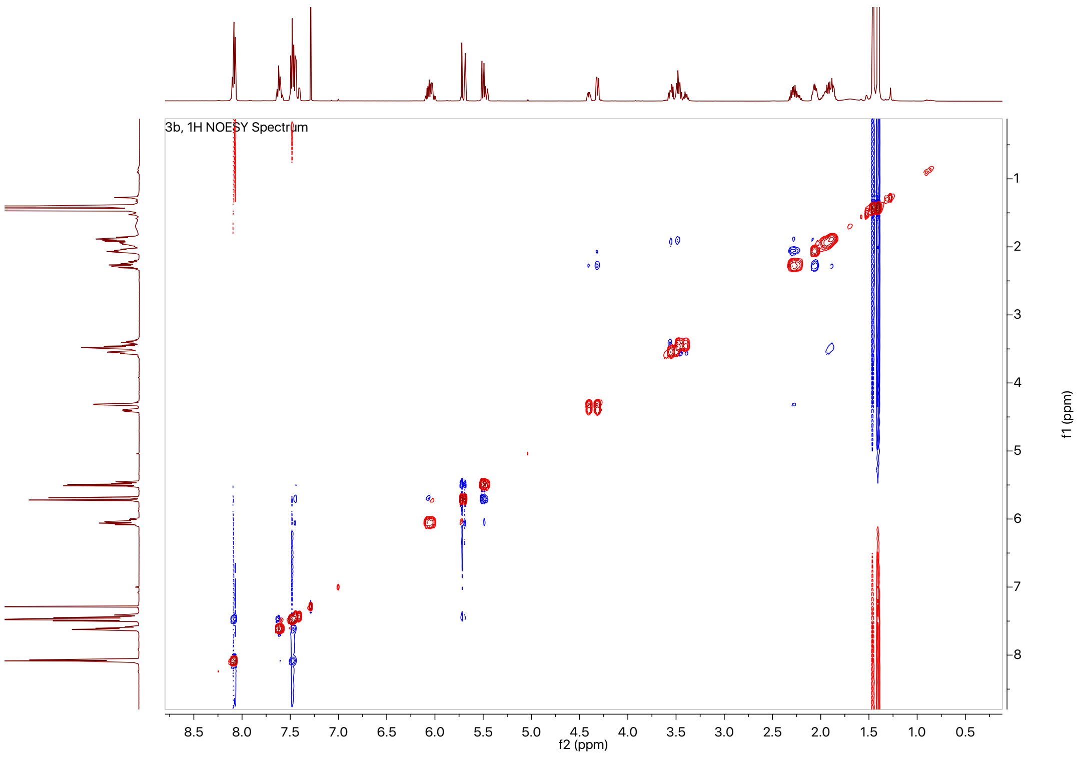

Zoomed in from 3.00 – 4.70 ppm

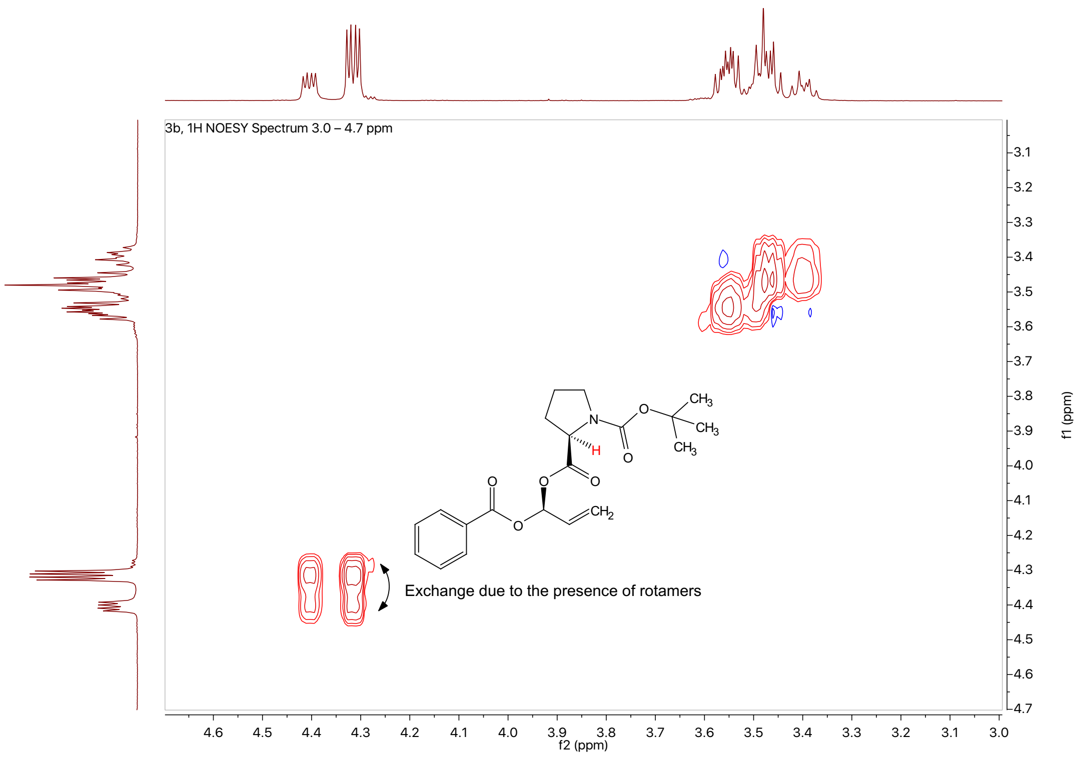

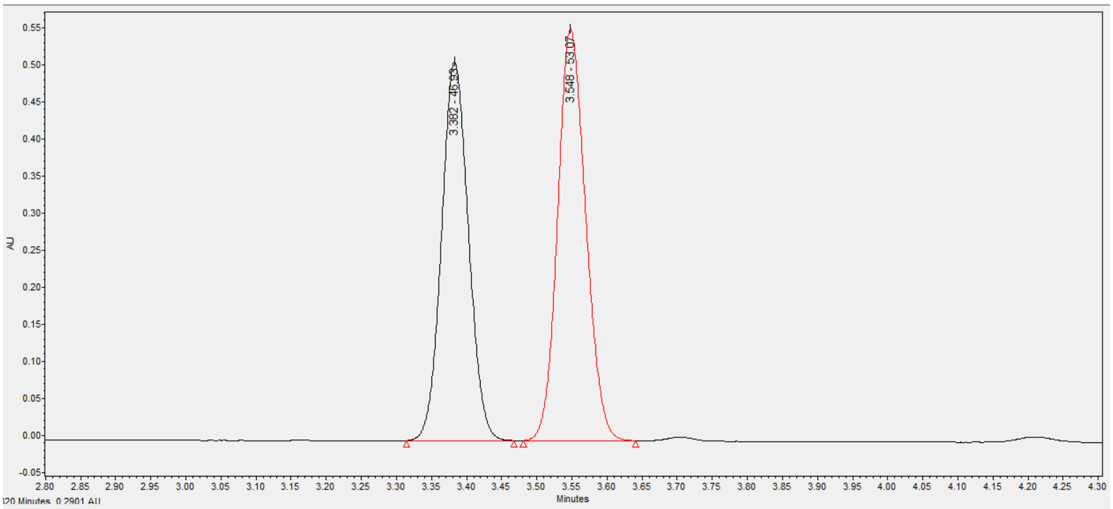

AU
Minutes
3.382 - 46.93
3.548 - 53.07

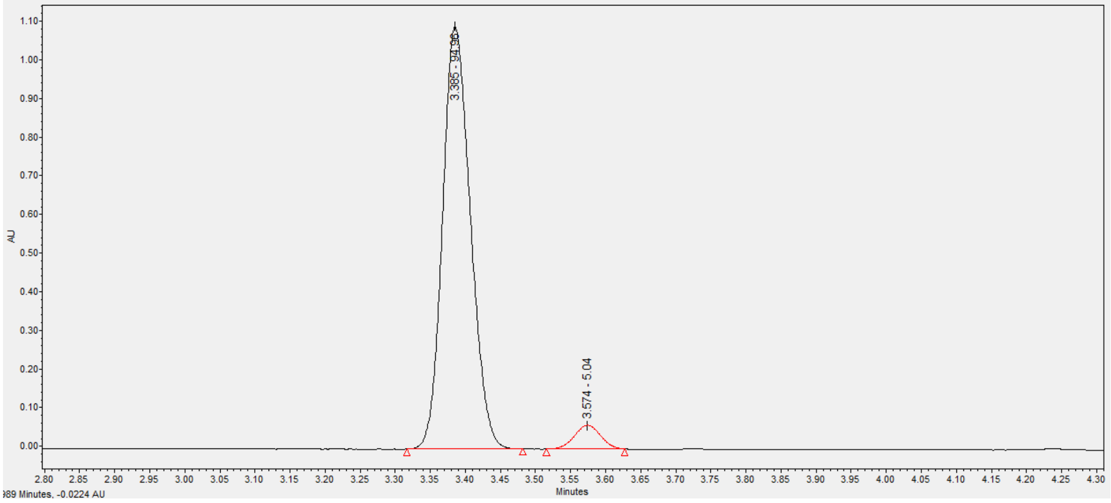

AU
Minutes
3.385 - 94.96
3.574 - 5.04
989 Minutes, -0.0224 AU

Supplementary Figure 28: ^{1}H, ^{13}C NMR and SFC trace for **3d**

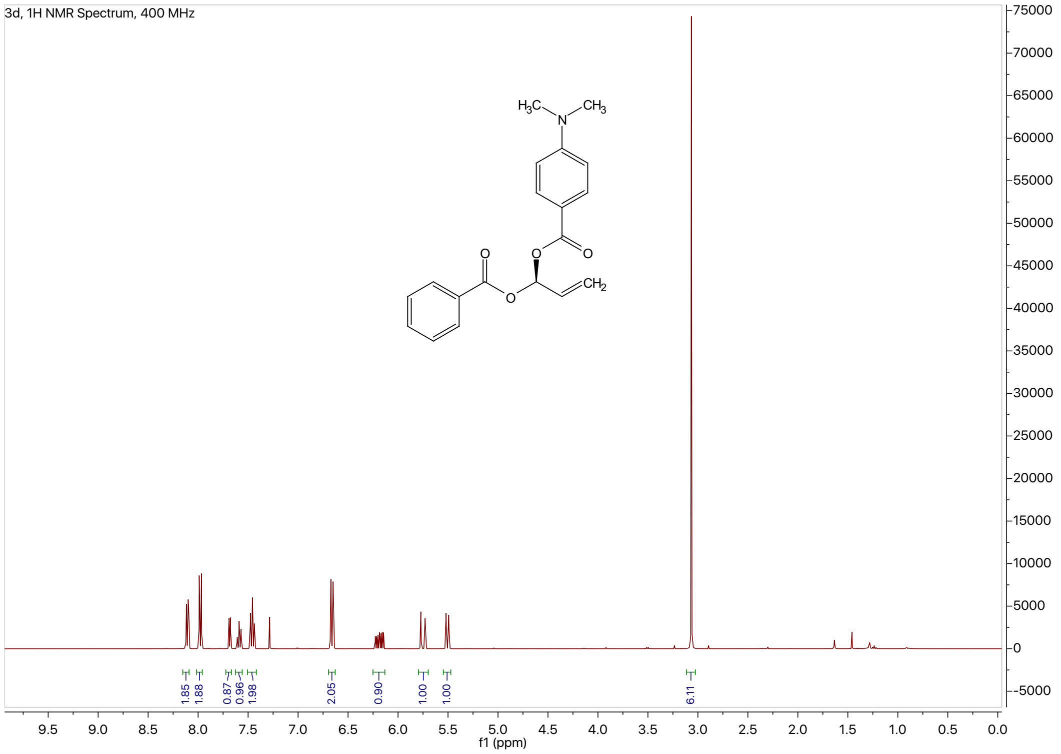

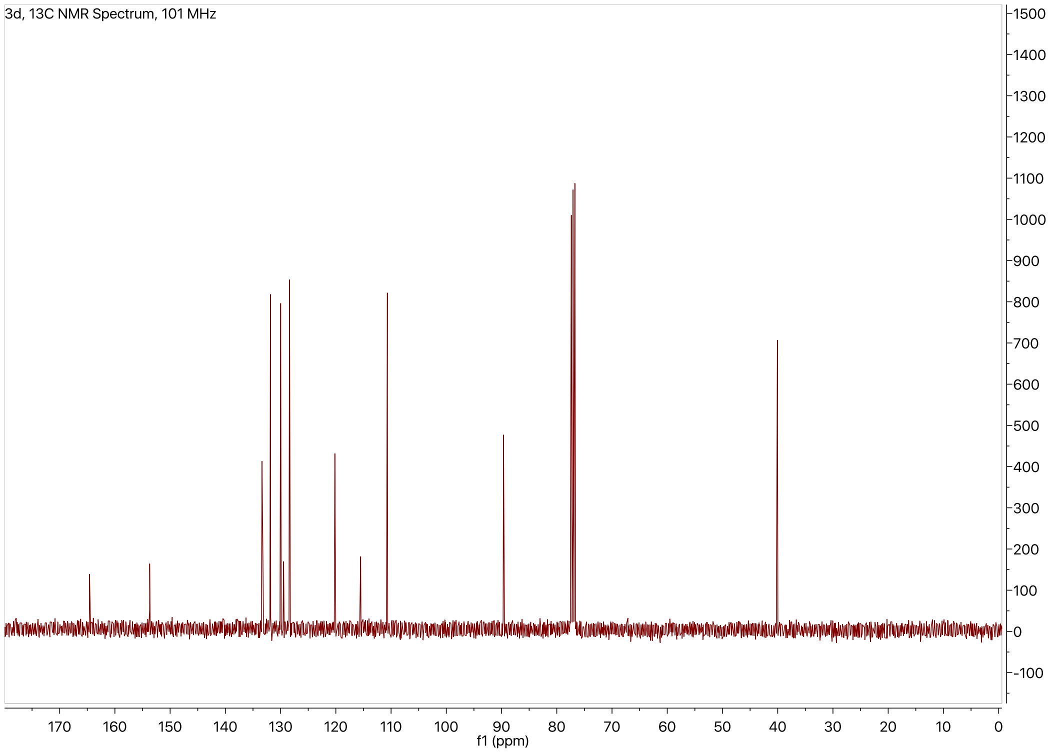

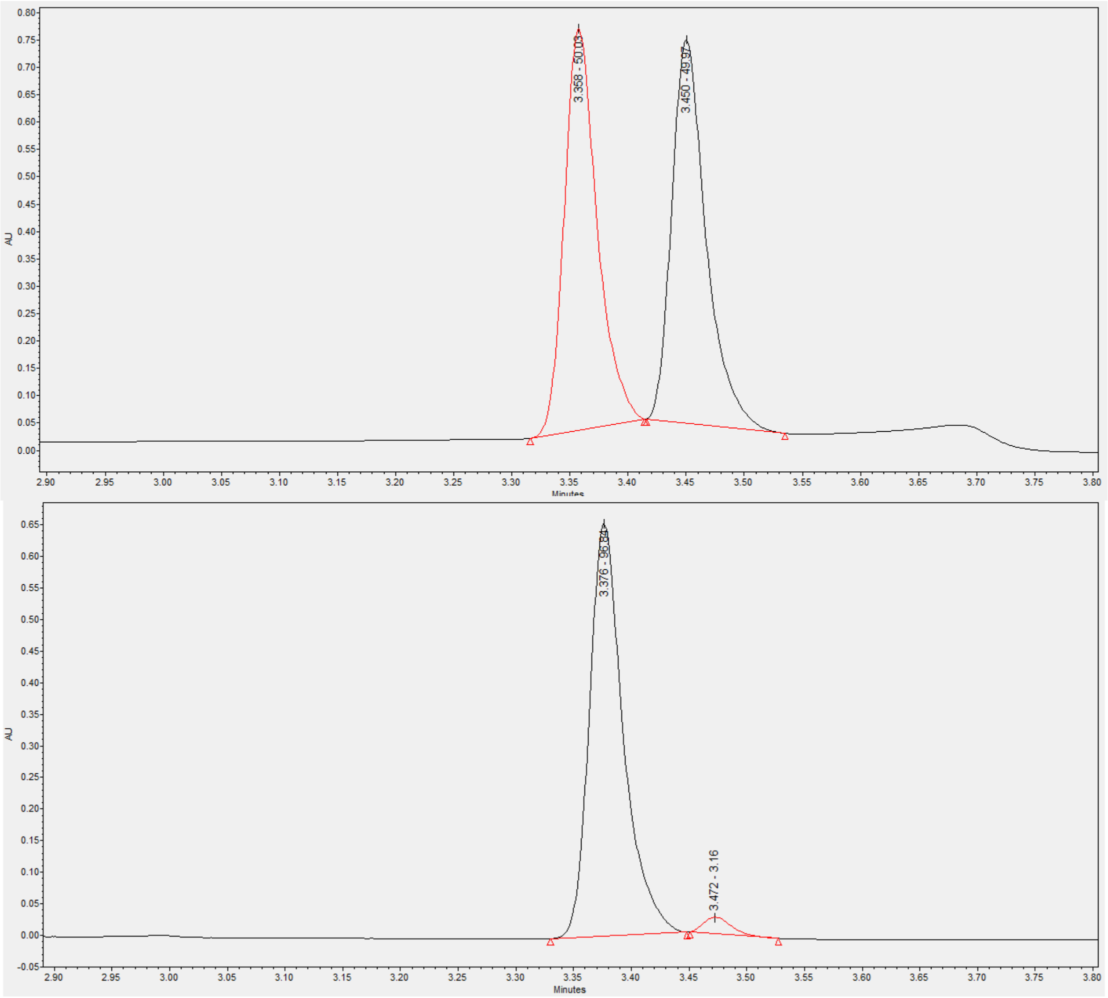

Supplementary Figure 29: ^{1}H, ^{13}C NMR and SFC trace for **3f**

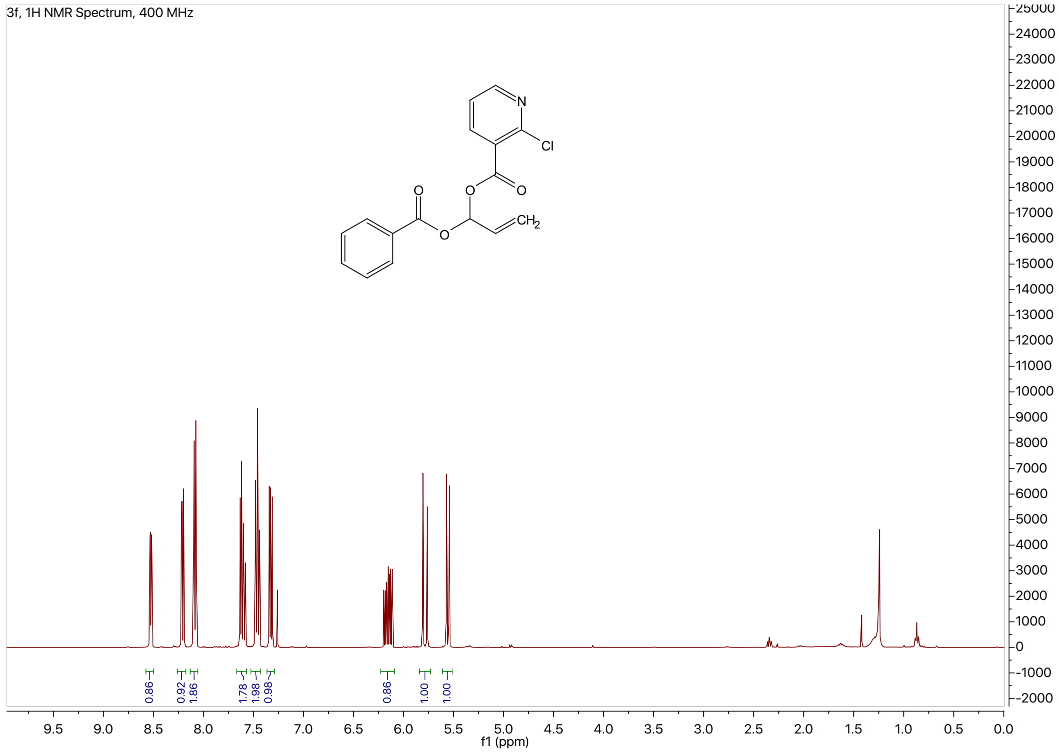

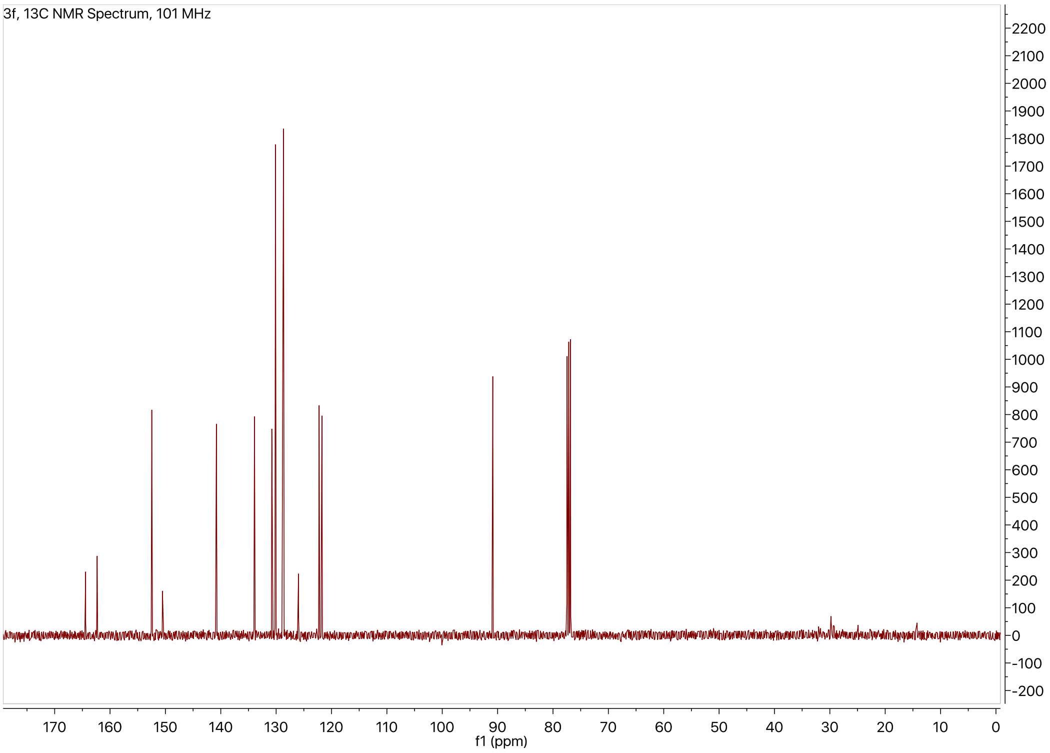

3f, 13C NMR Spectrum, 101 MHz
f1 (ppm)

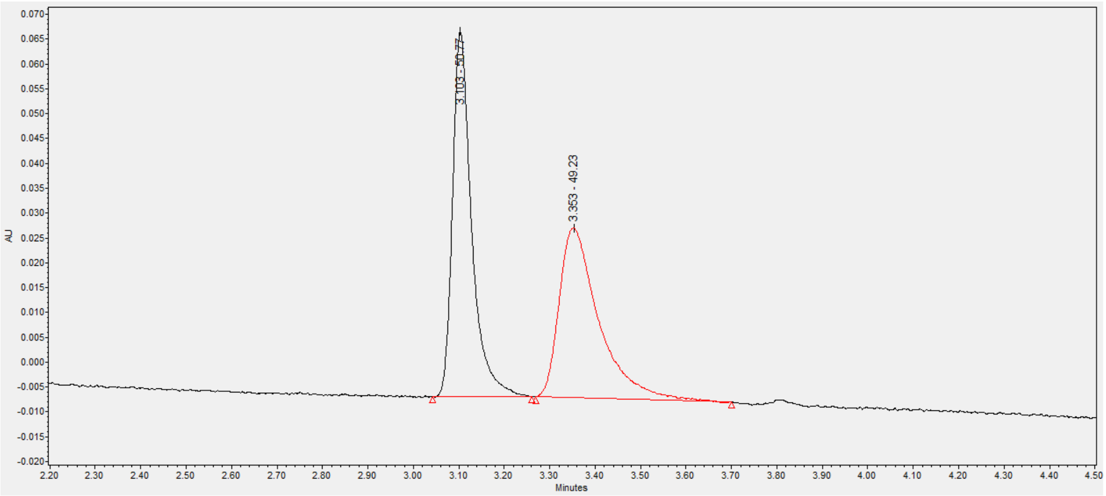

3.103 - 50.77
3.353 - 49.23
AU
Minutes

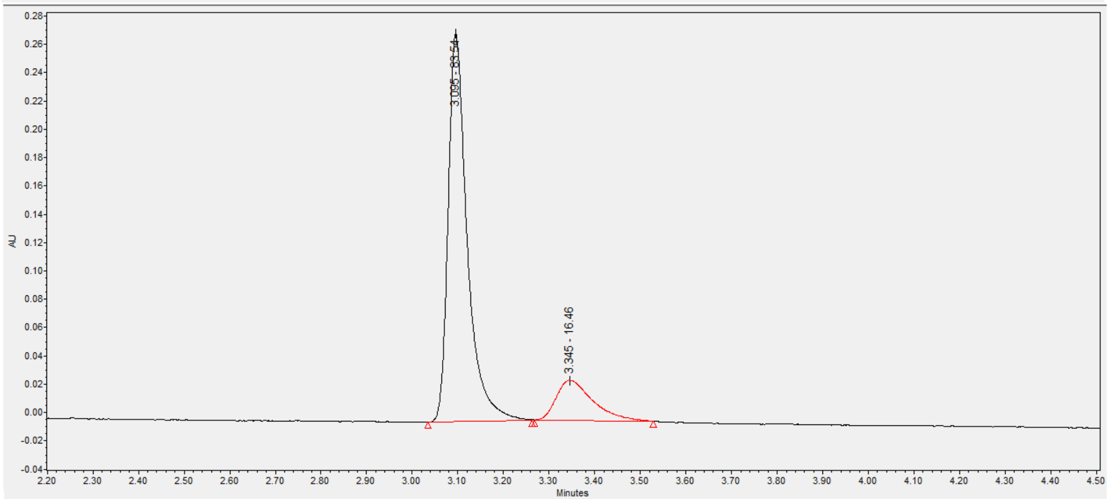

3.096 - 83.54
3.345 - 16.46
AU
Minutes

Supplementary Figure 30: ^{1}H, ^{13}C NMR and SFC trace for **3g**

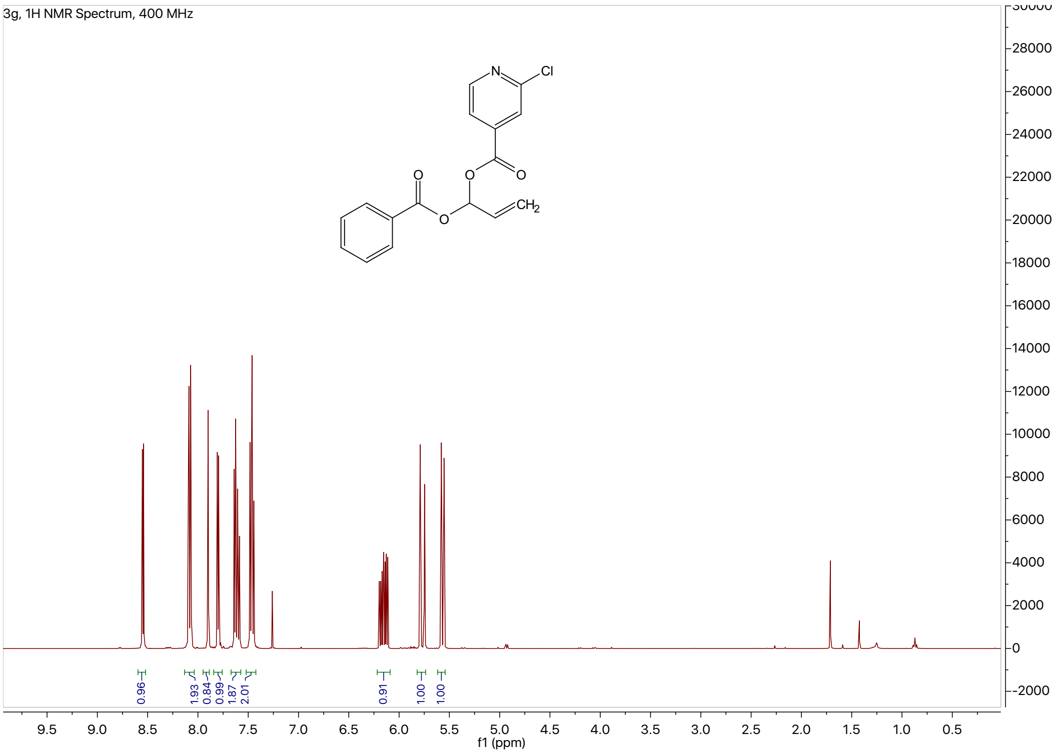

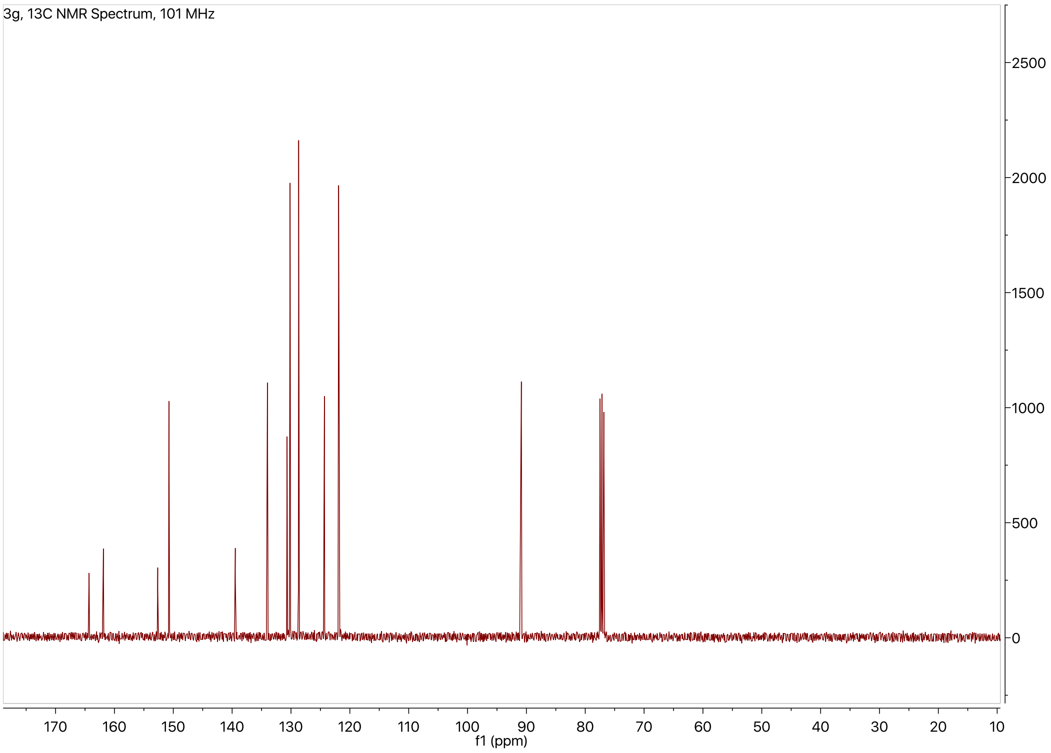

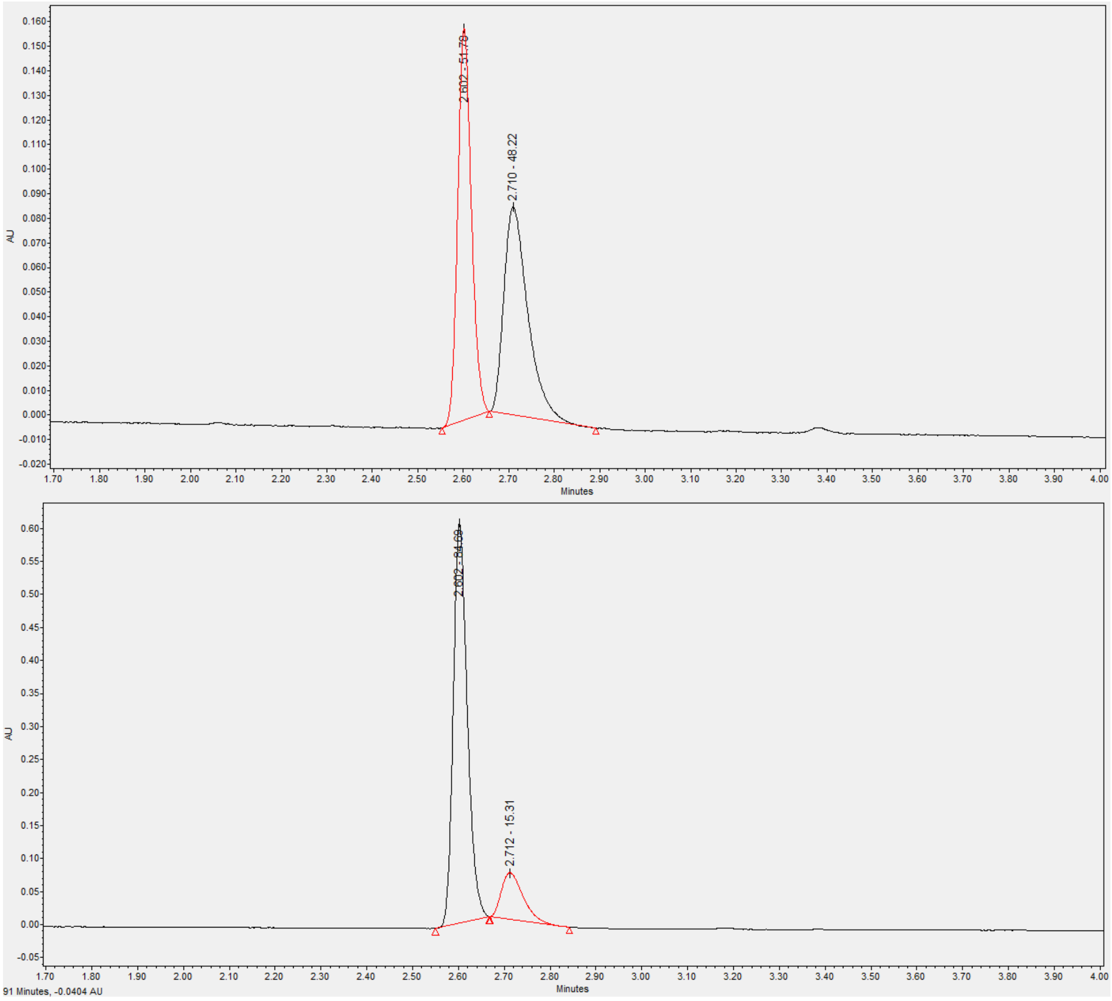

Supplementary Figure 31: ^{1}H, ^{13}C NMR and SFC trace for **4a**

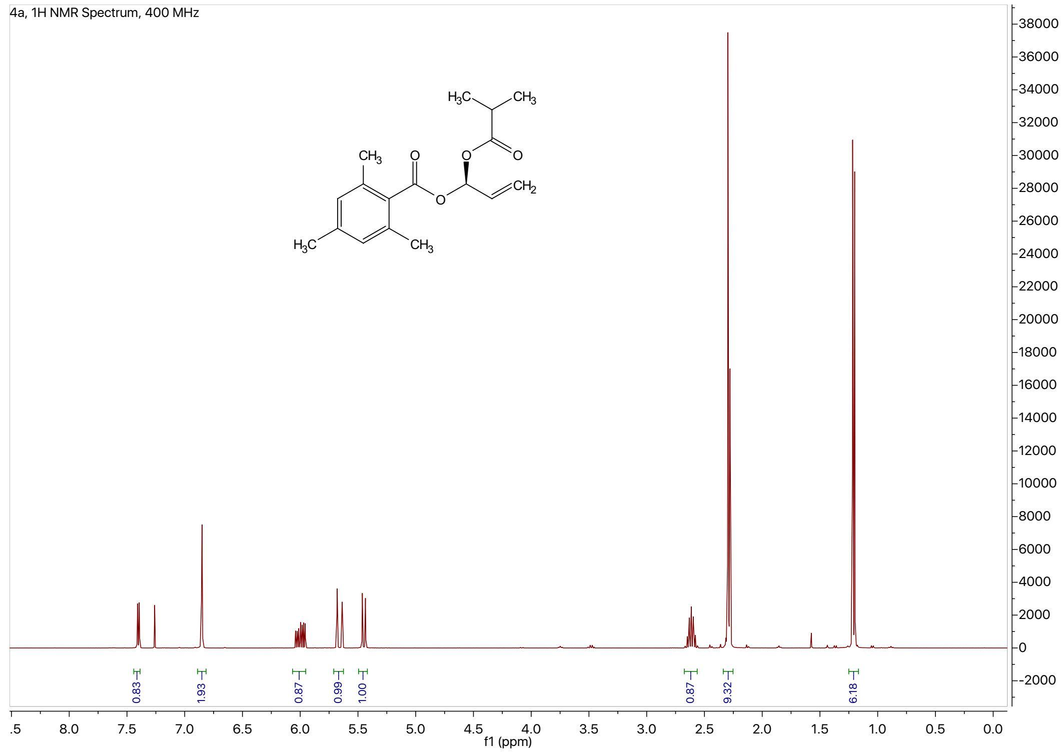

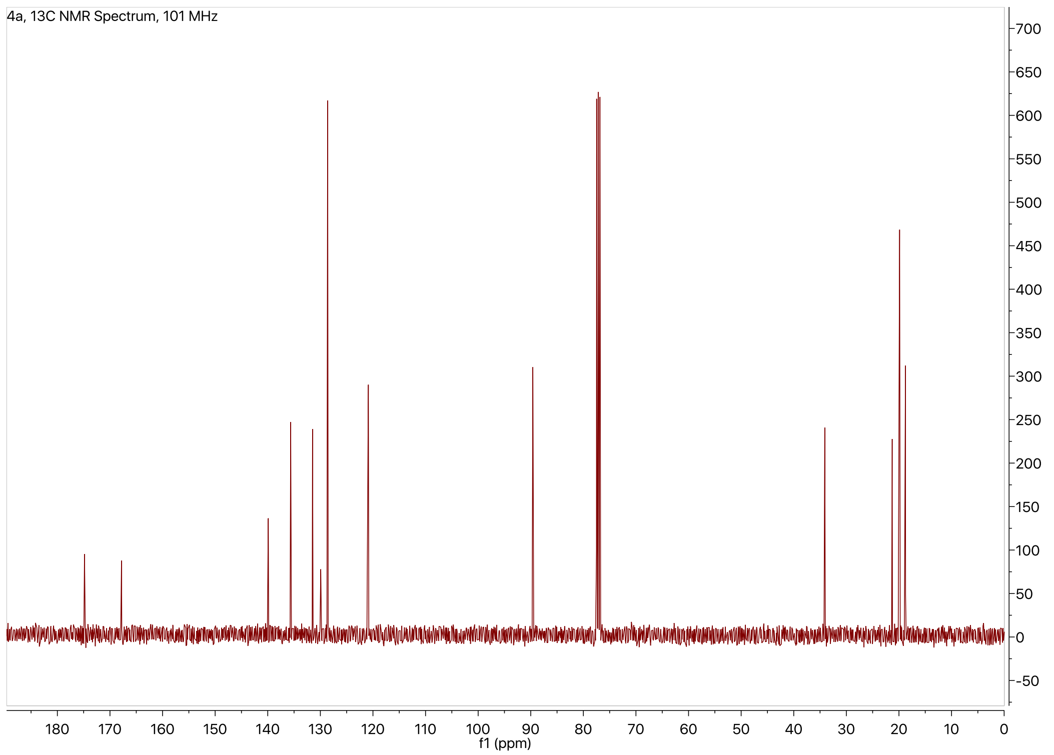
4a, 13C NMR Spectrum, 101 MHz
f1 (ppm)

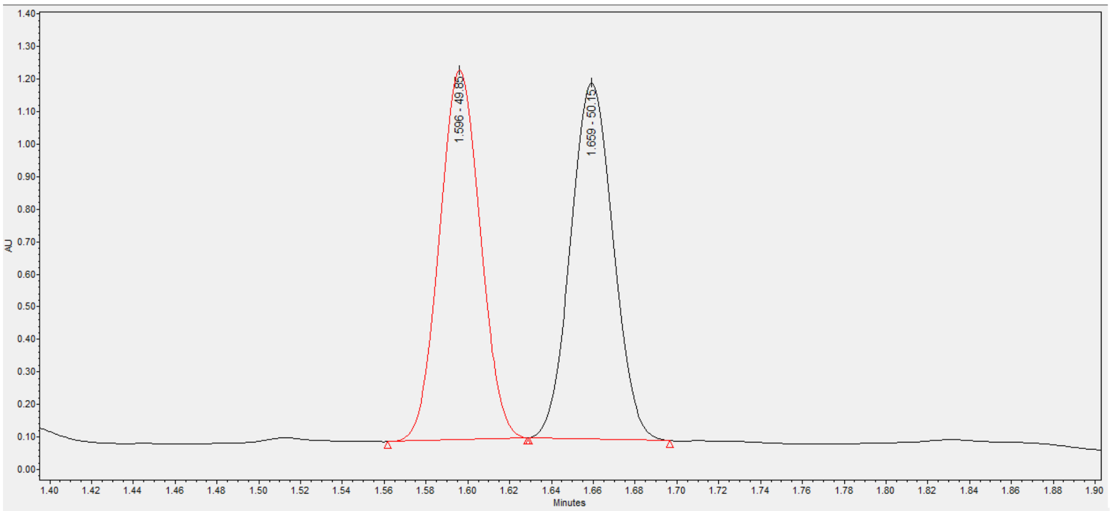
1.596 - 49.85
1.659 - 50.15
AU
Minutes

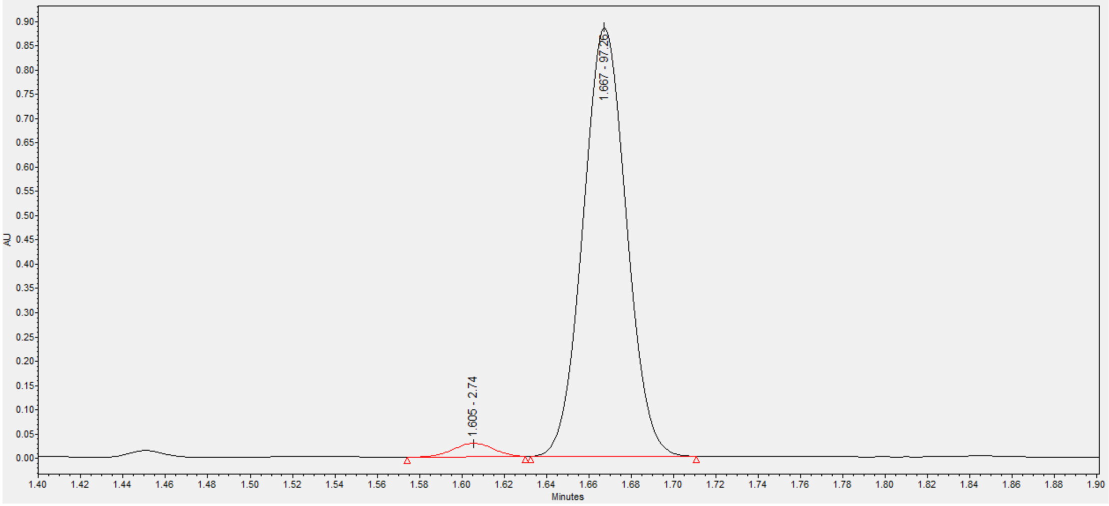
1.605 - 2.74
1.667 - 97.26
AU
Minutes

Supplementary Figure 32: ^{1}H, ^{13}C NMR and SFC trace for **4b**

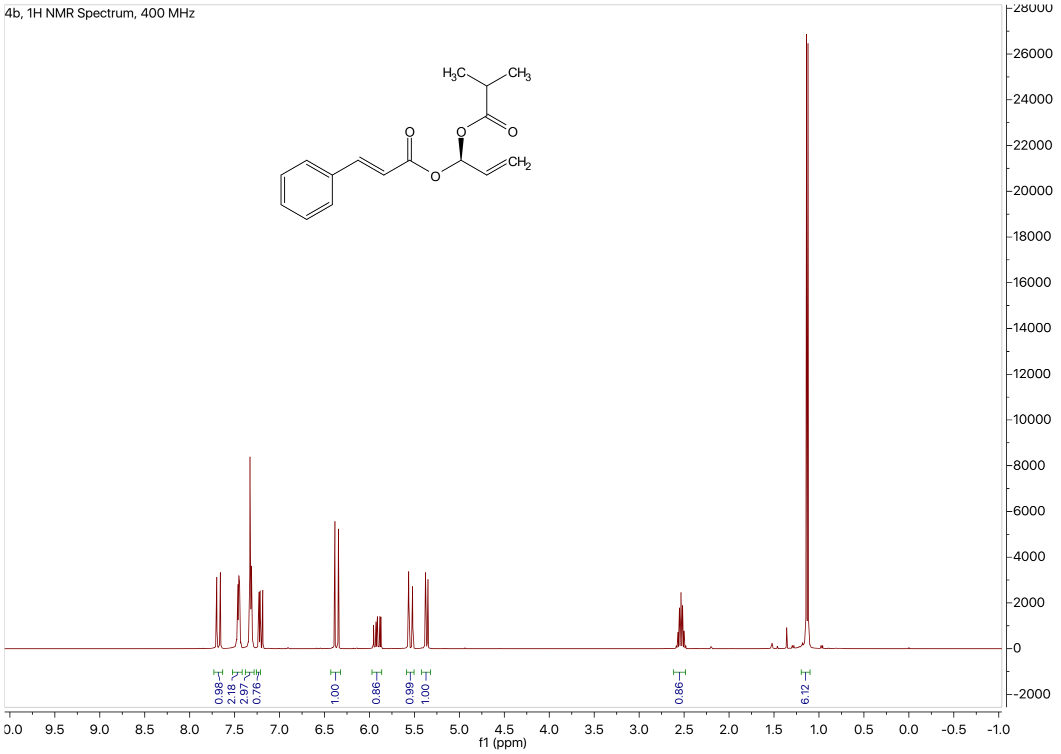

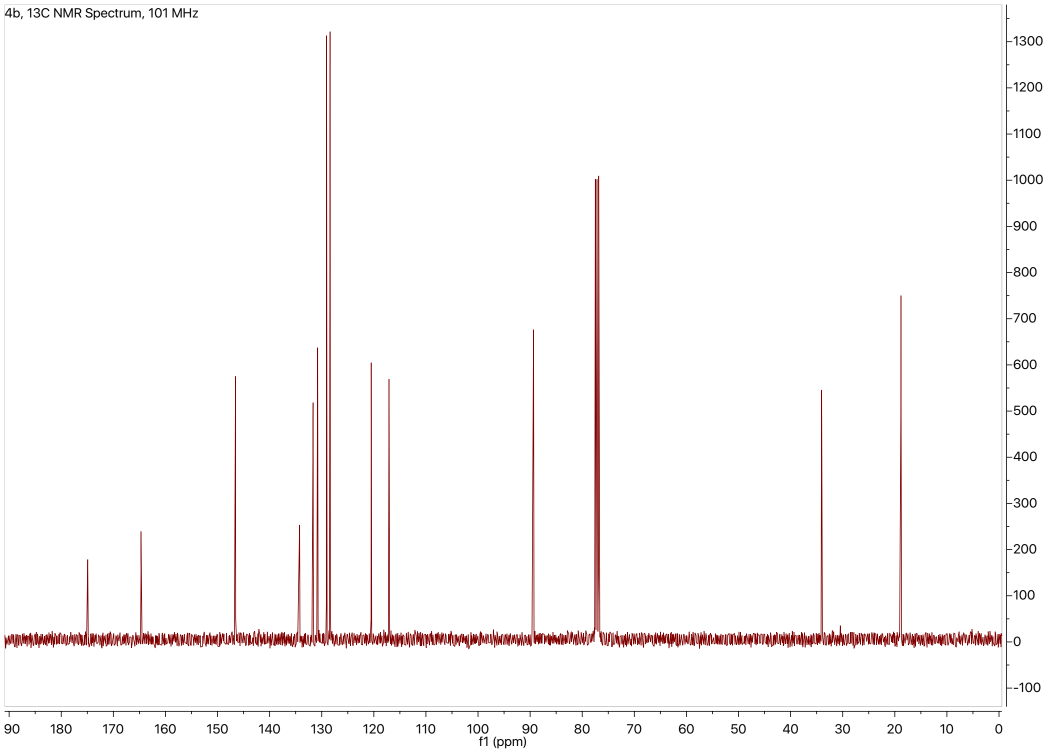

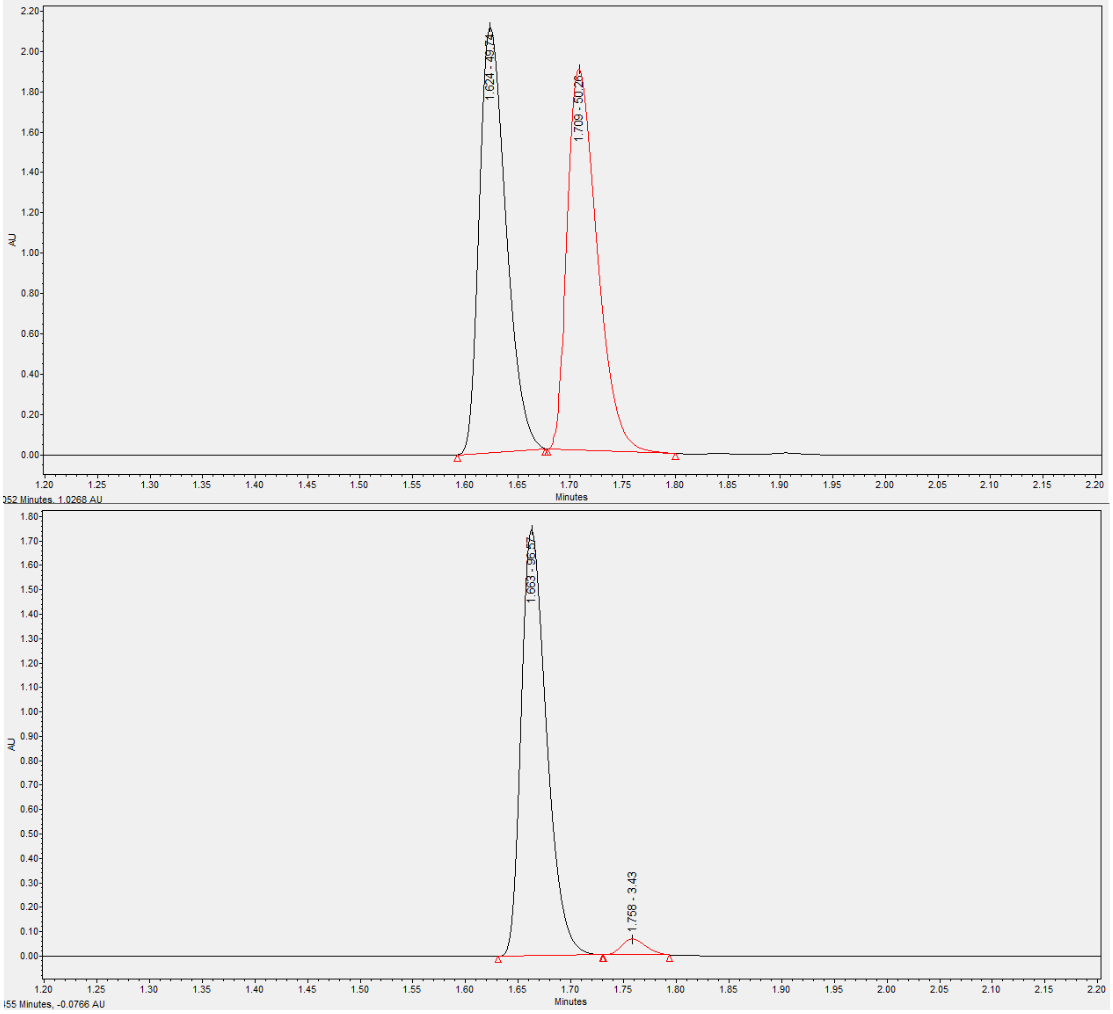

Supplementary Figure 33: ^{1}H, ^{13}C NMR and SFC trace for **4c**

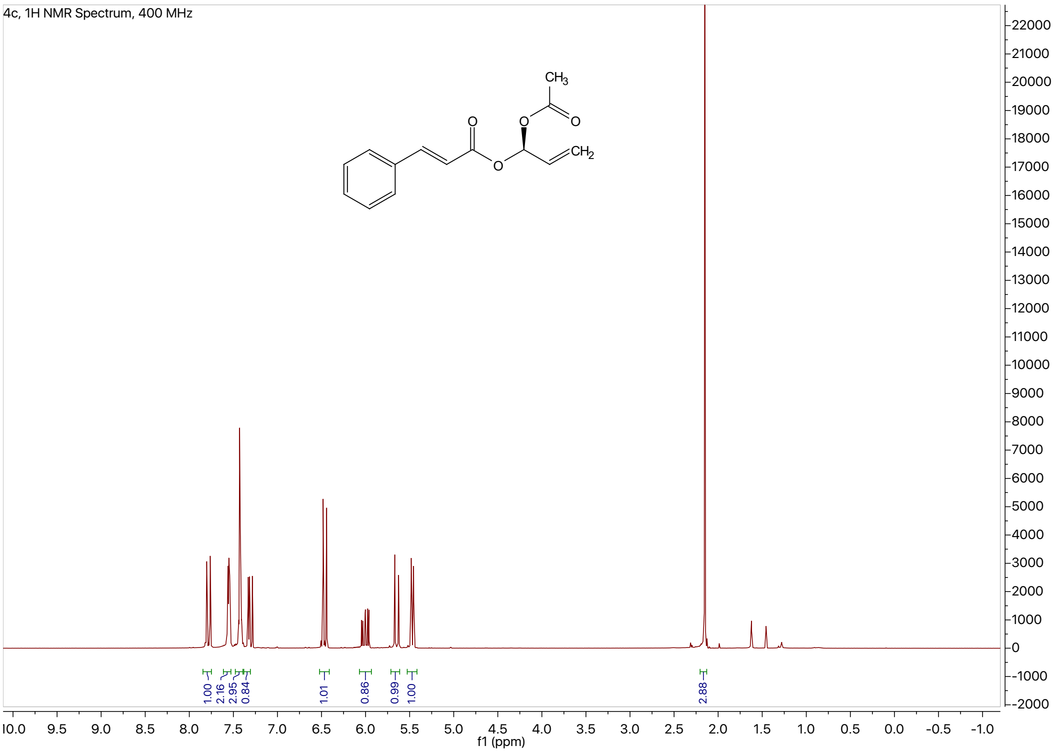

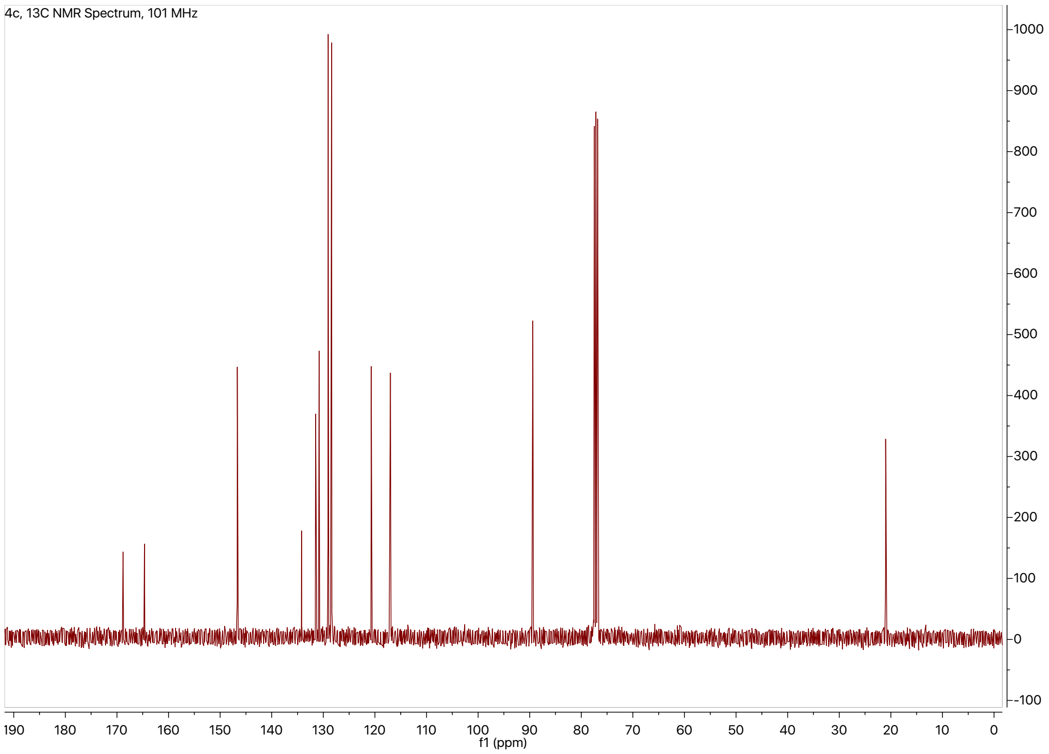
4c, 13C NMR Spectrum, 101 MHz
f1 (ppm)

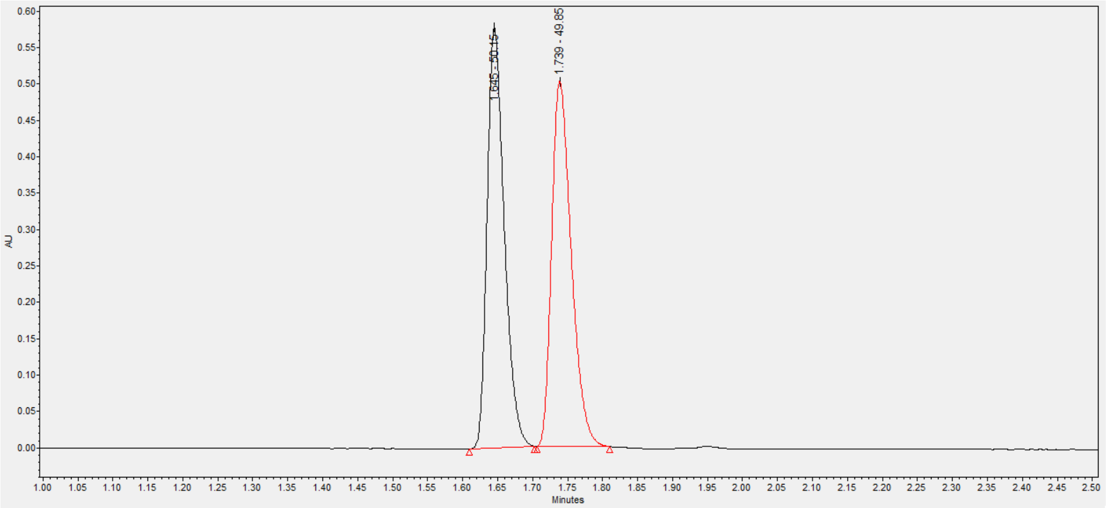
1.645 - 50.15
1.739 - 49.85
AU
Minutes

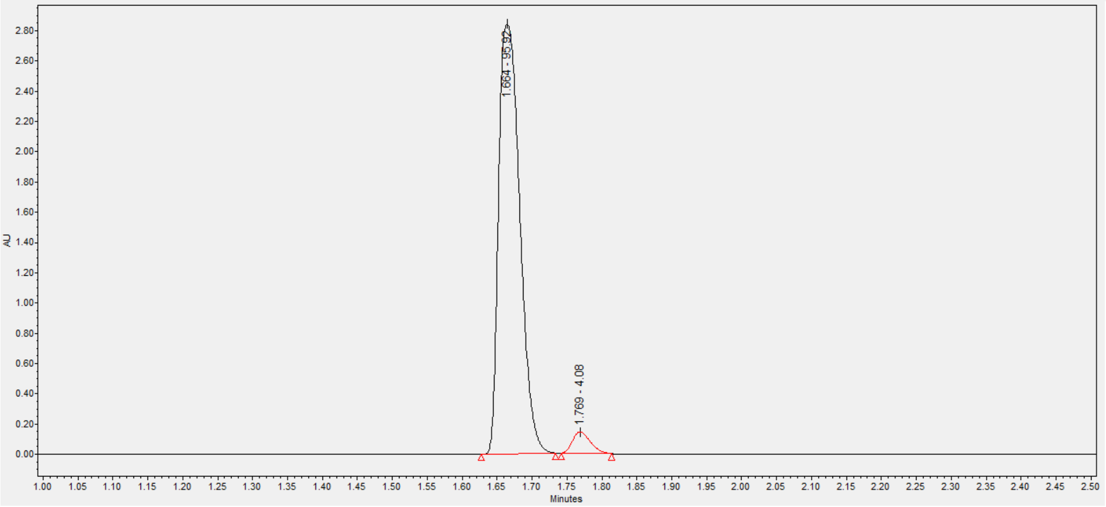
1.664 - 95.92
1.769 - 4.08
AU
Minutes

Supplementary Figure 34: ^{1}H, ^{13}C NMR and SFC trace for **5a**

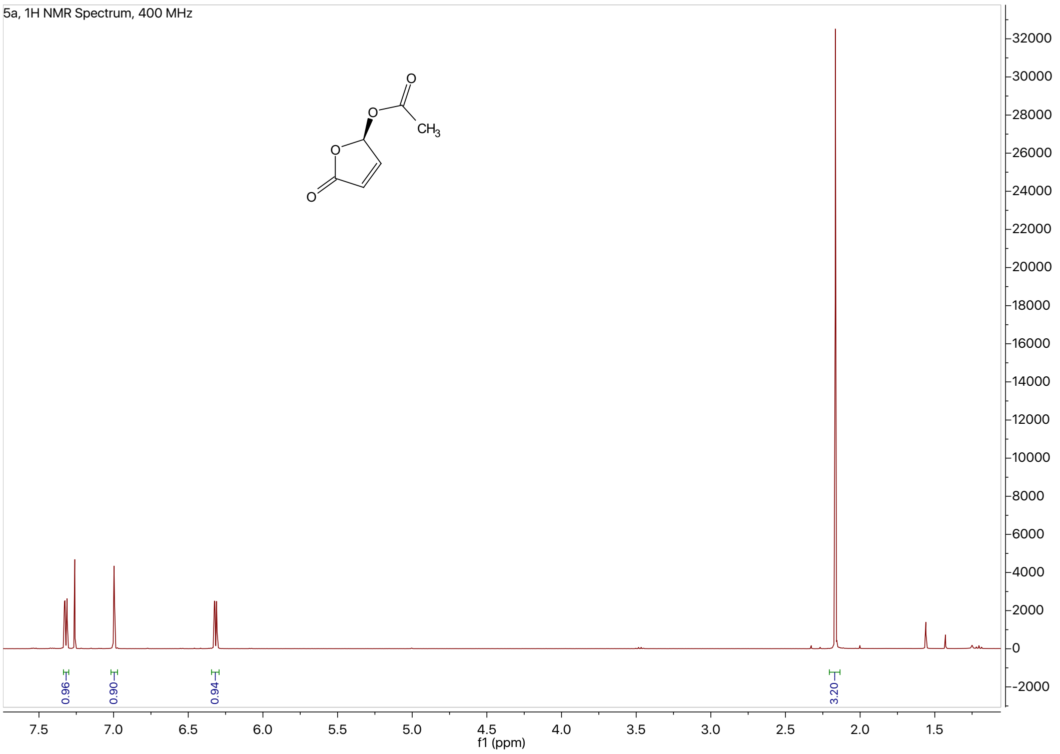

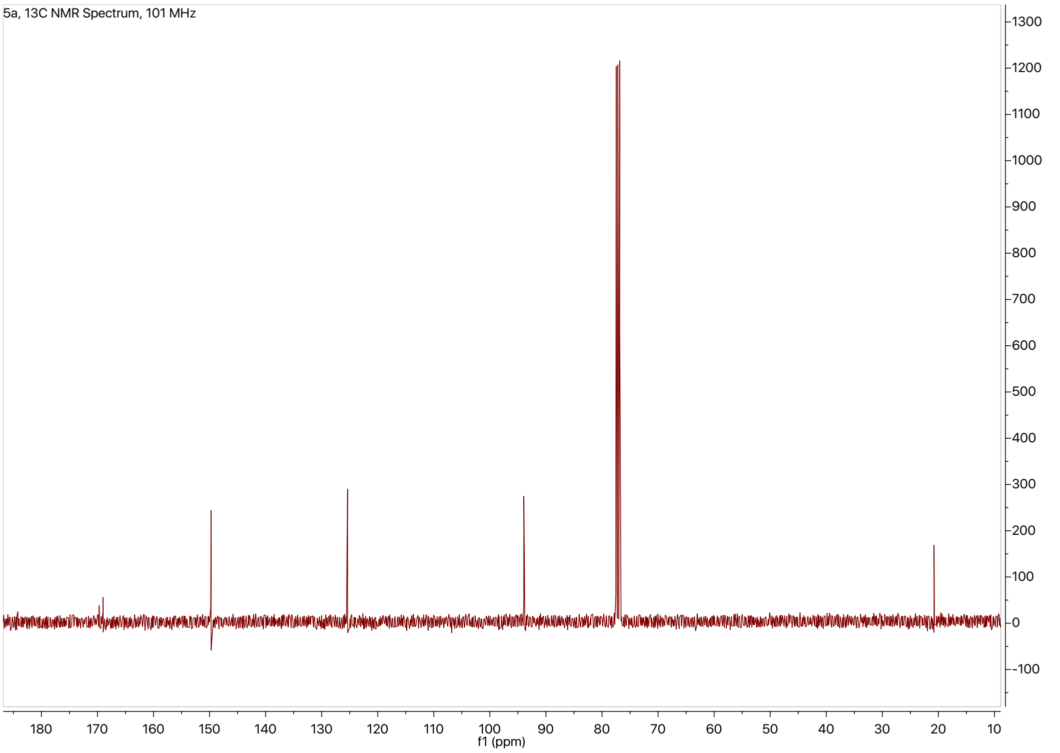

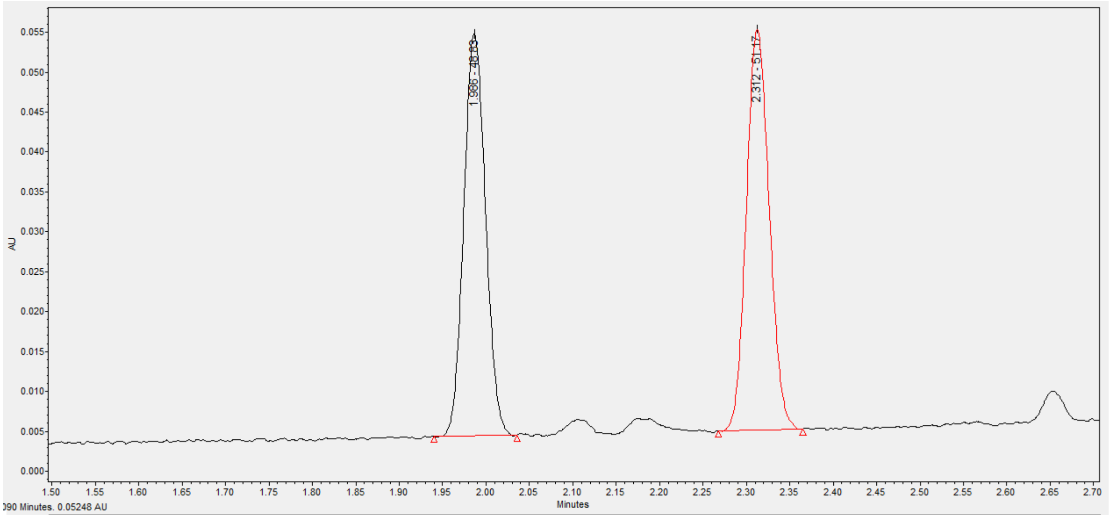

1.986 - 48.83
2.312 - 51.17
AU
Minutes
090 Minutes. 0.05248 AU

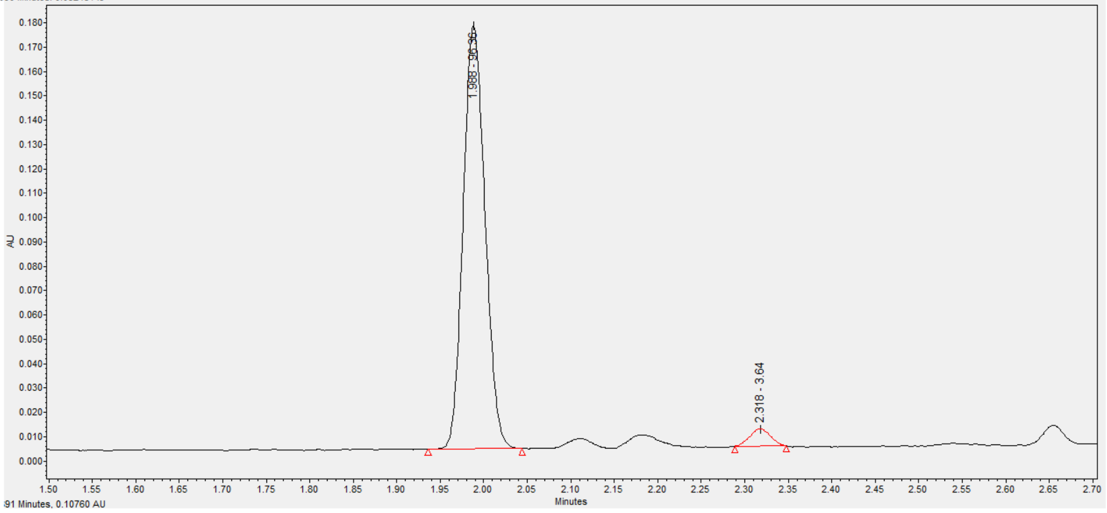

1.988 - 96.36
2.318 - 3.64
AU
Minutes
91 Minutes, 0.10760 AU

Supplementary Figure 35: ^{1}H, ^{13}C NMR and SFC trace for **5b**

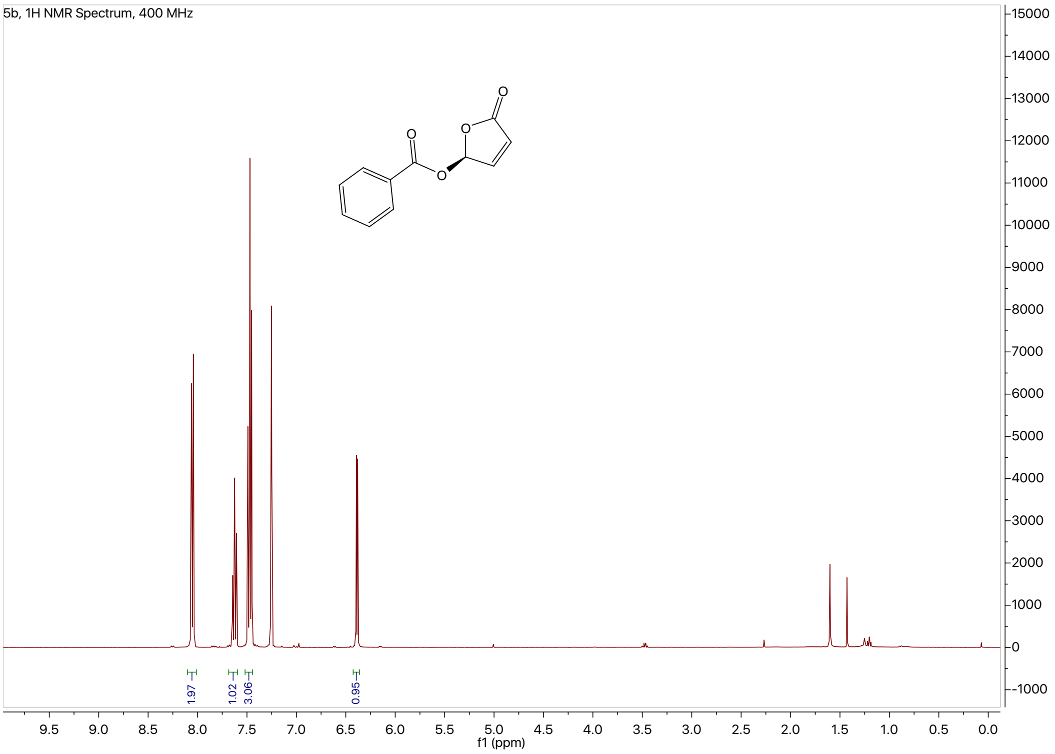

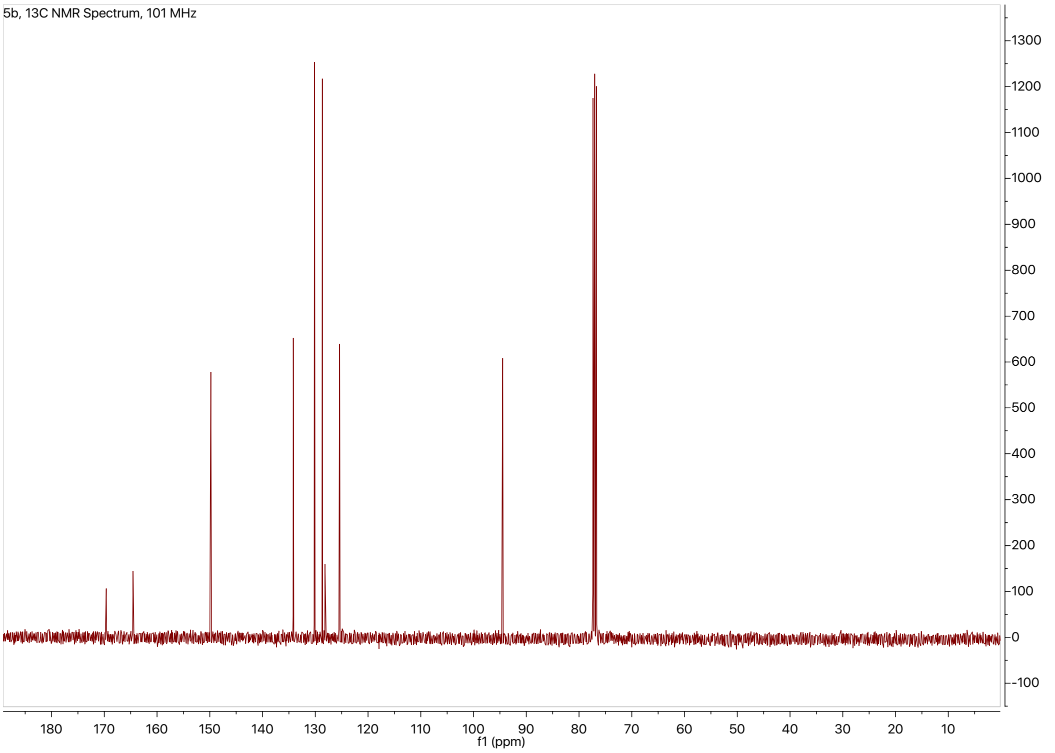

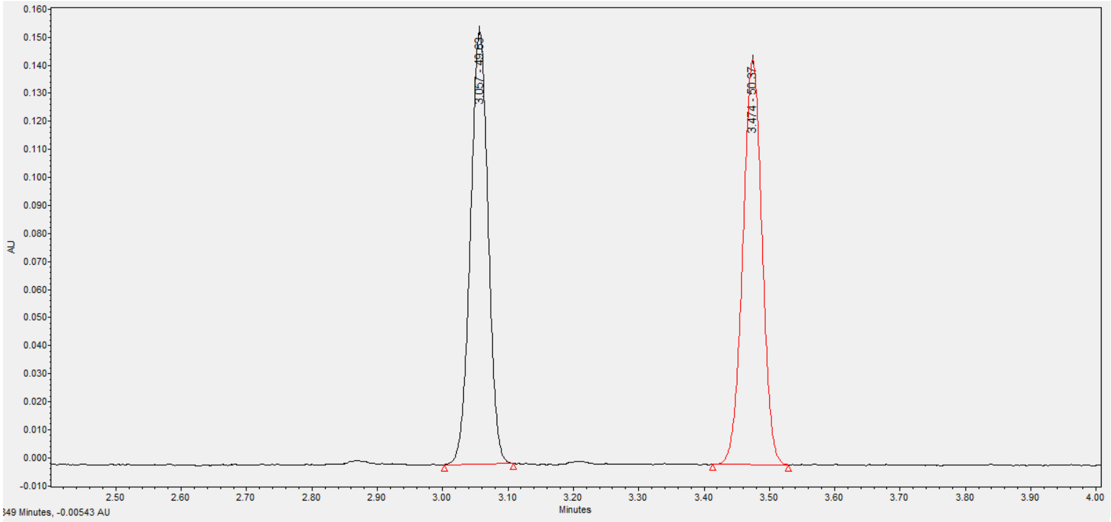
3.057 - 49.63
3.474 - 50.37
AU
Minutes
349 Minutes, -0.00543 AU

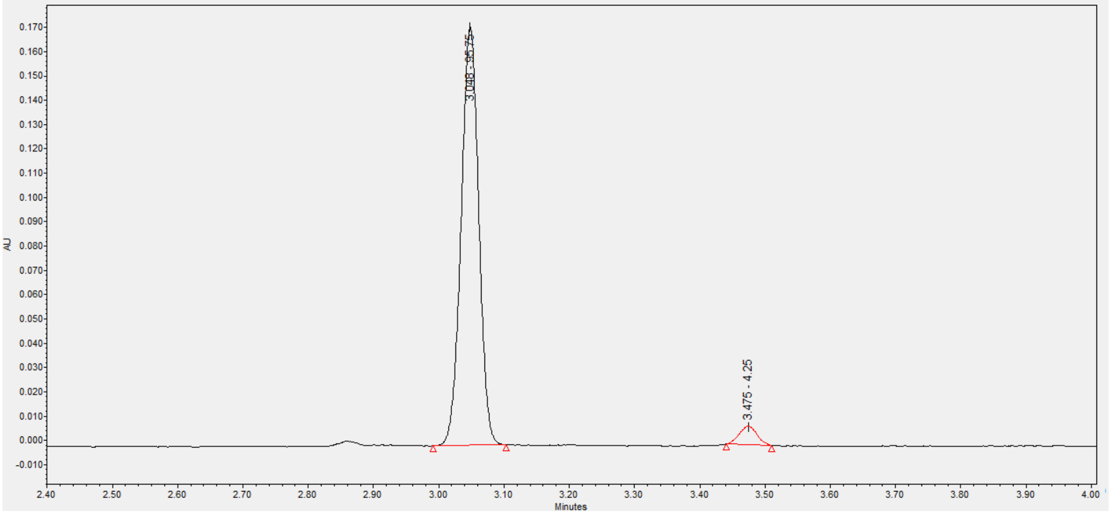
3.048 - 95.75
3.475 - 4.25
AU
Minutes

Supplementary Figure 36: ^{1}H, ^{13}C NMR and SFC trace for **5c**

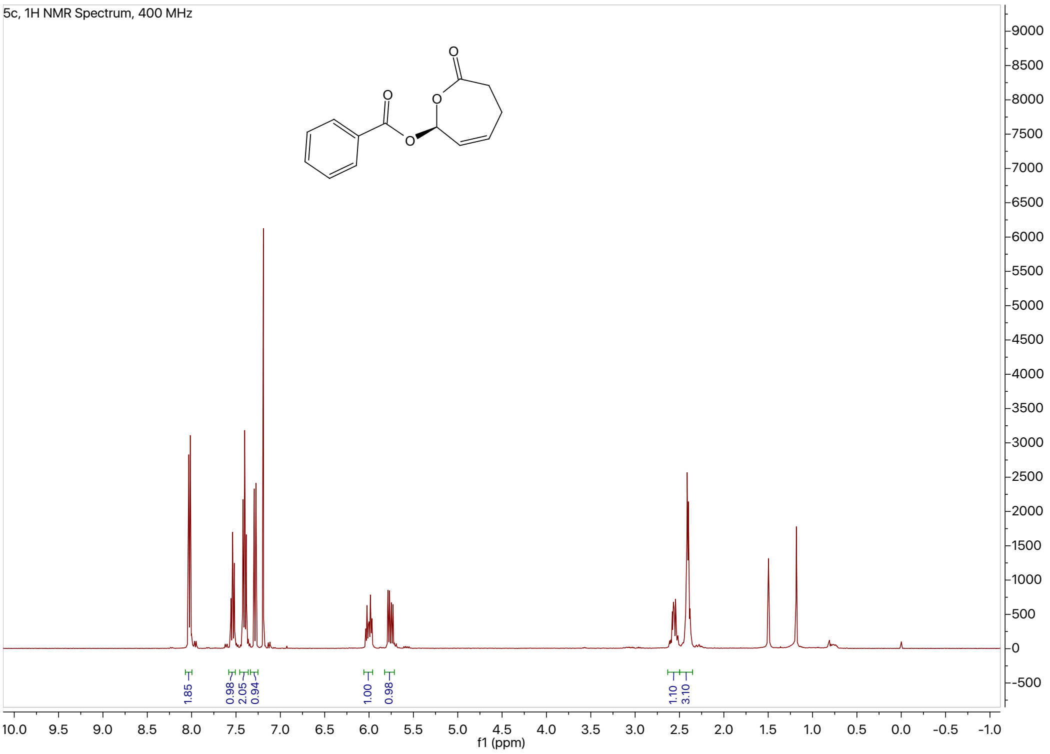

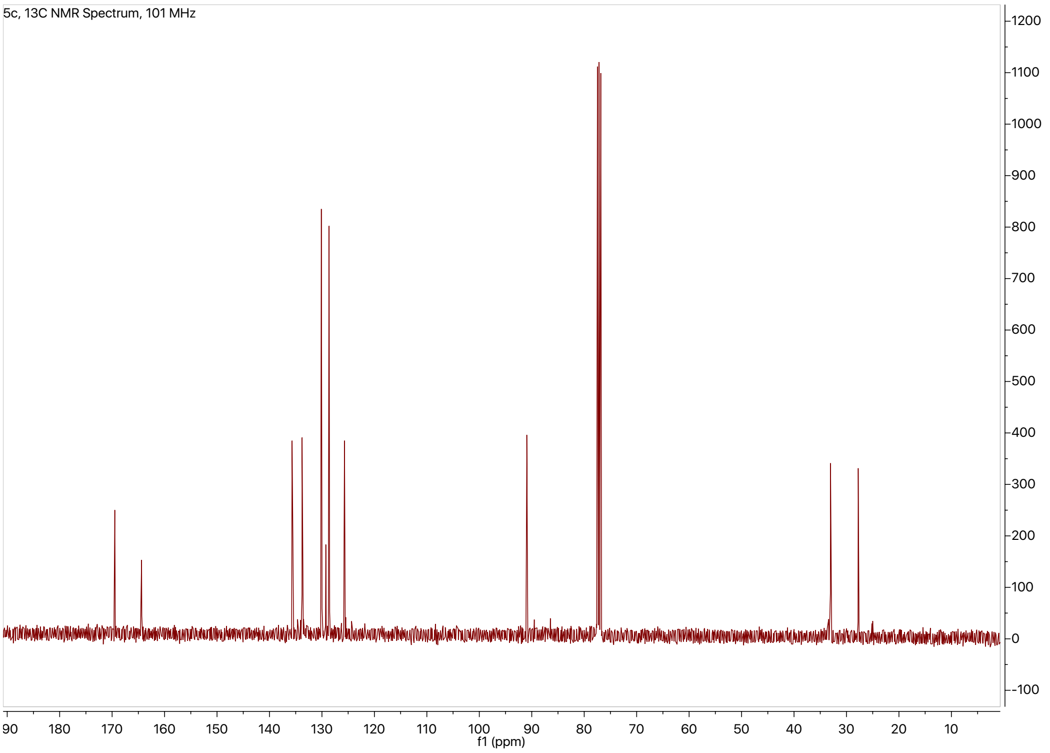

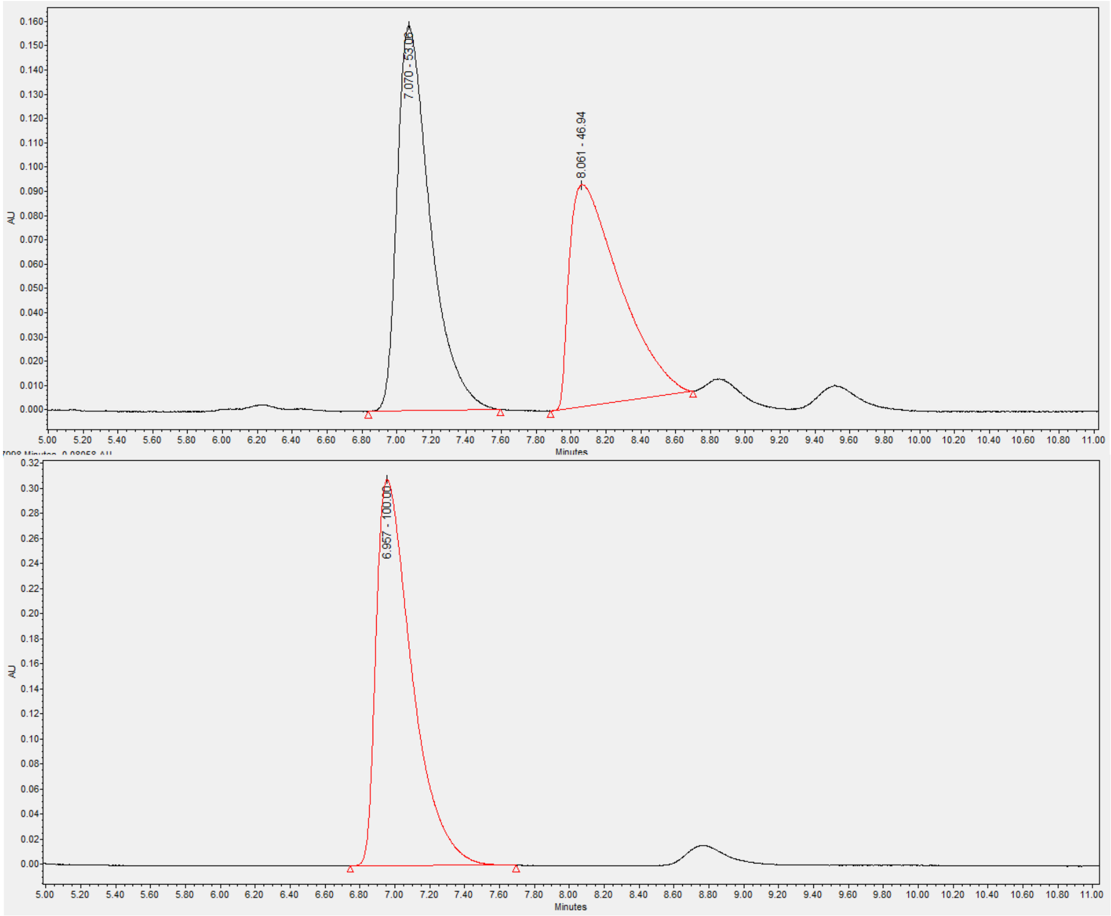
7.070 - 53.06
8.061 - 46.94
AU
Minutes
6.957 - 100.00
AU
Minutes
